# Clinical Efficacy of Single Application Local Drug Delivery and Adjunctive Agents in Nonsurgical Periodontal Therapy: A Systematic Review and Network Meta-Analysis

**DOI:** 10.3390/pharmaceutics12111086

**Published:** 2020-11-12

**Authors:** Oi Leng Tan, Syarida Hasnur Safii, Masfueh Razali

**Affiliations:** 1 Centre for Restorative Dentistry, Unit of Periodontology, Faculty of Dentistry, National University of Malaysia, Jalan Raja Muda Abdul Aziz, Kuala Lumpur 50300, Malaysia; oileng1086@gmail.com; 2 Department of Restorative Dentistry, Faculty of Dentistry, University of Malaya, Kuala Lumpur 50603, Malaysia; syarida.safii@um.edu.my

**Keywords:** evidence-based dentistry, anti-bacterial agents, periodontal debridement, periodontal pocket, periodontitis, meta-analysis, photochemotherapy, anti-infective agents, local

## Abstract

This review aimed to rank the clinical efficacy of commercially available single-application local drug delivery and adjunctive agents (LDAs) compared with subgingival mechanical debridement (SMD) in nonsurgical periodontal therapy (NSPT). Randomized controlled clinical trials that compared LDAs against SMD alone or with placebo in adults (aged at least 18 years) diagnosed with periodontitis with a minimum of 6 months follow-up were included. A frequentist approach to random-effects network meta-analysis was implemented. The efficacies of the LDAs measured by probing pocket depth (PPD) reduction and clinical attachment level (CAL) gain were reported as mean difference (MD) with 95% confidence intervals (CIs). The treatments were ranked according to their P-score. Four network meta-analyses suggested that sulfonic/sulfuric acid gel (PPD MD −1.13 mm, 95% CI −1.74 to −0.53, P-score 0.91; CAL MD −1.09 mm, 95% CI −1.58 to −0.61, P-score 0.95) and doxycycline hyclate gel (PPD MD −0.90 mm, 95% CI −1.50 to −0.30, P-score 0.93; CAL MD −0.84 mm, 95% CI −1.40 to −0.28, P-score 0.92) were the most effective in reducing PPD and gaining CAL in split-mouth and parallel studies, respectively (moderate certainty of evidence). LDAs have differing efficacies, but they present with possible clinical significance over SMD alone in NSPT.

## 1. Introduction

According to the 2019 Global Burden of Disease Study [1], periodontal disease has affected an estimated 1.06 billion people worldwide and can be an economic burden to a nation [2,3,4]. The incidence of periodontitis is likely to increase with the rising prevalence of diabetes, which is projected to affect 578 million people (10.2%) by 2030 [5] and tobacco smoking, in which 18.9% of the global population are current tobacco smokers [6]. Both factors are established risks for periodontitis [7]. Untreated periodontitis may ultimately progress to tooth loss, which can remarkably impact affected individuals functionally, aesthetically, and physiologically [8,9].

Nonsurgical mechanical periodontal debridement has been established as the gold standard in treating periodontitis with probing depth reductions and attachment level gains of up to 2 mm in moderate to deep pockets [10]. However, this approach has its limitations. Restricted access to furcation areas, deep pockets, and irregular surfaces complicate the removal of bacterial deposits, and the procedure is vastly dependent on the skills of the clinician [11].

Local adjunctive periodontal therapy has received much attention, as periodontal infections are more likely to be confined within the periodontal pocket. Furthermore, potential side effects and increased antibiotics resistance from systemic antimicrobials use can be prevented with local adjuncts [12]. There has been extensive research on other local pharmacologic approaches such as probiotics [13], phytotherapy [14,15,16], and also host-modulation therapy [17]. A recent market research report [18] projected that the global periodontal therapeutics market would expand to US$270.7 million by the year 2026 at a compound annual growth rate of 7.4%. Local antimicrobials have been utilized as an adjunct to the treatment of periodontitis for four decades [19,20], of which certain adjuncts are more effective than others [21,22,23,24,25,26]. The latest systematic review and meta-analyses that focused only on local antimicrobials adjuncts reported short-term 0.37 mm probing pocket depths (PPDs) reductions and 0.26 mm gains in clinical attachment levels (CALs) (*p* < 0.001) when compared with scaling and root planing (SRP) alone [26]. However, their clinical value remains inconclusive, as statistically substantial improvement does not often translate into clinical relevance [27].

The application of network meta-analysis (NMA) in a systematic review enables the comparison of multiple treatments of interest in the absence of head-to-head trials, and the results are expressed as estimates [28]. The NMA conducted by John et al. [25] demonstrated that their findings on the use of various adjuncts to SRP correspond to the American Dental Association (ADA)’s Clinical Practice Guideline published in 2015 [29]. They focused on CALs as the primary outcome and excluded products and medical devices that were not available in the United States [24].

Hence, the present systematic review and NMA aimed to present updated comprehensive information on the clinical efficacy of current commercially available local drug delivery and adjunctive agents (LDAs) on the global market used with conventional mechanical debridement to enable clinicians to exercise evidence-based decision making in the nonsurgical management of periodontitis.

## 2. Materials and Methods

The systematic review and NMA were conducted according to the Cochrane Handbook for Intervention Reviews [30] and Preferred Reporting Items for Systematic Reviews and Meta-Analyses (PRISMA) statement [31]. The review protocol was registered in the International Prospective Register of Systematic Reviews database (Registration number CRD42020137115) and received approval from The National University of Malaysia (UKM) Research Ethics Committee (Reference number UKM PPI/111/8/JEP-2019-042).

### 2.1. Inclusion and Exclusion Criteria

The studies included in the present review were human randomized controlled trials (RCTs) with split-mouth or parallel arm design with a minimum follow-up of 6 months. The participants included were systemically healthy, aged 18 years and above, and diagnosed with periodontitis. The diagnoses of chronic and aggressive periodontitis that were included as current evidence do not support the distinction between the two disease entities [32].

The intervention included subgingival mechanical debridement (SMD) with the use of LDAs. The LDAs must be commercially available, have a primary pharmacological, immunological, or metabolic mode of action within the periodontal pocket, and be administered as an adjunct to SMD. Experimental, discontinued, and/or banned LDAs were excluded.

Comparison groups included SMD alone or SMD with placebo. “SRP” and “ultrasonic scaling” were included into the definition of SMD as both techniques remove calculi, which serve as a nidus for plaque accumulation and impede the effectiveness of the LDA [33].

The primary outcomes of the present review were PPDs and CALs. The secondary outcome measures were bleeding on probing (BOP), post-operative adverse effects associated with LDAs and patient-related outcome measures (PROMs).

### 2.2. Information Sources and Search

Five electronic databases, namely, Cochrane Central Register of Controlled Trials, MEDLINE, PubMed, EMBASE and Web of Science, were searched (File S1). Gray literature search included ProQuest Dissertations and Theses Global, conference abstracts, the World Health Organization (WHO) International Clinical Trials Registry Platform and ClinicalTrials.gov. A hand search was carried out to identify potential papers from the last 5 years from relevant journals, and the bibliographies of all eligible papers and review articles were examined for relevant studies that might have been missed in the electronic search. All searches were conducted by two independent reviewers (O.L.T. and M.R.) up to 31 January 2020 with no restriction in publication status or language.

### 2.3. Study Selection and Data Extraction

The same reviewers (O.L.T. and M.R.) independently screened all titles/abstracts and assessed the eligibility of each full-text article. A third reviewer (S.H.S.) was consulted if disagreements were not settled by discussion. The details recorded were general study characteristics, study design, characteristics of participants, disease severity, treatment and outcome measures, and adverse events. The effect size measure for the continuous outcomes was weighted mean difference (MD) and its 95% confidence intervals (CIs).

Weights were adjusted for multi-arm and split-mouth studies using the reducing weights approach to eliminate bias resulting from the dependency of treatment arms and yield a more reliable and accurate NMA result [34,35]. The risk of bias of the included studies was evaluated independently by the two reviewers (O.L.T. and M.R.) using the Cochrane Collaboration’s Risk of Bias tool (RoB 2.0) [36].

### 2.4. Statistical Analyses

NMA with frequentist model was conducted using the netmeta package [37] in the statistical program R (version 3.6.2; R Foundation for Statistical Computing) to directly and indirectly compare the efficacy of each treatment of interest. A random-effects model was used on the assumption of the presence of heterogeneous effects across the network. Network plots were produced where the size of the node (circle) represents the number of participants involved, and the size of the connection (line width) represents the number of studies per treatment. The line of the network plot represents the direct comparison between the interventions. Summary MDs for all pairwise comparisons are presented in league tables. Treatments were ranked according to their P-scores, which were based on their point estimates’ and network estimates’ standard errors. A higher P-score represents a greater likelihood for the treatment to be ranked as best among the other competing treatments [38].

Global statistical heterogeneity was measured using I-squared (*I*^2^) statistics [39]. Inconsistency in the NMA was assessed through the comparison of direct and indirect evidence with net splitting. Comparison-adjusted funnel plots were generated to assess for any publication bias and small-study bias for all the possible comparisons of treatment versus SMD. Sensitivity analyses were conducted by excluding studies with parameters that may affect treatment effect and by re-running the NMA without each parameter.

Subgroup analyses were performed for the possible treatment effect of influencing factors. The threshold for statistical significance was set at a MD of 0. The clinically meaningful threshold (CMT) for clinical significance was set at 1 mm for PPD reduction and 0.5 mm for CAL gain. Clinical significance depends on the relationship of the CMT of the intervention to its average MD and surrounding 95% CI and was reported according to the criteria defined by Man-Son-Hing et al. [40]

### 2.5. Certainty of Evidence

Certainty of evidence was assessed using the Grading of Recommendations Assessment, Development and Evaluation (GRADE) approach [41]. For direct comparisons, RCTs initially have a high certainty of evidence, which will be reduced according to the seriousness in the risk of bias, imprecision, indirectness, inconsistency, and publication bias. Ratings for direct and indirect estimates contributed toward the network estimate, which includes local imprecision and incoherence [41,42]. A modified “summary of findings” table [43] was created for an overview of the NMA results with interpretation of the findings.

## 3. Results

**Figure 1:**
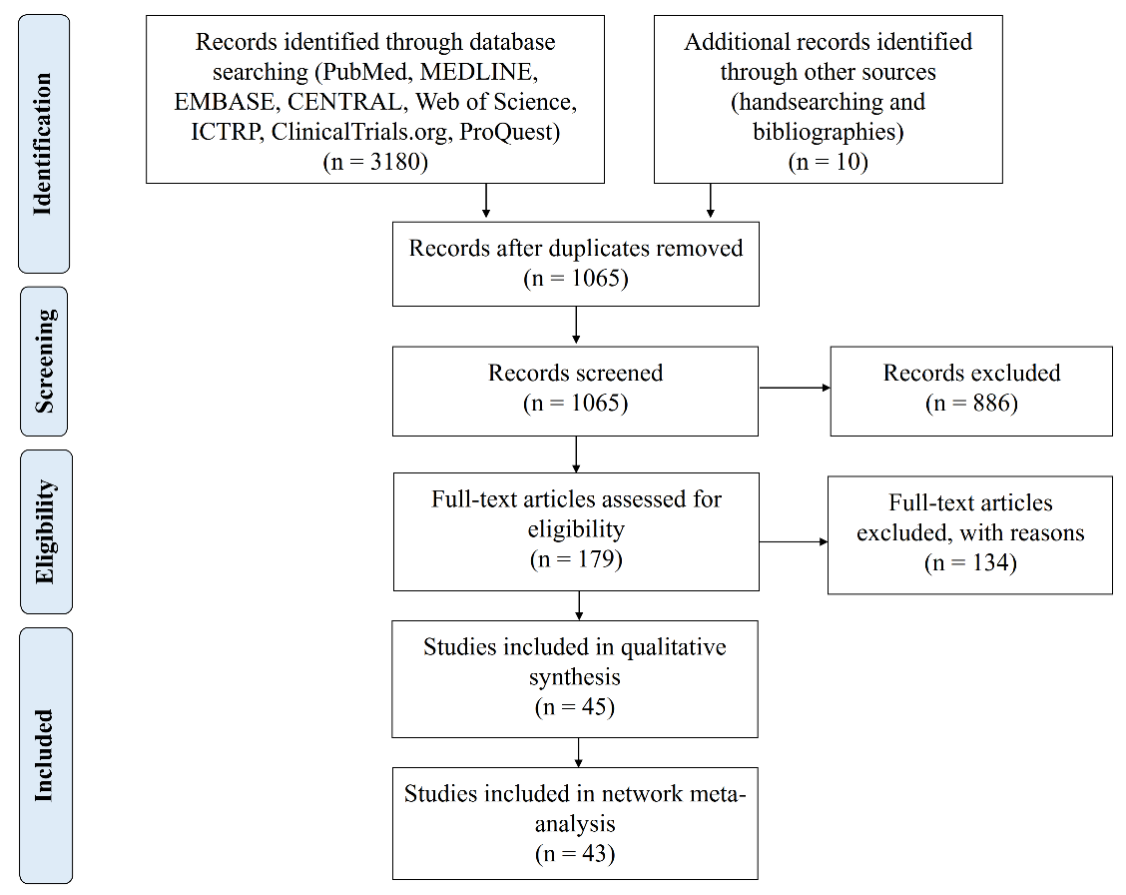
Preferred Reporting Items for Systematic Reviews and Meta-Analyses (PRISMA) flow diagram of the study selection process.

A total of 1065 studies were identified in the initial screening, and 179 studies were selected for full-text review after title and abstract screening. Forty-five studies satisfied the inclusion criteria, and 43 studies were considered eligible for the NMA (Figure 1 and Table S1). Cohen’s kappa for interrater reliability of the “title and Abstract screening” and “full-text screening” were 0.78 (95% CI 0.61 to 0.95) and 0.78 (95% CI 0.69 to 0.87), both indicating moderate agreement [44].

### 3.1. Characteristics of Included Studies

The 45 studies selected for qualitative synthesis consisted of 20 parallel and 25 split-mouth design trials (Tables S2–S5). Two studies [45,46] were associated with two selected trials [47,48] and were not included in the quantitative analysis.

The studies were published between 2000 and 2019 and were conducted in 19 countries. All selected studies were reported in English except that of Zhao et al. [49], which was written in Chinese. Fourteen of the included studies were commercially supported. The United States Food and Drug Administration (FDA) report [48] was split into two studies (103A and 103B) as the two study centers have their own individual control groups with supplement data from Williams et al. [46] Likewise, Agan et al. [50] had two subgroups based on the disease definition of chronic and aggressive periodontitis. Therefore, a total of 45 studies were included in the present review.

### 3.2. Risk of Bias

**Figure 2:**
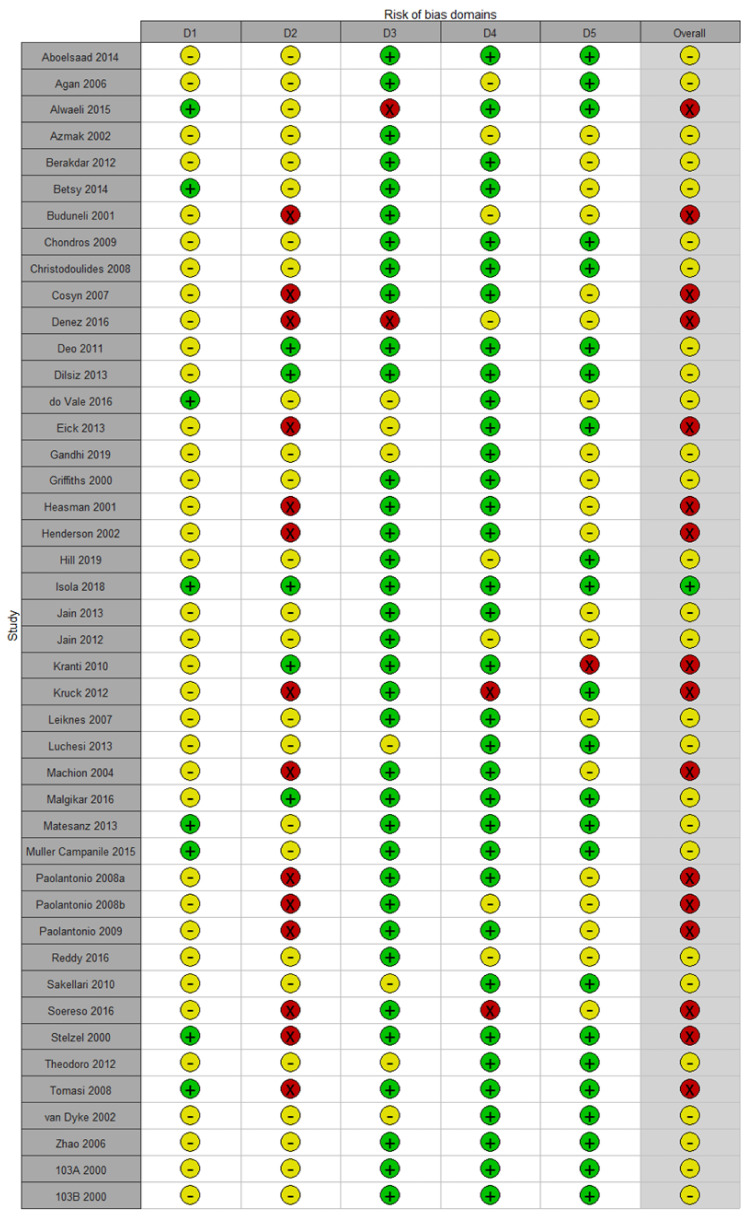

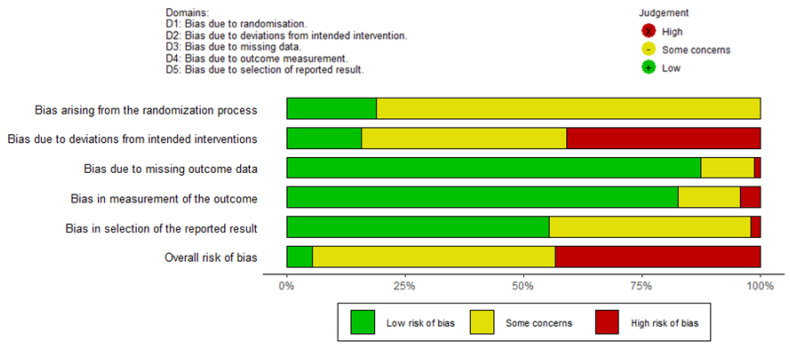
Risk of bias summary of the included trials.

Only one study [51] had a low risk of bias. Important information needed to assess the risk of bias was often not reported or incomplete. The most serious issue in the methodology was the risk of bias because of deviations from the intended interventions (effect of assignment to intervention), as the affected studies were single-blinded and utilized per-protocol instead of intention-to-treat data analysis. Sixteen studies had a high risk of bias. Overall, the majority of the studies (62.2%) demonstrated some concerns in their risk of bias (Figure 2).

### 3.3. Network Meta-Analyses

Evidence of significant heterogeneity and inconsistency (*p* < 0.0001) was observed upon the employment of the full NMA of all 45 studies. Hence, sensitivity analyses were conducted to examine the effects by removing trials based on possible influencing design parameters (File S2 and Table S6). The model divided by study design (split-mouth and parallel groups) and excluding high risk of bias trials had the most impact on the results and had low heterogeneity and inconsistency. Therefore, the NMA results are reported based on 29 studies and 14 types of adjuncts after the removal of studies with high risk of bias.

The 29 RCTs consisted of 16 split-mouth and 13 parallel groups and involved 2059 participants. Three studies were multi-arm trials [48,52,53]. The adjuncts included were: (1) antimicrobial photodynamic therapy (aPDT) indocyanine green (ICG) [54,55], (2) aPDT methylene blue (MB) 0.005% [56,57], (3) aPDT MB 1% [47,58,59,60], (4) aPDT phenothiazine chloride (PC) [61,62], (5) aPDT toluidine blue O (TBO) [53], (6) chlorhexidine (CHX) chip [52,63,64], (7) CHX xanthan gel [65,66], (8) doxycycline hyclate (DH) gel [50,67], (9) metronidazole (MET) gel [68,69], (10) minocycline (MINO) gel [49,70], (11) MINO microspheres [48,71,72], (12) povidone iodine (PI) 10% irrigating solution [73], (13) sulfonic/sulfuric acid (SA) gel [51], and (14) tetracycline (TC) fibers [52]. SMD alone was used as the reference comparator arm. Only Isola et al. [51] and Reddy et al. [52] performed 12-month follow-up, whereas the other studies had either 6-month or 9-month follow-up.

#### 3.3.1. PPD Changes

**Figure 3:**
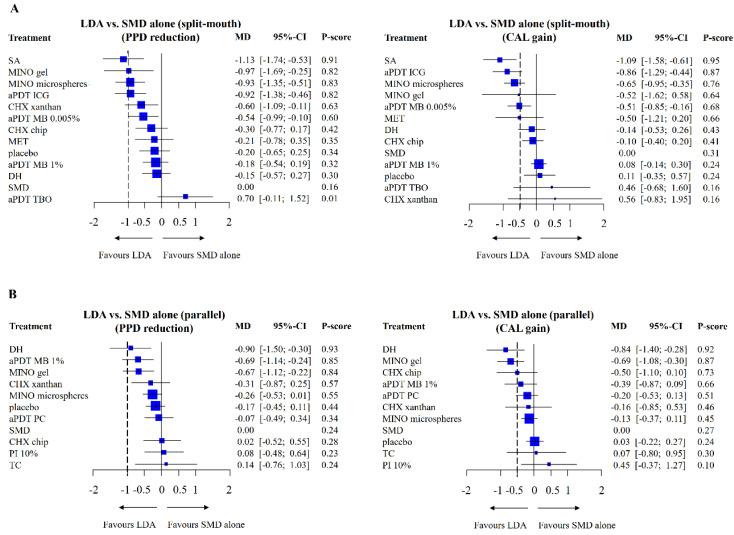
(**A**) Forest plot showing probing pocket depth (PPD) reduction and clinical attachment level (CAL) gain in local drug delivery and adjunctive agents (LDAs) vs. subgingival mechanical debridement (SMD) alone in split-mouth studies. (**B**) Forest plot showing PPD reduction and CAL gain in LDAs vs. SMD alone in parallel studies. The P-score indicates the treatment’s probability in ranking the best in efficacy in the network. The dotted line represents the threshold for clinical significance.

SA gel had probable clinical significance, whereas MINO gel, MINO microspheres, aPDT ICG, and CHX xanthan were possibly significant clinically in split-mouth studies (Figure 3A). The other LDAs had possible clinical significance without statistical significance with the exception of aPDT TBO, which fared worse than SMD. SA gel ranked first relative to SMD with a P-score of 0.91, which indicates a 91% probability of being ranked first when all of the treatments (with control groups included) were compared with each other. No important heterogeneity (*I*^2^ = 23.3%) was seen between the studies when the data were pooled.

**Figure 4:**
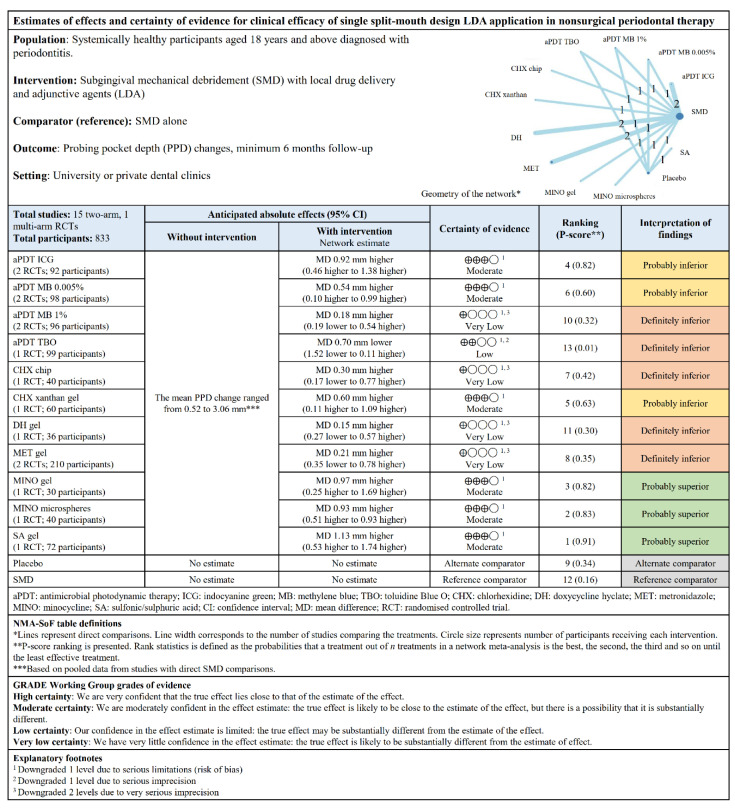
“Summary of findings” table showing the estimates of effects and certainty of evidence for PPD changes for single split-mouth design LDA application.

**Figure 5:**
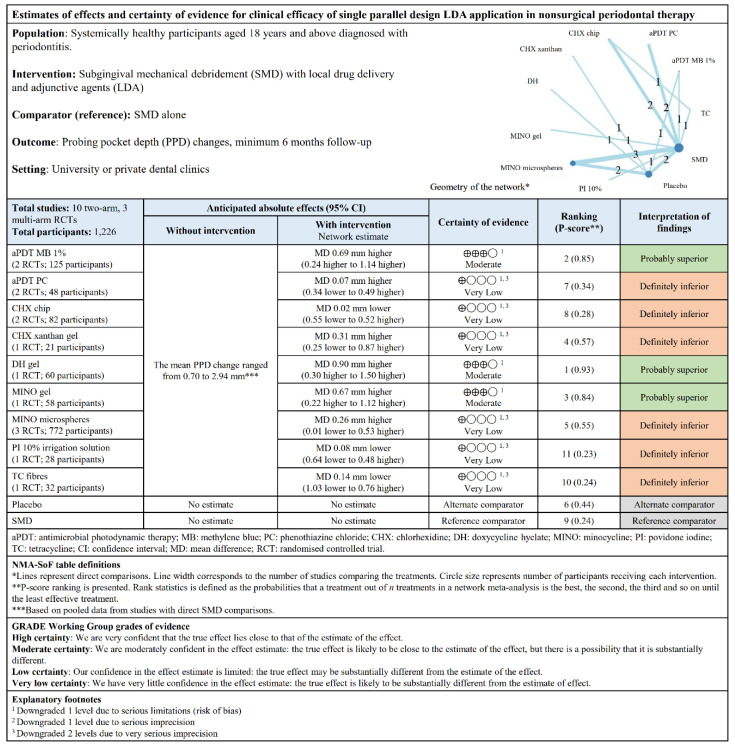
“Summary of findings” table showing the estimates of effects and certainty of evidence for PPD changes for single parallel design LDA application.

For parallel design studies, DH gel, aPDT MB 1%, and MINO gel were possibly significant clinically (Figure 3B). The other LDAs had no significance, especially CHX chip, PI 10%, and TC, which fared worse than SMD. DH gel ranked first relative to SMD with a P-score of 0.93. Low heterogeneity (*I*^2^ = 47.7%) was observed. The “summary of findings” tables (Figure 4 and Figure 5) are based on the GRADE certainty of evidence (Table S8).

#### 3.3.2. CAL Changes

SA gel had definite clinical significance, whereas aPDT ICG, MINO microspheres, and aPDT MB 0.005% were probably significant clinically in split-mouth studies (Figure 3A). Other LDAs had probable or possible clinical significance without statistical significance with the exception of CHX chip (not significant), aPDT MB 1%, aPDT TBO, and CHX xanthan gel, which fared worse than SMD. SA gel ranked first relative to SMD with a P-score of 0.95. No important heterogeneity was seen between the studies (*I*^2^ = 0%) when the data were pooled.

The findings from the parallel design studies demonstrated that DH gel and MINO gel were probably clinically significant (Figure 3B). The other LDAs had possible clinical significance without statistical significance except for MINO microspheres (not significant clinically and statistically), TC and PI 10% (fared worse than SMD). DH gel ranked first relative to SMD with a P-score of 0.92. Low heterogeneity was seen between the studies (*I*^2^ = 24.8%).

**Figure 6:**
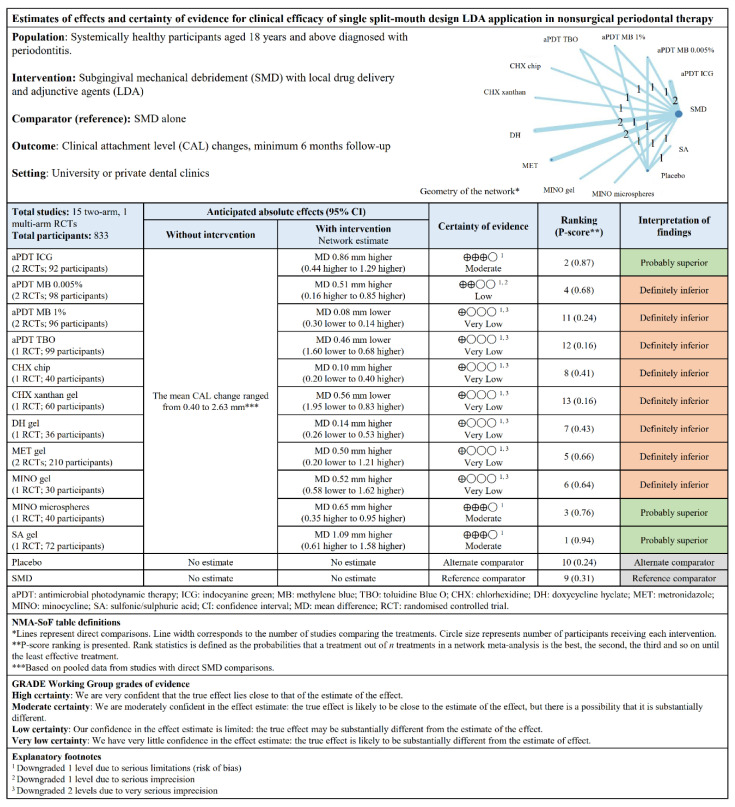
“Summary of findings” table showing the estimates of effects and certainty of evidence for CAL changes for single split-mouth design LDA application.

**Figure 7:**
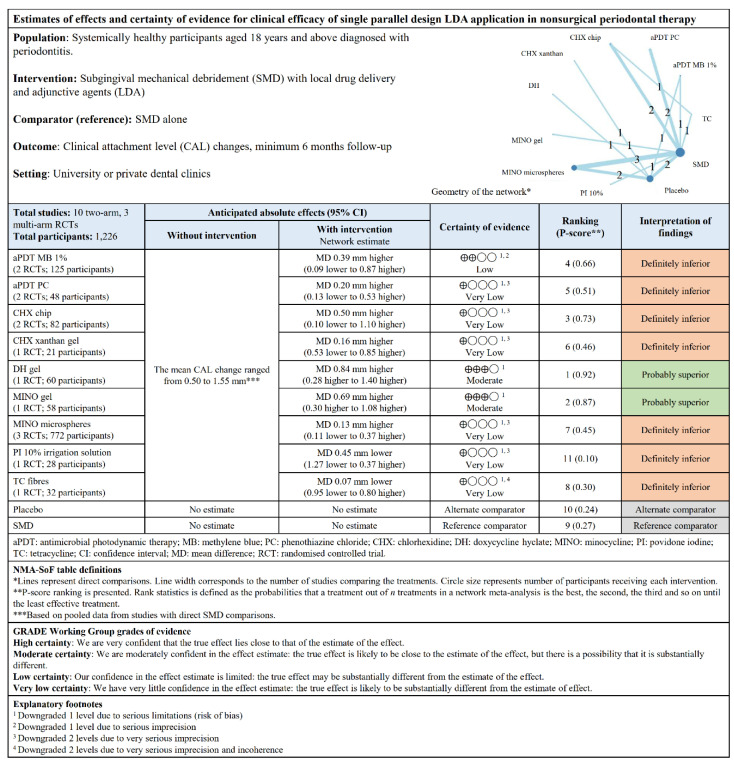
“Summary of findings” table showing the estimates of effects and certainty of evidence for CAL changes for single parallel design LDA application.

The direct and indirect comparisons of the treatments in the NMA are presented in the league tables (Figure S1). No inconsistency was seen overall (Figure S2). The “summary of findings” tables (Figure 6 and Figure 7) were based on the GRADE certainty of evidence (Table S8). Overall, no publication bias and small-study bias were observed in both study designs (Figure S3).

### 3.4. Subgroup Analyses

Subgroup analyses were conducted according to study duration, smoking status, treatment phase, treatment area, debridement method, type of control group, and funding source (Table S9). A significant subgroup effect was detected for study duration in PPD (*p* = 0.002) and CAL (*p* < 0.0001) outcomes in the split-mouth design and only CAL (*p* = 0.04) outcome for the parallel design studies. Smoking status had a significant subgroup effect in CAL (*p* = 0.01) outcomes in the split-mouth design studies. The funding source had a significant impact on PPD (*p* = 0.03) and CAL (*p* = 0.02) outcomes in the parallel design studies.

### 3.5. Secondary Outcomes

Thirty-four studies (75.6%) reported changes in BOP; however, eight different gingival bleeding indices were used (Table S3). PROMs in relation to adverse events were evaluated in more than half of the studies (55.5%), and 21 studies (84%) reported no adverse events post-intervention.

This section may be divided by subheadings. It should provide a concise and precise description of the experimental results, their interpretation, as well as the experimental conclusions that can be drawn.

## 4. Discussion

### 4.1. Summary of Findings

NMA was conducted to evaluate the clinical efficacy of local adjuncts used in nonsurgical periodontal therapy (NSPT) against a common comparator of mechanical debridement alone. Our findings suggest that based on a single LDA application, SA gel (split-mouth design) and DH gel (parallel design) were the most effective in PPD reduction and CAL gain with a probable superiority over SMD alone (moderate certainty). Although SA gel and DH gel were highly ranked in clinical efficacy for both outcomes, these adjuncts had fewer participants when compared with the studies of other adjuncts in this review.

In this review, an average reduction of 0.3–0.48 mm in PPD (ranged between −0.7 and 1.13 mm) and 0.27–0.3 mm gain in CAL (ranged between −0.56 and 1.09 mm) were seen compared with SMD alone. In general, the use of local adjuncts seems to have a lack of clinical significance. However, study-level data have shown that SA gel exhibited a more considerable clinical significance for both primary outcomes compared with other adjuncts.

The certainty of evidence ranged between very low and moderate for the outcomes. Although the study on SA gel was initially judged as having high certainty, a decision was made to downgrade the confidence, as it was the only study with a small sample size utilizing the adjunct. The adjuncts with moderate certainty for PPD reduction were MINO gel (both designs), MINO microspheres (split-mouth design), and aPDT MB 1% (parallel design). aPDT ICG and MINO microspheres (split-mouth design), as well as MINO gel (parallel design), showed moderate certainty for CAL gain. The other studies were predominantly downgraded because of issues in study limitations and imprecision. Eight studies (17.8%) reported minor to moderate adverse events [74] across the different adjuncts; thus, LDAs are relatively safe for use in clinical practice.

The test for subgroup differences suggested the presence of a statistically significant subgroup effect in study duration, smoking, and industrial funding. However, the numbers of participants in the test and control groups were imbalanced. A considerably smaller number of studies and participants contributed data to the split-mouth (one long-duration study with 36 participants vs. 15 medium-duration studies with 388 participants) and parallel (one long-duration study with 32 participants vs. 12 medium-duration studies with 913 participants) duration subgroup. Likewise, the number of participants in the nonsmoker subgroup was double the participants in the smoker subgroup for split-mouth design (three studies with 135 participants vs. 13 studies with 289 participants). Similarly, the number of participants were not equal with commercially funded subgroup outnumbering noncommercial funding subgroup by two times for parallel design. Therefore, the subgroup analyses are unlikely to produce a useful valid conclusion [75]. Hence, a general conclusion regarding the effect of study duration, smoking, and industrial funding on treatment effects could not be reached.

Based on the definition of periodontitis severity reported in the included studies, the case definitions would coincide with the latest classification of localized and generalized Stage II or III and Grade B or C (for aggressive cases or those modified by smoking) periodontitis [32]. However, the proper reclassification of periodontal disease used in the studies is difficult because of the lack of information provided.

### 4.2. Comparison to Other Reviews

Previous pairwise systematic reviews and meta-analyses [21,22,23] had reported an average mean PPD reduction of 0.06–0.70 mm and average mean CAL gain of −0.04–0.46 mm based on different local antimicrobials. The authors of the ADA systematic review reported an average CAL improvement of 0.24–0.64 mm [24] with assorted local adjunct use. Furthermore, a 0.32 mm average was reported when all local and systemic adjuncts were combined together compared with SMD alone [25]. The latest systematic review [26] concluded that local antimicrobials adjunctive 6-month to 9-month studies had a mean reduction of 0.37 mm in PPD and a mean gain of 0.26 mm in CAL. Long-term studies had a mean reduction of 0.19 mm in PPD and a mean gain of 0.09 mm in CAL, albeit with substantial heterogeneity noted in nearly all of their analyses. A summary of the previous reviews can be seen in Table 1.

The ADA Clinical Practice Guidelines [29] were “expert opinion for” DH gel and MINO microspheres adjunctive use as evidence was weak with a low level of certainty. The recommendation for CHX chips and aPDT adjuncts was “weak” albeit with moderate level of certainty, which the authors suggested should be implemented only after other alternatives have been considered. The adjuncts recommended by their study slightly differ, as they had excluded products that are not available in the United States and other medical devices. Furthermore, this study’s certainty of evidence was based on the GRADE assessment for NMA [41,42,43]; therefore, the criteria might be different from their expert panel’s direction of recommendations.

The present study would be the second NMA conducted after that of John et al. [25] The authors had placed DH gel and aPDT with the greatest probabilities in being ranked as first and second, respectively, which was somewhat similar to the findings of our study. However, their network did not include any multi-arm trials. Likewise, Herrera et al. [26] reported the largest observed benefits from DH and MINO-based products.

This review had included SA gel which is a fairly new LDA that contains a concentrated blend of sulfonic or sulfuric acids that was traditionally used in the management of aphthous stomatitis [76]. These acids form a strong bond with water available in the biofilm matrix, which rapidly detaches, destroys, and eradicates the biofilm itself. A preliminary study that utilized this desiccant with ultrasonic mechanical debridement found greater bacterial load reduction and enhanced effectiveness of mechanical debridement [77].

PI 10% subgingival irrigation appears to have no clinical benefits in this study. A previous systematic review [23] also did not find any additional benefits of CHX irrigation compared with SRP alone. The authors attributed this finding to the rapid clearance by gingival crevicular fluid at site and the inability of the irrigation solution to reach adequate subgingival depth [78].

NMA was subdivided into split-mouth and parallel designs because a high inconsistency in network design was detected. The inclusion of both study designs could impart substantial heterogeneity in the meta-analysis as proven in the systematic review by Herrera et al. [26] Split-mouth design may introduce bias to the treatment effect estimates because of carry-across effects, in which a treatment effect is potentially leaked from one side to the other [79]. Therefore, the study designs were split to obtain a higher certainty of evidence. SA gel still came out as the most effective in PPD reduction and CAL gain among the LDAs when compared with the full network of 45 studies (Table S7). However, DH gel dropped in the ranks as SA gel was not present in the parallel design network.

Clinical significance was rarely discussed in the previous reviews as statistical significance is routinely used in clinical periodontal research. Statistically significant improvements are often reported in studies but do not often translate into clinical relevance [27,80]. Although the *p*-value is important, the inclusion of confidence intervals (CIs) can help in the clinician’s judgment for potential relevance in practice [81]. Clinical significance can be established by determining the minimum important difference or clinically meaningful threshold [81]. Smiley and his colleagues [29] developed a four-category clinical relevance scale for CAL gain as a part of their clinical recommendation summary. This study had chosen to use an objective method to determine clinical importance based on Man-Son-Hing et al. [40] with the clinically meaningful threshold set at 1 mm for PPD reduction and 0.5 mm for CAL gain. These threshold values were derived from previous recommendations [80,82] and the average improvement of PPD and CAL with NSPT alone [29,83]. However, these values are surrogate outcome measures [84] that do not truly reflect true clinical significance that should include long-term tooth survival, cost, time, risks, and complications [85]. Without a doubt, statistical analysis is necessary for clinical research, but future clinical periodontal research can be further improved with a cost–benefit analysis for results to be clinically applicable in our daily practice.

### 4.3. Strengths and Limitations

One of the strengths of this systematic review is the implementation of the latest NMA GRADE approach for the certainty of evidence [41,43]. NMA was chosen, as this statistical method enables the comparison of multiple treatments and the attainment of estimates in the absence of head-to-head trials. This review also made considerable effort to include all RCTs without any language restriction. We believe that this study is the first NMA to do so and also included a broader inclusion of local adjuncts not restricted by regions as compared with the previous NMA on the same topic [25]. The focus of this review on current commercially available LDAs with the exclusion of experimental adjuncts was to ensure that the adjuncts investigated were regulatory approved and could be readily obtained for use by clinicians.

The limitations of this review involve the small magnitude of the included studies and the lack of head-to-head trials of the adjuncts with conclusions derived from indirect comparisons of the treatments. Moreover, the majority of the studies presented some concerns in the risk of bias as a result of methodological flaws in blinding and per-protocol data analysis. Meta-analysis for BOP outcome was not conducted because of the high heterogeneity of the indices and measurements used. Although Herrera et al. [26] conducted BOP meta-analysis in their study, obvious substantial heterogeneity was detected.

## 5. Conclusions

Four NMAs suggested that SA gel and DH gel were the most effective in reducing PPD and gaining CAL in split-mouth and parallel studies, respectively. Both had probable superiority over SMD alone (moderate certainty of evidence) and SA gel having probable to definite clinical significance in the primary outcome measures. The other LDAs that were probably superior compared with SMD alone and had possible clinical importance when used as adjuncts in NSPT were MINO gel, MINO microspheres, aPDT MB 1%, and aPDT ICG (moderate level of certainty). In addition to the clinical efficacy of LDAs, clinicians would need to further assess the profile of a patient, the availability of adjunct, and the cost–benefit ratio when making an informed clinical decision to utilize the adjunct for NSPT.

## Figures and Tables

**Table 1:** Summary table of systematic reviews with meta-analyses comparing clinical efficacy of different LDAs for nonsurgical periodontal therapy (NSPT).

Authors and Year	Study Duration	Number of Studies	LDAs Studied	Main Outcomes and Conclusion
Hanes and Purvis 2003 [23]	≥3 months	28 RCTs, 2 CCTs, 2 cohort	Local antimicrobials	1. Sample-size adjusted mean PPD reduction 1.45 mm (*p* = 0.002; 95% CI 0.56 to 2.34), and adjusted mean CAL gain 0.89 mm (*p* = 0.001; 95% CI 0.55 to 1.24) was seen from SRP alone. 2. A WMD range of 0.06–0.51 mm PPD reduction and −0.40–0.39 mm CAL gain was observed with different sustained-released adjuncts. 3. MINO gel and microencapsulated MINO had shown significant PPD reductions while CHX chip and DOXY gel had significant CAL gains. 4. No additional benefits from local CHX irrigation studies. 5. Adverse events were infrequent and minimal.
Bonito et al., 2005 [22]	Not specified	50 RCTs	Local antimicrobials	1. A range of 0.24–0.49 mm mean PPD reduction and 0.12–0.46 mm mean CAL gain can be seen with adjunctive local antibiotics. 2. Combination of PPD and CAL results suggested local MINO to be the most promising adjunct (PPD 0.49 mm, CAL 0.46 mm), followed by local TET. 3. Adverse events reported were relatively minor.
Matesanz-Pérez et al., 2013 [21]	Not specified	52 RCTs	Local antimicrobials	1. Overall effect was statistically significant (*p* = 0.000) for both changes in PPD (WMD 0.407 mm) and CAL (WMD 0.310 mm). 2. No significant differences detected for bleeding on probing and plaque index. 3. Subgingival application of TET fibers, sustained released DOXY and MINO had substantial advantages in PPD reduction (WMD between 0.5 and 0.7 mm).
Smiley et al., 2015 [29]	≥6 months	72 RCTs	Local and systemic adjuncts only available in the United States	1. Approximately 0.5 mm average improvement in CAL can be seen with SRP alone (moderate level of certainty). 2. A range of 0.2–0.6 mm average CAL improvements can be seen in the combinations of assorted adjuncts over SRP alone. 3. Four adjunctive therapies were beneficial compared with SRP alone (moderate level of certainty): SDD, systemic antimicrobials, CHX chips and photodynamic therapy with a diode laser.
John et al., 2017 [25] *		61 RCTs		1. NMA found DOXY hyclate (WMD 0.64 mm, 95% CI 0.02 to 1.26) and PDT with diode laser (WMD 0.55 mm, 95% CI 0.25 to 0.85) to have the highest probabilities for ranking first and second adjuncts in terms of CAL gain, respectively. 2. 0.32 mm (95% CI 0.24 to 0.40) CAL improvement seen with adjuncts over 6–12 months with no significant differences among them. 3. Publication bias was detected, and the lack of studies inflated estimated 20% of treatment effects.
Herrera et al., 2020 [26]	≥6 months	50 RCTs	Local antimicrobials	1. Medium-duration studies (6–9 months) had statistically significant differences for PPD (WMD 0.365 mm, 95% CI 0.262 to 0.468) and CAL (WMD 0.263 mm, 95% CI 0.123 to 0.403). 2. Long-duration studies (≥12 months) had statistically significant difference for PPD (WMD 0.190 mm, 95% CI 0.059 to 0.321). 3. No adverse events observed.
This study	≥6 months	45 RCTs	Commercially available LDAs	1. SA gel (PPD MD −1.13 mm, 95% CI −1.74 to −0.53, P-score 0.91; CAL MD −1.09 mm, 95% CI −1.58 to −0.61, P-score 0.95) and DOXY hyclate gel (PPD MD −0.90 mm, 95% CI −1.50 to −0.30, P-score 0.93; CAL MD −0.84 mm, 95% CI −1.40 to −0.28, P-score 0.92) were the most effective in reducing PPD and gaining CAL in split-mouth and parallel studies, respectively (moderate certainty of evidence). 2. SA gel has probable to definite clinical significance in the primary outcome measures. 3. The other LDAs that were probably superior compared with SMD alone and had possible clinical importance when used as adjuncts in NSPT were MINO gel, MINO microspheres, antimicrobial PDT MB 1% and antimicrobial PDT ICG (moderate level of certainty). 4. Eight studies (17.8%) reported minor to moderate adverse events across the different adjuncts; thus, LDAs are relatively safe for use in clinical practice.

RCT: randomized controlled trial; CCT: case-controlled trial; n/a: not available; SRP: scaling and root planning; PPD: probing pocket depth; CAL: clinical attachment level; WMD: weighted mean difference (historical term); MD: mean difference; CHX: chlorhexidine; MINO: minocycline; DOXY: doxycycline; MET: metronidazole; TET: tetracycline; SDD: sub-antimicrobial-dose doxycycline; PDT: photodynamic therapy; * Network analysis of systematic review by Smiley et al. [29].

## Supplementary Materials

The following are available online at https://www.mdpi.com/1999-4923/12/11/1086/s1, File S1: Search strategies for each electronic database, Table S1: Studies excluded after full-text screening and reasons for exclusion, Table S2: Study characteristics of included studies, Table S3: Clinical outcome assessment of included studies, Table S4: Intervention characteristics of included studies, Table S5: Outcomes data of included studies, File S2: Sensitivity analyses, Table S6: Sensitivity analyses of specific parameters, Table S7: Comparison of PPD and CAL mean differences (95% CI), P-score and ranks from complete analysis and having removed studies at high ROB, File S3: Sensitivity analyses of studies with imputed data, Figure S1: League tables, Figure S2: Inconsistency plot for the network meta-analysis, Figure S3: Comparison-adjusted funnel plot for publication and small-study bias, Table S8: GRADE certainty of evidence for primary outcomes.

## References

[1] Vos T., Lim S.S., Abbafati C., Abbas K.M., Abbasi M., Abbasifard M., Abbasi-Kangevari M., Abbastabar H., Abd-Allah F., Abdelalim A. (2020). Global burden of 369 diseases and injuries in 204 countries and territories, 1990–2019: A systematic analysis for the Global Burden of Disease Study 2019. Lancet.

[2] Mohd-Dom T.N., Mohd-Said S., Abdul-Manaf M.R., Abdul-Muttalib K., Aljunid S.M. (2016). A detailed framework of methods used to calculate costs of periodontal treatment in Malaysian public dental clinics. Malays. J. Public Health Med..

[3] Mohd Dom T.N., Ayob R., Abd Muttalib K., Aljunid S.M. (2016). National economic burden associated with management of periodontitis in Malaysia. Int. J. Dent..

[4] Janakiram C., Dye B.A. (2020). A public health approach for prevention of periodontal disease. Periodontology 2000.

[5] Saeedi P., Petersohn I., Salpea P., Malanda B., Karuranga S., Unwin N., Colagiuri S., Guariguata L., Motala A.A., Ogurtsova K. (2019). Global and regional diabetes prevalence estimates for 2019 and projections for 2030 and 2045: Results from the International Diabetes Federation Diabetes Atlas, 9th edition. Diabetes Res. Clin. Pract..

[6] World Health Organization (2019). WHO Global Report on Trends in Prevalence of Tobacco Use 2000–2025.

[7] Ganesan S.M., Joshi V., Fellows M., Dabdoub S.M., Nagaraja H.N., O’Donnell B., Deshpande N.R., Kumar P.S. (2017). A tale of two risks: Smoking, diabetes and the subgingival microbiome. ISME J..

[8] Ferreira M.C., Dias-Pereira A.C., Branco-de-Almeida L.S., Martins C.C., Paiva S.M. (2017). Impact of periodontal disease on quality of life: A systematic review. J. Periodontal Res..

[9] Petsos H., Schacher B., Ramich T., Nickles K., Dannewitz B., Arendt S., Seidel K., Eickholz P. (2020). Retrospectively analysed tooth loss in periodontally compromised patients: Long-term results 10 years after active periodontal therapy—Patient-related outcomes. J. Periodontal Res..

[10] Lang N.P., Salvi G.E., Sculean A. (2019). Nonsurgical therapy for teeth and implants—When and why?. Periodontology 2000.

[11] Herrera D., Matesanz P., Bascones-Martínez A., Sanz M. (2012). Local and systemic antimicrobial therapy in periodontics. J. Evid. Based. Dent. Pract..

[12] Greenstein G., Polson A. (1998). The role of local drug delivery in the management of periodontal diseases: A comprehensive review. J. Periodontol..

[13] Ho S.N., Acharya A., Sidharthan S., Li K.Y., Leung W.K., McGrath C., Pelekos G. (2020). A systematic review and meta-analysis of clinical, immunological, and microbiological shift in periodontitis after nonsurgical periodontal therapy with adjunctive use of probiotics. J. Evid. Based. Dent. Pract..

[14] Moro M.G., Silveira Souto M.L., Franco G.C.N., Holzhausen M., Pannuti C.M. (2018). Efficacy of local phytotherapy in the nonsurgical treatment of periodontal disease: A systematic review. J. Periodontal Res..

[15] Azizan N., Mohd-Said S., Mazlan M.K.F., Chelvan K.T., Hanafiah R.M., Zainal-Abidin Z. (2019). In-vitro inhibitory effect of Cinnamomum zeylanicum and Eugenia caryophyllata oils on multispecies anaerobic oral biofilm. J. Int. Dent. Med. Res..

[16] Azizan N., Mohd Said S., Zainal Abidin Z., Jantan I. (2017). Composition and antibacterial activity of the essential oils of Orthosiphon stamineus Benth and Ficus deltoidea Jack against pathogenic oral bacteria. Molecules.

[17] Hajishengallis G., Chavakis T., Lambris J.D. (2020). Current understanding of periodontal disease pathogenesis and targets for host-modulation therapy. Periodontology 2000.

[18] 360 Market Updates Global Periodontal Therapeutics Market Size, Status and Forecast 2020–2026. https://www.360marketupdates.com/global-periodontal-therapeutics-market-15840303.

[19] Tan O.L., Safii S.H., Razali M. (2020). Commercial local pharmacotherapeutics and adjunctive agents for nonsurgical treatment of periodontitis: A contemporary review of clinical efficacies and challenges. Antibiotics.

[20] Rams T.E., Slots J. (1996). Local delivery of antimicrobial agents in the periodontal pocket. Periodontology 2000.

[21] Matesanz-Pérez P., García-Gargallo M., Figuero E., Bascones-Martínez A., Sanz M., Herrera D. (2013). A systematic review on the effects of local antimicrobials as adjuncts to subgingival debridement, compared with subgingival debridement alone, in the treatment of chronic periodontitis. J. Clin. Periodontol..

[22] Bonito A., Lux L., Lohr K. (2005). Impact of local adjuncts to scaling and root planing in periodontal disease therapy: A systematic review. J. Periodontol..

[23] Hanes P.J., Purvis J.P. (2003). Local anti-infective therapy: Pharmacological agents. A systematic review. Ann. Periodontol..

[24] Smiley C.J., Tracy S.L., Abt E., Michalowicz B.S., John M.T., Gunsolley J., Cobb C.M., Rossmann J., Harrel S.K., Forrest J.L. (2015). Systematic review and meta-analysis on the nonsurgical treatment of chronic periodontitis by means of scaling and root planing with or without adjuncts. J. Am. Dent. Assoc..

[25] John M.T., Michalowicz B.S., Kotsakis G.A., Chu H. (2017). Network meta-analysis of studies included in the Clinical Practice Guideline on the nonsurgical treatment of chronic periodontitis. J. Clin. Periodontol..

[26] Herrera D., Matesanz P., Martín C., Oud V., Feres M., Teughels W. (2020). Adjunctive effect of locally delivered antimicrobials in periodontitis therapy. A systematic review and meta-analysis. J. Clin. Periodontol..

[27] Chambrone L., Armitage G.C. (2016). Statistical significance versus clinical relevance in periodontal research: Implications for clinical practice. J. Periodontol..

[28] Salanti G. (2012). Indirect and mixed-treatment comparison, network, or multiple-treatments meta-analysis: Many names, many benefits, many concerns for the next generation evidence synthesis tool. Res. Synth. Methods.

[29] Smiley C.J., Tracy S.L., Abt E., Michalowicz B.S., John M.T., Gunsolley J., Cobb C.M., Rossmann J., Harrel S.K., Forrest J.L. (2015). Evidence-based clinical practice guideline on the nonsurgical treatment of chronic periodontitis by means of scaling and root planing with or without adjuncts. J. Am. Dent. Assoc..

[30] Higgins J.P.T., Thomas J., Chandler J., Cumpston M., Li T., Page M.J., Welch V.A. Cochrane Handbook for Systematic Reviews of Interventions version 6.0 (updated July 2019). www.training.cochrane.org/handbook.

[31] Hutton B., Salanti G., Caldwell D.M., Chaimani A., Schmid C.H., Cameron C., Ioannidis J.P.A., Straus S., Thorlund K., Jansen J.P. (2015). The PRISMA extension statement for reporting of systematic reviews incorporating network meta-analyses of health care interventions: Checklist and explanations. Ann. Intern. Med..

[32] Papapanou P.N., Sanz M., Buduneli N., Dietrich T., Feres M., Fine D.H., Flemmig T.F., Garcia R., Giannobile W.V., Graziani F. (2018). Periodontitis: Consensus report of workgroup 2 of the 2017 World Workshop on the Classification of Periodontal and Peri-Implant Diseases and Conditions. J. Periodontol..

[33] Hokari T., Morozumi T., Komatsu Y., Shimizu T., Yoshino T., Tanaka M., Tanaka Y., Nohno K., Kubota T., Yoshie H. (2018). Effects of antimicrobial photodynamic therapy and local administration of minocycline on clinical, microbiological, and inflammatory markers of periodontal pockets: A pilot study. Int. J. Dent..

[34] Su Y.-X., Tu Y.-K. (2018). Statistical approaches to adjusting weights for dependent arms in network meta-analysis. Res. Synth. Methods.

[35] Rücker G., Schwarzer G. (2014). Reduce dimension or reduce weights? Comparing two approaches to multi-arm studies in network meta-analysis. Stat. Med..

[36] Sterne J.A.C., Savović J., Page M.J., Elbers R.G., Blencowe N.S., Boutron I., Cates C.J., Cheng H.Y., Corbett M.S., Eldridge S.M. (2019). RoB 2: A revised tool for assessing risk of bias in randomised trials. BMJ.

[37] Rücker G., Krahn U., König J., Efthimiou O., Schwarzer G. (2020). Netmeta: Network Meta-Analysis Using Frequentist Methods.

[38] Rücker G., Schwarzer G. (2015). Ranking treatments in frequentist network meta-analysis works without resampling methods. BMC Med. Res. Methodol..

[39] Higgins J.P.T., Thompson S.G., Deeks J.J., Altman D.G. (2003). Measuring inconsistency in meta-analyses. Br. Med. J..

[40] Man-Son-Hing M., Laupacis A., O’Rourke K., Molnar F.J., Mahon J., Chan K.B.Y., Wells G. (2002). Determination of the clinical importance of study results: A review. J. Gen. Intern. Med..

[41] Brignardello-Petersen R., Bonner A., Alexander P.E., Siemieniuk R.A., Furukawa T.A., Rochwerg B., Hazlewood G.S., Alhazzani W., Mustafa R.A., Murad M.H. (2018). Advances in the GRADE approach to rate the certainty in estimates from a network meta-analysis. J. Clin. Epidemiol..

[42] Puhan M.A., Schünemann H.J., Murad M.H., Li T., Brignardello-Petersen R., Singh J.A., Kessels A.G., Guyatt G.H. (2014). A GRADE Working Group approach for rating the quality of treatment effect estimates from network meta-analysis. BMJ.

[43] Yepes-Nuñez J.J., Li S.A., Guyatt G., Jack S.M., Brozek J.L., Beyene J., Murad M.H., Rochwerg B., Mbuagbaw L., Zhang Y. (2019). Development of the summary of findings table for network meta-analysis. J. Clin. Epidemiol..

[44] McHugh M.L. (2012). Interrater reliability: The kappa statistic. Biochem. Med..

[45] Betsy J., Prasanth C.S., Baiju K.V., Presanthila J., Subhash N. (2016). Patients’ perceptions of antimicrobial photodynamic therapy in the management of chronic periodontitis. Photodiagnosis Photodyn. Ther..

[46] Williams R.C., Paquette D.W., Offenbacher S., Adams D.F., Armitage G.C., Bray K., Caton J., Cochran D.L., Drisko C.H., Fiorellini J.P. (2001). Treatment of periodontitis by local administration of minocycline microspheres: A controlled trial. J. Periodontol..

[47] Betsy J., Prasanth C.S., Baiju K.V., Prasanthila J., Subhash N. (2014). Efficacy of antimicrobial photodynamic therapy in the management of chronic periodontitis: A randomized controlled clinical trial. J. Clin. Periodontol..

[48] FDA Center for Drug Application Reviews Statistical Review and Evaluation, Application Number NDA 50-781. https://www.accessdata.fda.gov/drugsatfda_docs/nda/2001/50781_Arestin_statr.pdf.

[49] Zhao N., Ge S.H., Ding G.Y. (2006). Clinical effect of minocycline as adjunctive therapy to scaling and root planning on treatment of chronic periodontitis. Hua Xi Kou Qiang Yi Xue Za Zhi West China J. Stomatol..

[50] Ağan S., Sönmez S., Serdar M. (2006). The effect of topical doxycycline usage on gingival crevicular fluid MMP-8 levels of chronic and aggressive periodontitis patients: A pilot study. Int. J. Dent. Hyg..

[51] Isola G., Matarese G., Williams R.C., Siciliano V.I., Alibrandi A., Cordasco G., Ramaglia L. (2018). The effects of a desiccant agent in the treatment of chronic periodontitis: A randomized, controlled clinical trial. Clin. Oral Investig..

[52] Reddy S., Reddy S., Bhowmick N. (2016). A comparison of chlorhexidine and tetracycline local drug delivery systems in management of persistent periodontal pockets—A clinical study. Int. J. Appl. Dent. Sci..

[53] Theodoro L.H., Silva S.P., Pires J.R., Soares G.H.G., Pontes A.E.F., Zuza E.P., Spolidório D.M.P., de Toledo B.E.C., Garcia V.G. (2012). Clinical and microbiological effects of photodynamic therapy associated with nonsurgical periodontal treatment. A 6-month follow-up. Lasers Med. Sci..

[54] Gandhi K., Pavaskar R., Cappetta E., Drew H. (2019). Effectiveness of adjunctive use of low-level laser therapy and photodynamic therapy after scaling and root planing in patients with chronic periodontitis. Int. J. Periodontics Restor. Dent..

[55] Hill G., Dehn C., Hinze A.V., Frentzen M., Meister J. (2019). Indocyanine green-based adjunctive antimicrobial photodynamic therapy for treating chronic periodontitis: A randomized clinical trial. Photodiagn. Photodyn. Ther..

[56] Berakdar M., Callaway A., Eddin M.F., Roß A., Willershausen B. (2012). Comparison between scaling-root-planing (SRP) and SRP/photodynamic therapy: Six-month study. Head Face Med..

[57] Müller Campanile V.S., Giannopoulou C., Campanile G., Cancela J.A., Mombelli A. (2015). Single or repeated antimicrobial photodynamic therapy as adjunct to ultrasonic debridement in residual periodontal pockets: Clinical, microbiological, and local biological effects. Lasers Med. Sci..

[58] Dilsiz A., Canakci V., Aydin T. (2013). Clinical effects of potassium–titanyl–phosphate laser and photodynamic therapy on outcomes of treatment of chronic periodontitis: A randomized controlled clinical trial. J. Periodontol..

[59] Malgikar S., Reddy S.H., Sagar S.V., Satyanarayana D., Reddy G.V., Josephin J.J. (2016). Clinical effects of photodynamic and low-level laser therapies as an adjunct to scaling and root planing of chronic periodontitis: A split-mouth randomized controlled clinical trial. Indian J. Dent. Res..

[60] Luchesi V.H., Pimentel S.P., Kolbe M.F., Ribeiro F.V., Casarin R.C., Nociti F.H., Sallum E.A., Casati M.Z. (2013). Photodynamic therapy in the treatment of class II furcation: A randomized controlled clinical trial. J. Clin. Periodontol..

[61] Chondros P., Nikolidakis D., Christodoulides N., Rössler R., Gutknecht N., Sculean A. (2009). Photodynamic therapy as adjunct to non-surgical periodontal treatment in patients on periodontal maintenance: A randomized controlled clinical trial. Lasers Med. Sci..

[62] Christodoulides N., Nikolidakis D., Chondros P., Becker J., Schwarz F., Rössler R., Sculean A. (2008). Photodynamic therapy as an adjunct to non-surgical periodontal treatment: A randomized, controlled clinical trial. J. Periodontol..

[63] Azmak N., Atilla G., Luoto H., Sorsa T. (2002). The effect of subgingival controlled-release delivery of chlorhexidine chip on clinical parameters and matrix metalloproteinase-8 levels in gingival crevicular fluid. J. Periodontol..

[64] Sakellari D., Ioannidis I., Antoniadou M., Slini T., Konstantinidis A. (2010). Clinical and microbiological effects of adjunctive, locally delivered chlorhexidine on patients with chronic periodontitis. J. Int. Acad. Periodontol..

[65] Jain M., Dave D., Jain P., Manohar B., Yadav B., Shetty N. (2013). Efficacy of xanthan based chlorhexidine gel as an adjunct to scaling and root planing in treatment of the chronic periodontitis. J. Indian Soc. Periodontol..

[66] Matesanz P., Herrera D., Echeverría A., O’Connor A., González I., Sanz M., O’Connor A., González I., Sanz M., O’Connor A. (2013). A randomized clinical trial on the clinical and microbiological efficacy of a xanthan gel with chlorhexidine for subgingival use. Clin. Oral Investig..

[67] Deo V., Ansari S., Mandia S., Bhongade M. (2011). Therapeutic efficacy of subgingivally delivered doxycycline hyclate as an adjunct to non-surgical treatment of chronic periodontitis. J. Oral Maxillofac. Res..

[68] Griffiths G.S., Smart G.J., Bulman J.S., Weiss G., Shrowder J., Newman H.N. (2000). Comparison of clinical outcomes following treatment of chronic adult periodontitis with subgingival scaling or subgingival scaling plus metronidazole gel. J. Clin. Periodontol..

[69] Leiknes T., Leknes K.N., Böe O.E., Skavland R.J., Lie T. (2007). Topical use of a metronidazole gel in the treatment of sites with symptoms of recurring chronic inflammation. J. Periodontol..

[70] Jain R., Mohamed F., Hemalatha M. (2012). Minocycline containing local drug delivery system in the management of chronic periodontitis: A randomized controlled trial. J. Indian Soc. Periodontol..

[71] Van Dyke T.E., Offenbacher S., Braswell L., Lessem J. (2002). Enhancing the value of scaling and root-planing: Arestin clinical trial results. J. Int. Acad. Periodontol..

[72] Aboelsaad N., Ghandour R., Abiad R. (2014). Clinical efficacy of local delivered minocycline in the treatment of chronic periodontitis smoker patients. J. Dent. Oral Health.

[73] do Vale H.F., Casarin R.C.V., Taiete T., Bovi Ambrosano G.M., Ruiz K.G.S., Nociti F.H., Sallum E.A., Casati M.Z. (2016). Full-mouth ultrasonic debridement associated with povidone iodine rinsing in GAgP treatment: A randomised clinical trial. Clin. Oral Investig..

[74] U.S. Department of Health and Human Services (2017). Common Terminology Criteria for Adverse Events (CTCAE) Version 5.0.

[75] Richardson M., Garner P., Donegan S. (2019). Interpretation of subgroup analyses in systematic reviews: A tutorial. Clin. Epidemiol. Glob. Health.

[76] Porter S.R., Al-Johani K., Fedele S., Moles D.R. (2009). Randomised controlled trial of the efficacy of HybenX in the symptomatic treatment of recurrent aphthous stomatitis. Oral Dis..

[77] Lombardo D.G., Lombardo G., Signoretto C., Corrocher G., Pardo A., Pighi J., Rovera A., Caccuri F., Nocini P.F. (2015). A topical desiccant agent in association with ultrasonic debridement in the initial treatment of chronic periodontitis: A clinical and microbiological study. New Microbiol..

[78] Goodson J.M. (2003). Gingival crevice fluid flow. Periodontology 2000.

[79] Lesaffre E., Philstrom B., Needleman I., Worthington H. (2009). The design and analysis of split-mouth studies: What statisticians and clinicians should know. Stat. Med..

[80] Greenstein G. (2003). Clinical versus statistical significance as they relate to the efficacy of periodontal therapy. J. Am. Dent. Assoc..

[81] Fethney J. (2010). Statistical and clinical significance, and how to use confidence intervals to help interpret both. Aust. Crit. Care.

[82] Addy M., Newcombe R.G. (2005). Statistical versus clinical significance in periodontal research and practice. Periodontology 2000.

[83] Drisko C.L. (2014). Periodontal debridement: Still the treatment of choice. J. Evid. Based. Dent. Pract..

[84] Gerritsen A.E., Allen P.F., Witter D.J., Bronkhorst E.M., Creugers N.H.J. (2010). Tooth loss and oral health-related quality of life: A systematic review and meta-analysis. Health Qual. Life Outcomes.

[85] Tu Y.K., Gilthorpe M.S. (2012). Key statistical and analytical issues for evaluating treatment effects in periodontal research. Periodontology 2000.

