# Supplementary material for: Clinical Efficacy of Single Application Local Drug Delivery and Adjunctive Agents in Nonsurgical Periodontal Therapy: A Systematic Review and Network Meta-Analysis
Source: Pharmaceutics. 2020 Nov 12;12(11):1086. doi: 10.3390/pharmaceutics12111086 (PMC7698182; doi:10.3390/pharmaceutics12111086)
Supplement: Supplementary file 1 [file pharmaceutics-12-01086-s001.pdf]

pharmaceutics

MDPI

Supplementary Materials: Clinical Efficacy of Single Application Local Drug Delivery and Adjunctive Agents in Nonsurgical Periodontal Therapy: A Systematic Review and Network Meta-Analysis

Oi Leng Tan, Syarida Hasnur Safii and Masfueh Razali *

S1. Search strategies for each electronic database

PubMed (1979 to 31st January 2020)

1. (((((periodontal diseases [mesh]) NOT periodontal cyst [mesh]) NOT gingival diseases [mesh]) NOT periapical diseases [mesh]) NOT peri-implantitis [mesh]) OR periodontitis [tw]
2. (dental prophylaxis [mesh] OR subgingival curettage [mesh] OR dental prophylaxis [tw] OR scaling root planing [tw] OR dental scaling [tw] OR root planing [tw] OR dental cleaning [tw] OR SRP [tw] OR scaling planing [tw] OR ultrasonic therapy [tw] OR ultrasonic therapy [mesh] OR ultrasonic scaling [tw] OR root surface debridement [tw] OR root surface instrumentation [tw] OR periodontal debridement [tw] OR nonsurgical [tw] OR non-surgical [tw]) NOT (ultrasonic surgical procedures [mesh])
3. (randomized controlled trial [pt] OR controlled clinical trial [pt] OR randomized [tiab] OR placebo [tiab] OR clinical trials as topic [mesh:noexp] OR randomly [tiab] OR trial [ti] NOT (animals [mh] NOT humans [mh])) NOT (review [pt] OR systematic review [pt]) [Adapted from Lefebvre et al. (2019)]
4. (adjunct* [tw]) AND (dental enamel proteins [mh] OR enamel matrix derivative* [tw] OR Anti-infective agents [mh] OR antibiotic* [tw] OR antiseptic* [tw] OR antimicrobial* [tw] OR antibacterial* [tw] OR tetracycline [tw] OR chlorhexidine [tw] OR doxycycline [tw] OR metronidazole [tw] OR minocycline [tw] OR hyaluron* [tw] OR probiotic* [tw] OR photodynamic therap* [tw])
5. 1 and 2 and 3 and 4

MEDLINE via OVID (1946 to 31st January 2020)

1. exp Periodontal Diseases/ or periodonti$.mp. or (periodontal adj3 pocket$).mp. or "periodont$ attachment loss".mp.
2. exp Dental Prophylaxis/ or exp subgingival curettage/ or exp ultrasonic therapy/ or ((dental or oral or teeth or tooth or subgingival) adj6 (scaling or scale$ or curettage)).mp. or (dental prophylaxis or oral prophylaxis).mp. or (root adj (plane$ or planing)).mp. or (periodontal adj debridement$).mp. or (root surface adj debridement$).mp. or (root surface adj instrumentation$).mp. or (non?surgical adj3 periodontal).mp.
3. adjunct$.mp.
4. exp Anti-infective agents/ or (anti?biotic$ or anti?septic$ or anti?bacterial$ or anti?microbial$).mp. or (tetracycline or chlorhexidine or doxycycline or metronidazole or minocycline or hyaluron$ or photodynamic or enamel matrix derivative$ or probiotic$).mp.

5. randomized controlled trial.pt. or controlled clinical trial.pt. or randomized.ab. or placebo.ab. or drug therapy.fs. or randomly.ab. or trial.ab. or groups.ab. NOT (review.pt or systematic review.pt) [Adapted from Lefebvre et al. (2019)]
6. 1 and 2 and 3 and 4 and 5
7. (exp animal/ or animal.hw. or nonhuman/) not humans.sh.
8. 6 not 7

EMBASE via OVID (1974 to 31st January 2020)

1. exp Periodontal Diseases/ or periodonti$.mp. or (periodontal adj3 pocket$).mp. or "periodont$ attachment loss".mp.
2. exp Dental Prophylaxis/ or exp subgingival curettage/ or exp ultrasonic therapy/ or ((dental or oral or teeth or tooth or subgingival) adj6 (scaling or scale$ or curettage)).mp. or (dental prophylaxis or oral prophylaxis).mp. or (root adj (plane$ or planing)).mp. or (periodontal adj debridement$).mp. or (root surface adj debridement$).mp. or (root surface adj instrumentation$).mp. or (non?surgical adj3 periodontal).mp.
3. adjunct$.mp.
4. exp Anti-infective agents/ or (anti?biotic$ or anti?septic$ or anti?bacterial$ or anti?microbial$).mp. or (tetracycline or chlorhexidine or doxycycline or metronidazole or minocycline or hyaluron$ or photodynamic or enamel matrix derivative$ or probiotic$).mp.
5. Randomized controlled trial/ or controlled clinical study/ or random$.ti,ab. or randomisation/ or intermethod comparison/ or placebo.ti,ab. or (compare or compared or comparison).ti. or ((evaluated or evaluate or evaluating or assessed or assess) and (compare or compared or comparing or comparison)).ab. or (open adj label).ti,ab. or ((double or single or doubly or singly) adj (blind or blinded or blindly)).ti,ab. or double blind procedure/ or parallel group$1.ti,ab. or (crossover or cross over).ti,ab. or ((assign$ or match or matched or allocation) adj5 (alternate or group$1 or intervention$1 or patient$1 or subject$1 or participant$1)).ti,ab. or (assigned or allocated).ti,ab. or (controlled adj7 (study or design or trial)).ti,ab. or (volunteer or volunteers).ti,ab. or human experiment/ or trial.ti. [Adapted from Lefebvre et al. (2019)]
6. random$ adj sampl$ adj7 (cross section$ or questionnaire$1 or survey$ or database$1).ti,ab. not (comparative study/ or controlled study/ or randomi?ed controlled.ti,ab. or randomly assigned.ti,ab.) or Cross-sectional study/ not (randomized controlled trial/ or controlled clinical study/ or controlled study/ or randomi?ed controlled.ti,ab. or control group$1.ti,ab.) or (((case adj control$) and random$) not randomi?ed controlled).ti,ab. or (Systematic review not (trial or study)).ti. or (nonrandom$ not random$).ti,ab. or (Random field$).ti,ab. or (random cluster adj3 sampl$).ti,ab. or (review.ab. and review.pt.) not trial.ti. or (we searched).ab. and (review.ti. or review.pt.) or (update review).ab. or (databases adj4 searched).ab. or (rat or rats or mouse or mice or swine or porcine or murine or sheep or lambs or pigs or piglets or rabbit or rabbits or cat or cats or dog or dogs or cattle or bovine or monkey or monkeys or trout or marmoset$1).ti. and animal experiment/ or Animal experiment/ not (human experiment/ or human/)
7. 5 not 6
8. 1 and 2 and 3 and 4 and 7

Cochrane Central Register of Controlled Clinical Trials (CENTRAL) (inception to 31st January 2020)

1. MeSH descriptor: [Periodontal Diseases] explode all trees

2. (periodonti*) OR (periodontal near/3 pocket*) OR (periodont* attachment loss)
3. #1 or #2
4. MeSH descriptor: [Dental Prophylaxis] explode all trees
5. (dental or oral or teeth or tooth or subgingival) AND (scaling or scale* or curettage)
6. (dental prophylaxis OR oral prophylaxis) OR (root* NEXT plane* or root* NEXT planing) OR (periodontal NEXT debridement*)
7. (root surface NEXT debridement*) OR (root surface NEXT instrumentation*) OR ((non surgical or non-surgical or nonsurgical) near/3 periodontal)
8. #4 or #5 or #6 or #7
9. (adjunct*)
10. MeSH descriptor: [Anti-Infective Agents] explode all trees
11. (anti biotic* or anti septic* or anti bacterial* or anti microbial* or antibiotic* or antiseptic* or antibacterial* or antimicrobial* or anti-biotic* or anti-septic* or anti-bacterial* or anti-microbial*) OR (tetracycline or chlorhexidine or doxycycline or metronidazole or minocycline or hyaluron* or photodynamic or enamel matrix derivative* or probiotic*)
12. #10 or #11
13. #3 and #8 and #9 and #12

Web of Science (All databases) (1980 to 31st January 2020)

1. TS=((periodonti*) OR (periodontal near/3 pocket*) OR (periodont* attachment loss))
2. TS=((dental or oral or teeth or tooth or subgingival) AND (scaling or scale* or curettage))
3. TS=((dental prophylaxis OR oral prophylaxis) OR (root* NEAR/1 plane* or root* NEAR/1 planing) OR (periodontal NEAR/1 debridement*))
4. TS=((root surface debridement*) OR (root surface instrumentation*) or (non surgical or non-surgical or nonsurgical))
5. #4 OR #3 OR #2
6. TS=(anti biotic* or anti septic* or anti bacterial* or anti microbial* or antibiotic* or antiseptic* or antibacterial* or antimicrobial* or anti-biotic* or anti-septic* or anti-bacterial* or anti-microbial*)
7. TS=(tetracycline or chlorhexidine or doxycycline or metronidazole or minocycline or hyaluron* or photodynamic or enamel matrix derivative* or probiotic*)
8. #7 OR #6
9. TS=(adjunct*)
10. TS=(randomized controlled trial or randomised controlled clinical trial OR random* OR placebo OR clinical trial*)
11. TI=(review)
12. #10 AND #9 AND #8 AND #5 AND #1
13. #12 NOT #11
14. TS=(animal* NOT human*)
15. #13 NOT #14

US National Institutes of Health Ongoing Trials Register (ClinicalTrials.gov) (inception to 31st January 2020)

Advanced search:
Condition: periodontitis

Other terms: adjunct
Applied filter: Interventional

World Health Organization International Clinical Trials Registry Platform (inception to 31st January 2020)

Periodontitis AND adjunct

ProQuest Dissertations and Theses Global (1879 to 31st January 2020)

1. ((periodonti*) OR ("periodontal near/3 pocket*") OR ("periodont* attachment loss")) NOT ti(implant* or gingivitis or mucositis or orthodont* or endodont*)
2. ("dental prophylaxis" OR "oral prophylaxis") OR ("root* NEAR/1 plane*" OR "root* NEAR/1 planing") OR ("periodontal NEAR/1 debridement*") OR ("root surface debridement*") OR ("root surface instrumentation*") OR ("non surgical" or "non-surgical" or "nonsurgical") OR (dental or oral or teeth or tooth or subgingival) AND (scaling or scale* or curettage)
3. adjunct*
4. (anti?biotic* or anti?septic* or anti?bacterial* or anti?microbial*) OR (tetracycline or chlorhexidine or doxycycline or metronidazole or minocycline or hyaluron* or photodynamic or enamel matrix derivative* or probiotic*)
5. randomi?ed control* trial OR random* OR placebo OR clinical trial*
6. ti(review OR (in?vitro) OR (ex?vivo) OR (in?vivo) OR (cross?section*) OR retrospective OR observational OR experiment* OR literature)
7. s1 and s2 and s3 and s4 and s5
8. s7 not s6
9. su((animal*) NOT (human*))
10. s8 not s9

Table S1. Studies excluded after full-text screening and reasons for exclusion.

No	Author(s)	Year	Title of study	Journal	Volume	Issue	Page	Exclusion reason(s)
1	Ahamed, S.; Jalaluddin, M.; Khalid, I.; Moon, N.; Shaf, T. K.; Ali, F. M.	2013	The use of controlled release locally delivered 10% doxycycline hyclate gel as an adjunct to scaling and root planing in the treatment of chronic periodontitis: clinical and microbiological results	Journal of Contemporary Dental Practice	14	6	1080-1086	No randomization
2	Aimetti, M.; Romano, F.; Guzzi, N.; Carnevale, G.	2011	One-stage full-mouth disinfection as a therapeutic approach for generalized aggressive periodontitis	Journal of Periodontology	82	6	845-853	No control
3	Aimetti, M.; Romano, F.; Torta, I.; Cirillo, D.; Caposio, P.; Romagnoli, R.	2004	Debridement and local application of tetracycline-loaded fibers in the management of persistent periodontitis: Results after 12 months	Journal of Clinical Periodontology	31	3	166-172	Discontinued adjunct
4	Akıncıbay, H., Şenel, S., and Ay, Z. Y.	2007	Application of chitosan gel in the treatment of chronic periodontitis	Journal of Biomedical Materials Research. Part B: Applied Biomaterials	80	2	290-296	Adjunct not commercially available
5	Al Habashneh, R., Mashal, M. A., Khader, Y., and Qudah, R.	2019	Clinical and biological effects of adjunctive photodynamic therapy in refractory periodontitis	Journal of Lasers in Medical Sciences	10	2	139-145	No randomization
6	Balata, M. L., Andrade, L. P. D., Santos, D. B. N., Cavalcanti, A. N., Tunes, U. D. R., Ribeiro, E. D. P., and Bittencourt, S.	2013	Photodynamic therapy associated with full-mouth ultrasonic debridement in the treatment of severe chronic periodontitis: a randomized-controlled clinical trial	Journal of Applied Oral Science: Revista FOB	21	2	208-214	Failing sites retreated per initial basic therapy
7	Bizzarro, S., Van der Velden, U., and Loos, B. G.	2016	Local disinfection with sodium hypochlorite as adjunct to basic periodontal therapy: A randomized controlled trial	Journal of Clinical Periodontology	43	9	778-788	Used systemic antimicrobials
8	Blomlöf, L., Bergman, E., Forsgårdh, Å., Foss, L., Larsson, A., Sjöberg, B., Uhlander, L., et al.	2000	A clinical study of root surface conditioning with an EDTA gel. I. Nonsurgical periodontal treatment	International Journal of Periodontics & Restorative Dentistry	20	6	561-565	Experimental adjunct

9	Bogren, A., Teles, R. P., Torresyap, G., Haffajee, A. D., Socransky, S. S., and Wennström, J. L.	2008	Locally delivered doxycycline during supportive periodontal therapy: a 3-year study	Journal of Periodontology	79	5	827-835	Repeated application
10	Bosco, J., Lopes, B., Bosco, A. F., Spolidorio, D. M., and Marcantonio, R. A.	2009	Local application of tetracycline solution with a microbrush: An alternative treatment for persistent periodontitis	Quintessence International	40	1	29-40	Adjunct not commercially available
11	Bundidpun, P., Srisuwantha, R., and Laosrisin, N.	2018	Clinical effects of photodynamic therapy as an adjunct to full-mouth ultrasonic scaling and root planing in treatment of chronic periodontitis	Laser Therapy	27	1	33-39	Adjuncts administered one week after SRP
12	Cappuyns, I., Cionca, N., Wick, P., Giannopoulou, C., and Mombelli, A.	2012	Treatment of residual pockets with photodynamic therapy, diode laser, or deep scaling. A randomized, split-mouth controlled clinical trial	Lasers in Medical Science	27	5	979-986	Not used as adjunct
13	Carvalho, J., Novak, M. J., and Mota, L. F.	2007	Evaluation of the effect of subgingival placement of chlorhexidine chips as an adjunct to scaling and root planing	Journal of Periodontology	78	6	997-1001	Repeated application
14	Carvalho, V.F., Andrade, P.V., Rodrigues, M.F., Hirata, M.H., Hirata, R.D., Pannuti, C.M., De Micheli, G. et al.	2015	Antimicrobial photodynamic effect to treat residual pockets in periodontal patients: a randomized controlled clinical trial	Journal of Clinical Periodontology	42	5	440-447	Not used as adjunct
15	Chiang, C. P., Hsieh, O., Tai, W. C., Chen, Y. J., and Chang, P. C.	2019	Clinical outcomes of adjunctive indocyanine green-diode lasers therapy for treating refractory periodontitis: A randomized controlled trial with in vitro assessment	Journal of The Formosan Medical Association				Incomplete data
16	Cortelli, J.R., Aquino, D.R., Cortelli, S.C., Carvalho-F, J., Roman-Torres, C.V., and Costa, F.O.	2008	A double-blind randomized clinical trial of subgingival minocycline for chronic periodontitis	Journal of Oral Science	50	3	259-265	No CAL data
17	Cortelli, J. R., Querido, S. M. R., Aquino, D. R., Ricardo, L. H., and Pallos, D.	2006	Longitudinal clinical evaluation of adjunct minocycline in the treatment of chronic periodontitis	Journal of Periodontology	77	2	161-166	No CAL data
18	Cortelli, S. C., Cavallini, F., Alves, M. F. R., Bezerra, A.	2009	Clinical and microbiological effects of an essential-oil-containing mouth rinse applied in the "one-stage full-mouth disinfection"	Clinical Oral Investigations	13	2	189-194	No CAL data

	A., Queiroz, C. S., and Cortelli, J. R.		protocol—a randomized doubled-blinded preliminary study					
19	Cortelli, S.C., Cortelli, J.R., Holzhausen, M., Franco, G.C.N., Rebelo, R.Z., Sonagere, A.S., Queiroz, C.D.S. et al.	2009	Essential oils in one-stage full-mouth disinfection: double-blind, randomized clinical trial of long-term clinical, microbial, and salivary effects	Journal of Clinical Periodontology	36	4	333-342	No CAL data
20	Cosyn, Jan; Wyn, Iris; De Rouck, Tim; Sabzevar, Mehran Moradi	2006	Long-term clinical effects of a chlorhexidine varnish implemented treatment strategy for chronic periodontitis	Journal of Periodontology	77	3	406-415	No CAL data
21	da Cruz Andrade, P.V., Alves, V.T.E., de Carvalho, V.F., Rodrigues, M.D.F., Pannuti, C.M., Holzhausen, M., De Micheli, G. et al.	2017	Photodynamic therapy decrease immune-inflammatory mediators levels during periodontal maintenance	Lasers in Medical Science	32	1	9-17	Not used as adjunct
22	D'aiuto, F., Parkar, M., Nibali, L., Suvan, J., Lessem, J., and Tonetti, M. S.	2006	Periodontal infections cause changes in traditional and novel cardiovascular risk factors: Results from a randomized controlled clinical trial	American Heart Journal	151	5	977-984	No PPD/CAL data
23	Dannewitz, B., Lippert, K., Lang, N. P., Tonetti, M. S., and Eickholz, P.	2009	Supportive periodontal therapy of furcation sites: Non-surgical instrumentation with or without topical doxycycline	Journal of Clinical Periodontology	36	6	514-522	Subset of Tonetti 2012
24	De Lissovoy, G., Rentz, A. M., Dukes, E. M., Eaton, C. A., Jeffcoat, M. K., Killoy, W. J., and Finkelman, R. D.	1999	The cost-effectiveness of a new chlorhexidine delivery system in the treatment of adult periodontitis	Journal of the American Dental Association	130	6	855-862	Extension of Jeffcoat 1998
25	Dean, J. W., Branch-Mays, G. L., Hart, T. C., Reinhardt, R. A., Shapiro, B., Santucci, E. A., and Lessem, J.	2003	Topically applied minocycline microspheres: why it works	Compendium of Continuing Education in Dentistry	24	4	247-258	No control
26	Del Peloso Ribeiro, É., Bittencourt, S., Ambrosano, G. M. B., Nociti Jr, F. H., Sallum, E. A., Sallum, A. W., and Casati, M. Z.	2006	Povidone–iodine used as an adjunct to non-surgical treatment of furcation involvements	Journal of Periodontology	77	2	211-217	Failing sites retreated per initial basic therapy

27	Drisko, C.L., Cobb, C.M., Killoy, W.J., Michalowicz, B.S., Pihlstrom, B.L., Lowenguth, R.A., Caton, J.G., et al.	1995	Evaluation of periodontal treatments using controlled-release tetracycline fibers: clinical response	Journal of Periodontology	66	8	692-699	Discontinued adjunct
28	DRKS00005389	2014	Clinical outcomes of adjunctive antimicrobial photodynamic therapy in the treatment of patients with severe chronic periodontitis—a controlled randomized clinical trial	http://www.who.int/trialsearch/trial2.aspx?trialid=drks00005389	Incomplete data			
29	Eickholz, P., Kim, T.S., Bürklin, T., Schacher, B., Renggli, H.H., Schaecken, M.T., Holle, R., et al.	2002	Non-surgical periodontal therapy with adjunctive topical doxycycline: a double-blind randomized controlled multicenter study	Journal of Clinical Periodontology	29	2	108-117	15% DH not commercially available
30	Feng, H.S., Bernardo, C.C., Sonoda, L.L., Hayashi, F., Romito, G.A., De Lima, L.A.P.A., Lotufo, R.F.M. et al.	2011	Subgingival ultrasonic instrumentation of residual pockets irrigated with essential oils: a randomized controlled trial	Journal of Clinical Periodontology	38	7	637-643	Adjuncts administered one week after SRP
31	Flemmig, T. F., Weinacht, S., Rüdiger, S., Rumetsch, M., Jung, A., and Klaiber, B.	1996	Adjunctive controlled topical application of tetracycline HCl in the treatment of localized persistent or recurrent periodontitis: Effects on clinical parameters and elastase-α1-proteinase inhibitor in gingival crevicular fluid	Journal of Clinical Periodontology	23	10	914-921	Discontinued adjunct
32	Friesen, L. R., Williams, K. B., Krause, L. S., and Killoy, W. J.	2002	Controlled local delivery of tetracycline with polymer strips in the treatment of periodontitis	Journal of Periodontology	73	1	13-19	Adjunct not commercially available
33	Fulvi, L., Polizzi, E. and Calderini, A.	2011	Effectiveness of local antibiotic therapy plus scaling/root-planing: An experimental split-mouth study	Prevenzione E Assistenza Dentale	37	2	33-43	Adjunct not commercially available
34	Garrett, S., Johnson, L., Drisko, C.H., Adams, D.F., Bandt, C., Beiswanger, B., Bogle, G., et al.	1999	Two multi-center studies evaluating locally delivered doxycycline hyclate, placebo control, oral hygiene, and scaling and root planing in the treatment of periodontitis	Journal of Periodontology	70	5	490-503	Not used as adjunct
35	Garrett, S., Adams, D.F., Bogle, G., Donly, K., Hastings Drisko, C.,	2000	The effect of locally delivered controlled-release doxycycline or scaling and root planing on periodontal maintenance patients over 9 months	Journal of Periodontology	71	1	22-30	Subset of Garrett 1999

	Hallmon, W.W., Brady Hancock, E., et al.							
36	Giannelli, M., Formigli, L., Lorenzini, L., and Bani, D.	2012	Combined photoablative and photodynamic diode laser therapy as an adjunct to non-surgical periodontal treatment. A randomized split-mouth clinical trial	Journal of Clinical Periodontology	39	10	962-970	Adjunct administered one week after SRP
37	Giannelli, M., Formigli, L., Lorenzini, L., and Bani, D.	2015	Efficacy of combined photoablative-photodynamic diode laser therapy adjunctive to scaling and root planing in periodontitis: randomized split-mouth trial with 4-year follow-up	Photomedicine and Laser Surgery	33	9	473-480	Adjunct administered one week after SRP
38	Giannelli, M., Materassi, F., Fossi, T., Lorenzini, L., and Bani, D.	2018	Treatment of severe periodontitis with a laser and light-emitting diode (LED) procedure adjunctive to scaling and root planing: a double-blind, randomized, single-center, split-mouth clinical trial investigating its efficacy and patient-reported outcomes at 1 year	Lasers in Medical Science	33	5	991-1002	Adjunct administered one week after SRP
39	Giannopoulou, C., Cappuyns, I., Cancela, J., Cionca, N., and Mombelli, A.	2012	Effect of photodynamic therapy, diode laser, and deep scaling on cytokine and acute-phase protein levels in gingival crevicular fluid of residual periodontal pockets	Journal of Periodontology	83	8	1018-1027	Not used as adjunct
40	Goh, E. X., Tan, K. S., Chan, Y. H., and Lim, L. P.	2017	Effects of root debridement and adjunctive photodynamic therapy in residual pockets of patients on supportive periodontal therapy: A randomized split-mouth trial	Photodiagnosis And Photodynamic Therapy	18		342-348	Failing sites retreated per initial basic therapy
41	Gonzales, J. R., Harnack, L., Schmitt-Corsitto, G., Boedeker, R. H., Chakraborty, T., Domann, E., and Meyle, J.	2011	A novel approach to the use of subgingival controlled-release chlorhexidine delivery in chronic periodontitis: A randomized clinical trial	Journal of Periodontology	82	8	1131-1139	Adjunct administered before SRP
42	Gopinath, V., Ramakrishnan, T., Emmadi, P., Ambalavanan, N., and Mammen, B.	2009	Effect of a controlled release device containing minocycline microspheres on the treatment of chronic periodontitis: A comparative study	Journal of Indian Society of Periodontology	13	2	79	No mention of method of randomization
43	Grisi, D. C., Salvador, S. L., Figueiredo, L. C., Souza, S. L., Novaes Jr, A. B., and Grisi, M. F.	2002	Effect of a controlled-release chlorhexidine chip on clinical and microbiological parameters of periodontal syndrome	Journal of clinical periodontology	29	10	875-881	Repeated application

44	Grzech-Leśniak, K., Gaspirc, B., and Sculean, A.	2019	Clinical and microbiological effects of multiple applications of antibacterial photodynamic therapy in periodontal maintenance patients. A randomized controlled clinical study	Photodiagnosis And Photodynamic Therapy	27		44-50	No CAL data
45	Hagiwara, S., Iida, M., and Ishikawa, I.	1993	Clinical and microbiological study concerning effects of scaling and root planing. 2. The effects of mechanical debridement until one year and of minocycline application topically	Kokubyo Gakkai Zasshi. The Journal of The Stomatological Society, Japan	60	2	301-312	No mention of method of randomization
46	Harmouche, L., Courval, A., Mathieu, A., Petit, C., Huck, O., Severac, F., and Davideau, J. L.	2019	Impact of tooth-related factors on photodynamic therapy effectiveness during active periodontal therapy: A 6-month split-mouth randomized clinical trial	Photodiagnosis and photodynamic therapy	27		167-172	Repeated application
47	Henke, C.J.; Genco, R.J.; Killoy, W.J.; Miller, D.P.; Evans, C.J.; Finkelman, R.D.; Villa, K.F. et al.	2001	An economic evaluation of a chlorhexidine chip for treating chronic periodontitis: the CHIP (chlorhexidine in periodontitis) study	Journal of The American Dental Association (1939)	132	11	1557-1569	No final PPD/CAL reported
48	Ioannou, I., Dimitriadis, N., Papadimitriou, K., Vouros, I., Sakellari, D., and Konstantinidis, A.	2011	The effect of locally delivered doxycycline in the treatment of chronic periodontitis. A clinical and microbiological cohort study	Journal of Oral & Maxillofacial Research	1	4	e1	No randomization, no control
49	Jentsch, H.R., Roccuzzo, M., Marini, L., Kasaj, A., Fimmers, R., Jepsen, S.	2018	0059: Flapless application of enamel matrix derivative (EMD) as an adjunct to scaling and root planing – a multi-center RCT	Journal of Clinical Periodontology	45		26-27	Less than 6 months duration
50	Jeffcoat, M. K., Palcanis, K. G., Weatherford, T. W., Reese, M., Geurs, N. C., and Flashner, M.	2000	Use of a biodegradable chlorhexidine chip in the treatment of adult periodontitis: clinical and radiographic findings	Journal of Periodontology	71	2	256-262	Subset of Jeffcoat 1998
51	Jeffcoat, M.K., Bray, K.S., Ciancio, S.G., Dentino, A.R., Fine, D.H., Gordon, J.M., Gunsolley, J.C., et al.	1998	Adjunctive use of a subgingival controlled-release chlorhexidine chip reduces probing depth and improves attachment level compared with scaling and root planing alone	Journal of Periodontology	69	9	989-997	Only adjunct repeated, not SRP; possible bias toward adjunct
52	Jones, A. A., Kornman, K. S., Newbold, D. A., and Manwell, M. A.	1994	Clinical and microbiological effects of controlled-release locally delivered minocycline in periodontitis	Journal of Periodontology	65	11	1058-1066	Adjunct not commercially available

53	Kanoriya, D., Singhal, S., Garg, V., Pradeep, A. R., Garg, S., and Kumar, A.	2018	Clinical efficacy of subgingivally delivered 0.75% boric acid gel as an adjunct to mechanotherapy in chronic periodontitis: A randomized, controlled clinical trial	Journal of Investigative and Clinical Dentistry	9	e1227 1		Adjunct not commercially available
54	Kasaj, A., Chiriachide, A., and Willershausen, B.	2007	The adjunctive use of a controlled-release chlorhexidine chip following treatment with a new ultrasonic device in supportive periodontal therapy: A prospective, controlled clinical study	International Journal of Dental Hygiene	5	4	225-231	Repeated application
55	Katsikanis, F., Strakas, D., and Vouros, I.	2019	The application of antimicrobial photodynamic therapy (aPDT, 670 nm) and diode laser (940 nm) as adjunctive approach in the conventional cause-related treatment of chronic periodontal disease: A randomized controlled split-mouth clinical trial	Clinical Oral Investigations			1-7	Repeated application
56	Kerry, G.	1994	Tetracycline-loaded fibers as adjunctive treatment in periodontal disease	Journal of The American Dental Association (1939)	125	9	1199-1203	Discontinued adjunct
57	Killeen, A. C., Harn, J. A., Erickson, L. M., Yu, F., and Reinhardt, R. A.	2016	Local minocycline effect on inflammation and clinical attachment during periodontal maintenance: Randomized clinical trial	Journal of Periodontology	87	10	1149-1157	Original study of Killeen 2018
58	Killeen, A. C., Harn, J. A., Jensen, J., Yu, F., Custer, S., and Reinhardt, R. A.	2018	Two-year randomized clinical trial of adjunctive minocycline microspheres in periodontal maintenance	Journal of Dental Hygiene	92	4	51-58	Repeated application
59	Killoy, W. J.	1999	Assessing the effectiveness of locally delivered chlorhexidine in the treatment of periodontitis	Journal of The American Dental Association (1939)	130	4	567-570	Review
60	Kim, T. S., Burklin, T., Schacher, B., Ratka-Kruger, P., Schaecken, M. T., Renggli, H. H., and Eickholz, P.	2003	Local application of antibiotics versus mechanical debridement in supportive periodontal treatment	Deutsche Zahnarztliche Zeitschrift	58	10	579-583	Not used as adjunct
61	Kinane, D. F., and Radvar, M.	1999	A six-month comparison of three periodontal local antimicrobial therapies in persistent periodontal pockets	Journal of Periodontology	70	1	1-7	Discontinued adjunct
62	Kolbe, M.F., Ribeiro, F.V., Luchesi, V.H., Casarin, R.C., Sallum, E.A., Nociti	2014	Photodynamic therapy during supportive periodontal care: clinical, microbiologic, immunoinflammatory, and patient-centered	Journal of Periodontology	85	8	e277-e286	Not used as adjunct

	Jr, F.H., Ambrosano, G.M., et al.		performance in a split-mouth randomized clinical trial					
63	Kondreddy, K., Ambalavanan, N., Ramakrishna, T., and Kumar, R. S.	2012	Effectiveness of a controlled release chlorhexidine chip (PerioColTM-CG) as an adjunctive to scaling and root planing when compared to scaling and root planing alone in the treatment of chronic periodontitis: A comparative study	Journal of Indian Society of Periodontology	16	4	553-557	No randomization
64	Konuganti, K.; Kumar, A.	2016	Efficacy of subgingivally delivered flurbiprofen and chlorhexidine chip in the treatment of chronic periodontitis: A randomized controlled clinical trial	International Journal of Pharmaceutical Sciences Review and Research	40	1	149-153	Adjunct not commercially available
65	Koshy, G., Kawashima, Y., Kiji, M., Nitta, H., Umeda, M., Nagasawa, T., and Ishikawa, I.	2005	Effects of single-visit full-mouth ultrasonic debridement versus quadrant-wise ultrasonic debridement	Journal of Clinical Periodontology	32	7	734-743	No passive control
66	Laleman, I., Pauwels, M., Quirynen, M., and Teughels, W.	2020	A dual-strain Lactobacilli reuteri probiotic improves the treatment of residual pockets: A randomized controlled clinical trial	Journal of Clinical Periodontology	47		43-53	Administered use of lozenges at home
67	Lauenstein, M., Kaufmann, M., and Persson, G. R.	2013	Clinical and microbiological results following nonsurgical periodontal therapy with or without local administration of piperacillin/tazobactam	Clinical Oral Investigations	17	7	1645-1660	No CAL data
68	Leonhardt, Å., Bergström, C., Krok, L., and Cardaropoli, G.	2006	Healing following ultrasonic debridement and PVP–iodine in individuals with severe chronic periodontal disease: A randomized, controlled clinical study	Acta Odontologica Scandinavica	64	5	262-266	PD in %
69	Leonhardt, Å., Bergström, C., Krok, L., and Cardaropoli, G.	2007	Microbiological effect of the use of an ultrasonic device and iodine irrigation in patients with severe chronic periodontal disease: A randomized controlled clinical study	Acta Odontologica Scandinavica	65	1	52-59	No PPD/CAL data
70	Lie, T., Bruun, G., and Böe, O. E.	1998	Effects of topical metronidazole and tetracycline in treatment of adult periodontitis	Journal of Periodontology	69	7	819-827	Discontinued adjunct
71	Lulic, M., Leiggener Görög, I., Salvi, G. E., Ramseier, C.	2009	One-year outcomes of repeated adjunctive photodynamic therapy during periodontal	Journal of Clinical Periodontology	36	8	661-666	Repeated application

	A., Mattheos, N., and Lang, N. P.		maintenance: A proof-of-principle randomized-controlled clinical trial					
72	Machion, L., Andia, D. C., Lecio, G., Nociti Jr, F. H., Casati, M. Z., Sallum, A. W., and Sallum, E. A.	2006	Locally delivered doxycycline as an adjunctive therapy to scaling and root planing in the treatment of smokers: A 2-year follow-up	Journal of Periodontology	77	4	606-613	Extension of Machion 2004
73	McColl, E., Patel, K., Dahlen, G., Tonetti, M., Graziani, F., Suvan, J., and Laurell, L.	2006	Supportive periodontal therapy using mechanical instrumentation or 2% minocycline gel: A 12-month randomized, controlled, single masked pilot study	Journal of Clinical Periodontology	33	2	141-150	Not used as adjunct
74	Megally, A., Zekeridou, A., Cancela, J., Giannopoulou, C., and Mombelli, A.	2019	Short ultrasonic debridement with adjunctive low-concentrated hypochlorite/amino acid gel during periodontal maintenance: Randomized clinical trial of 12 months	Clinical Oral Investigations			1 - 9	Repeated application
75	Meinberg, T. A., Barnes, C. M., Dunning, D. G., and Reinhardt, R. A.	2002	Comparison of conventional periodontal maintenance versus scaling and root planing with subgingival minocycline	Journal of Periodontology	73	2	167-172	No randomization
76	Mızrak, T., Güncü, G. N., Çağlayan, F., Balcı, T. A., Aktar, G. S., and İpek, F.	2006	Effect of a controlled-release chlorhexidine chip on clinical and microbiological parameters and prostaglandin E2 levels in gingival crevicular fluid	Journal of Periodontology	77	3	437-443	Repeated application
77	Modanese, D. D. G., Tiosso-Tamburi, R., de Goes, V. F. F., de Cássia Bergamaschi, C., Martinez, E. F., Napimoga, M. H., et al.	2016	Clinical and immunoinflammatory evaluation of one-stage full-mouth ultrasonic debridement as a therapeutic approach for smokers with generalized aggressive periodontitis: A short-term follow-up study	Journal of Periodontology	87	9	1012-1021	Not used as adjunct
78	Mongardini, C., van Steenberghe, D., Dekeyser, C., and Quirynen, M.	1999	One stage full- versus partial-mouth disinfection in the treatment of chronic adult or generalized early-onset periodontitis. I. Long-term clinical observations	Journal of Periodontology	70	6	632-645	Comparison of different SRP
79	Müller, N., Moëne, R., Cancela, J. A., and Mombelli, A.	2014	Subgingival air-polishing with erythritol during periodontal maintenance: Randomized clinical trial of twelve months	Journal of Clinical Periodontology	41	9	883-889	CHX content not with the intention to have a therapeutic effect

80	NCT02487186	2015	Locally delivered doxycycline adjunct to nonsurgical periodontal therapy	https://clinicaltrials.gov/show/nct02487186	Incomplete data			
81	NCT04036890	2019	Local minocycline in patients under supportive periodontal therapy	https://clinicaltrials.gov/ct2/show/nct04036890	Incomplete data			
82	Newman, M. G., Kornman, K. S., and Doherty, F. M.	1994	A 6-month multi-center evaluation of adjunctive tetracycline fiber therapy used in conjunction with scaling and root planing in maintenance patients: Clinical results	Journal of Periodontology	65	7	685-691	Discontinued adjunct
83	Okuda, K., Wolff, L., Oliver, R., Osbom, J., Stoltenberg, J., Bereuter, J., Anderson, L., et al.	1992	Minocycline slow-release formulation effect on subgingival bacteria	Journal of Periodontology	63	2	73-79	No PPD/CAL data
84	Oringer, R. J., Van, T. D., and Lessem, J.	2002	The challenge of treating periodontal patients who smoke—the efficacy of Arestin	Journal of The International Academy of Periodontology	4	3	89-94	Mix subpopulation of 2 rcts
85	Paquette, D., Oringer, R., Lessem, J., Offenbacher, S., Genco, R., Persson, G.R., Santucci, E.A., et al.	2003	Locally delivered minocycline microspheres for the treatment of periodontitis in smokers	Journal of Clinical Periodontology	30	9	787-794	Subset of Williams 2001; no CAL data
86	Pedrazzoli, V., Kilian, M., and Karring, T.	1992	Comparative clinical and microbiological effects of topical subgingival application of metronidazole 25% dental gel and scaling in the treatment of adult periodontitis	Journal of Clinical Periodontology	19	9	715-722	Not used as adjunct
87	Pejčić, A., Kojović, D., Minić, I., Mirković, D., Denić, M., and Stojanović, M.	2015	Therapeutic efficacy of clindamycin gel as an adjunct to scaling and root planing therapy in chronic periodontal disease	Acta Clinica Croatica	54	1	46-50	Adjunct not commercially available
88	Petelin, M., Perkič, K., Seme, K., and Gašpirc, B.	2015	Effect of repeated adjunctive antimicrobial photodynamic therapy on subgingival periodontal pathogens in the treatment of chronic periodontitis	Lasers in Medical Science	30	6	1647-1656	Repeated application
89	Pietruska, M., Paniczko, A., Waszkiel, D., Pietruski, J., and Bernaczyk, A.	2006	Efficacy of local treatment with chlorhexidine gluconate drugs on the clinical status of periodontium in chronic periodontitis patients	Advances in Medical Sciences	51	7	162-165	Adjunct administered one week after SRP

90	Quirynen, M., De Soete, M., Boschmans, G., Pauwels, M., Coucke, W., Teughels, W., and Van Steenberghe, D.	2006	Benefit of "one-stage full-mouth disinfection" is explained by disinfection and root planing within 24 hours: A randomized controlled trial	Journal of Clinical Periodontology	33	9	639-647	Comparison of supragingival antiseptics
91	Quirynen, M., Mongardini, C., De Soete, M., Pauwels, M., Coucke, W., Van Eldere, J., and Van Steenberghe, D.	2000	The role of chlorhexidine in the one-stage full-mouth disinfection treatment of patients with advanced adult periodontitis. Long-term clinical and microbiological observations	Journal of Clinical Periodontology	27	8	578-589	Comparison of different SRP
92	Ratka-Krüger, P., Schacher, B., Bürklin, T., Böddinghaus, B., Holle, R., Renggli, H.H., Eickholz, P., et al.	2005	Non-surgical periodontal therapy with adjunctive topical doxycycline: a double-masked, randomized, controlled multicenter study. II. Microbiological results	Journal of Periodontology	76	1	66-74	No PPD/CAL data
93	Raut, C. P., Sethi, K. S., Kohale, B. R., Mamajiwala, A., and Warang, A.	2018	Indocyanine green-mediated photothermal therapy in treatment of chronic periodontitis: A clinico-microbiological study	Journal of Indian Society of Periodontology	22	3	221-227	Adjunct not commercially available
94	Renvert, S., Dahlén, G., and Snyder, B.	1997	Clinical and microbiological effects of subgingival antimicrobial irrigation with citric acid as evaluated by an enzyme immunoassay and culture analysis	Journal of Periodontology	68	4	346-352	No randomization
95	Renvert, S., Dahlen, G., and Wikström, M.	1996	Treatment of periodontal disease based on microbiological diagnosis. Relation between microbiological and clinical parameters during 5 years	Journal of Periodontology	67	6	562-571	Use of systemic antimicrobials
96	Ribeiro, É. D. P., Bittencourt, S., Sallum, E. A., Sallum, A. W., and Nociti Júnior, F. H.	2010	Non-surgical instrumentation associated with povidone–iodine in the treatment of interproximal furcation involvements	Journal of Applied Oral Science	18	6	599-606	Failing sites retreated per initial basic therapy
97	Rodrigues, I. F. G., Machion, L., Casati, M. Z., Nociti Jr, F. H., de Toledo, S., Sallum, A. W., and Sallum, E. A.	2007	Clinical evaluation of the use of locally delivered chlorhexidine in periodontal maintenance therapy	Journal of Periodontology	78	4	624-628	Adjunct administered 3 months after SRP
98	Romano, F., Torta, I., Debernardi, C., and Aimetti, M.	2005	Debridement and local application of tetracycline in the management of persistent	Minerva Stomatologica	54	1-2	43-51	Discontinued adjunct

			periodontitis. Clinical and microbiological results after 12 months					
99	Rosling, B. G., Slots, J., Christersson, L. A., Gröndahl, H. G., and Genco, R. J.	1986	Topical antimicrobial therapy and diagnosis of subgingival bacteria in the management of inflammatory periodontal disease	Journal of Clinical Periodontology	13	10	975-981	Trial series
100	Rosling, B., Hellström, M. K., Ramberg, P., Socransky, S. S., and Lindhe, J.	2001	The use of PVP–iodine as an adjunct to non-surgical treatment of chronic periodontitis	Journal of Clinical Periodontology	28	11	1023-1031	Failing sites retreated per initial basic therapy
101	Rudhart, A., Purucker, P., Kage, A., Hopfenmüller, W., and Bernimoulin, J. P.	1998	Local metronidazole application in maintenance patients. Clinical and microbiological evaluation	Journal of Periodontology	69	10	1148-1154	Not used as adjunct
102	Sakellari, D., Vouros, I., and Konstantinidis, A.	2003	The use of tetracycline fibers in the treatment of generalized aggressive periodontitis: clinical and microbiological findings	Journal of The International Academy of Periodontology	5	2	52-60	Discontinued adjunct
103	Santuchi, C. C., Cortelli, J. R., Cortelli, S. C., Cota, L. O. M., Fonseca, D. C., Alencar, C. O., and Costa, F. O.	2016	Scaling and root planing per quadrant versus one-stage full-mouth disinfection: Assessment of the impact of chronic periodontitis treatment on quality of life—A clinical randomized, controlled trial	Journal of Periodontology	87	2	114-123	Comparison of different SRP
104	Santuchi, C. C., Cortelli, S. C., Cortelli, J. R., Cota, L. O. M., Alencar, C. O., and Costa, F. O.	2015	Pre- and post-treatment experiences of fear, anxiety, and pain among chronic periodontitis patients treated by scaling and root planing per quadrant versus one-stage full-mouth disinfection: A 6-month randomized controlled clinical trial	Journal of Clinical Periodontology	42	11	1024-1031	Comparison of different SRP
105	Sculean, A., Windisch, P., Keglevich, T., and Gera, I.	2003	Histologic evaluation of human intrabony defects following non-surgical periodontal therapy with and without application of an enamel matrix protein derivative	Journal of Periodontology	74	2	153-160	Not RCT
106	Segarra-Vidal, M., Guerra-Ojeda, S., Vallés, L.S., López-Roldán, A., Mauricio, M.D., Aldasoro, M., Alpiste-Illueca, F. et al.	2017	Effects of photodynamic therapy in periodontal treatment: A randomized, controlled clinical trial	Journal of Clinical Periodontology	44	9	915-925	Adjunct administered one week after SRP

107	Shaddox, L. M., Andia, D. C., Casati, M. Z., Nociti, F. H., Sallum, E. A., Gollwitzer, J., and Walker, C. B.	2007	Microbiologic changes following administration of locally delivered doxycycline in smokers: A 15-month follow-up	Journal of Periodontology	78	11	2143-2149	Subset of Machion 2004, 2006
108	Shalev, A.	2019	Clinical efficacy of local delivery minocycline gel for the treatment of moderate to severe periodontitis	University of Minnesota Digital Conservancy, http://hdl.handle.net/11299/206137				Repeated application
109	Shiloah, J., and Patters, M. R.	1996	Repopulation of periodontal pockets by microbial pathogens in the absence of supportive therapy	Journal of Periodontology	67	2	130-139	CAL data not separated by groups
110	Singh, M., Shreehari, A. K., Garg, P. K., and Singh, S.	2014	Clinical efficacy of chlorhexidine chips and tetracycline fibers as an adjunct to nonsurgical periodontal therapy	European Journal of General Dentistry	3	2	134-139	Adjunct administered one week after SRP
111	Soeroso, Y., Akase, T., Sunarto, H., Kemal, Y., Salim, R., Octavia, M., Viandita, A., et al.	2017	The risk reduction of recurrent periodontal pathogens of local application minocycline HCl 2% gel, used as an adjunct to scaling and root planing for chronic periodontitis treatment	Therapeutics and Clinical Risk Management	13		307-314	Duplicate of Soeroso 2016 which is more complete
112	Soskolne, W. A., Heasman, P. A., Stabholz, A., Smart, G. J., Palmer, M., Flashner, M., and Newman, H. N.	1997	Sustained local delivery of chlorhexidine in the treatment of periodontitis: A multi-center study	Journal of Periodontology	68	1	32-38	Failing sites of control not retreated, bias toward adjunct?
113	Soskolne, W. A., Proskin, H. M., and Stabholz, A.	2003	Probing depth changes following 2 years of periodontal maintenance therapy including adjunctive controlled release of chlorhexidine	Journal of Periodontology	74	4	420-427	No randomization
114	Stelzel, M.	1997	Topical metronidazole application in recall patients: Long-term results	Journal of Clinical Periodontology	24	12	914-919	Not used as adjunct
115	Sweatha, C., Srikanth, C., and Babu, M. R.	2015	A comparative study of the effect of minocycline microspheres as an adjunct to scaling and root planing versus scaling and root planing alone in the treatment of chronic periodontitis	International Journal of Recent Scientific Research	6	4	3540-3550	Failing sites of control not retreated, bias towards adjunct?

116	Swierkot, K., Nonnenmacher, C. I., Mutters, R., Flores-de-Jacoby, L., and Mengel, R.	2009	One-stage full-mouth disinfection versus quadrant and full-mouth root planing	Journal of Clinical Periodontology	36	3	240-249	Comparison of different SRP
117	Tabenski, L., Moder, D., Cieplik, F., Schenke, F., Hiller, K.A., Buchalla, W., Schmalz, G. and Christgau, M.	2017	Antimicrobial photodynamic therapy vs. local minocycline in addition to non-surgical therapy of deep periodontal pockets: A controlled randomized clinical trial	Clinical Oral Investigations	21	7	2253-2264	Repeated application
118	Timmerman, M. F., Van der Weijden, G. A., Van Steenbergen, T. J. M., Mantel, M. S., De Graaff, J., and Van der Velden, U.	1996	Evaluation of the long-term efficacy and safety of locally-applied minocycline in adult periodontitis patients	Journal of Clinical Periodontology	23	8	707-716	Subset of van Steenberghe 1999
119	Trojahn, M. R. B., Silva, R. C. D., and Joly, J. C.	2013	Non-surgical periodontal therapy for the treatment of chronic periodontitis	Rgo. Revista Gaúcha De Odontologia (Online)	61	4	529-534	Adjunct not commercially available
120	Tomasi, C., and Wennström, J. L.	2011	Locally delivered doxycycline as an adjunct to mechanical debridement at retreatment of periodontal pockets: outcome at furcation sites	Journal of Periodontology	82	2	210-218	Subset of Tomasi 2008
121	Tonetti, M. S., Cortellini, P., Carnevale, G., Cattabriga, M., De Sanctis, M., and Prato, G. P.	1998	A controlled multi-center study of adjunctive use of tetracycline periodontal fibers in mandibular class II furcations with persistent bleeding	Journal of Clinical Periodontology	25	9	728-736	Discontinued adjunct
122	Tonetti, M.S., Lang, N.P., Cortellini, P., Suvan, J.E., Eickholz, P., Fourmousis, I., Topoll, H., et al.	2012	Effects of a single topical doxycycline administration adjunctive to mechanical debridement in patients with persistent/recurrent periodontitis but acceptable oral hygiene during supportive periodontal therapy	Journal of Clinical Periodontology	39	5	475-482	Failing sites retreated per initial basic therapy
123	Ueda, M., Teranishi, Y., Nakagaki, N., Imai, H., Katagiri, E. and Funaoka, K.	1995	Periodontal therapy by local delivery of antibiotics—Periocline (R) administration in periodontal pocket in combination with scaling—(Part 4)	Oral Therapeutics and Pharmacology	14	2	99-105	Duration less than 6 months
124	Ueda, M., Ogata, C., Suga, H., Kohno, T., Imai, H., Cao, C., Geng, S., et al.	2001	Periodontal therapy with local delivery of antibiotics: Part 1 Observation at Osaka Dental University	Shikaigaku	64	2	187-190	Duration less than 6 months

125	Ueda, M., Ogata, C., Suga, H., Kohno, T., Imai, H., Cao, C., Geng, S., et al.	2001	Periodontal therapy with local delivery of antibiotics: Part 2 Observation at Beijing University	Shikaigaku	64	2	191-194	Duration less than 6 months
126	Unsal, E., Akkaya, M., and Walsh, T. F.	1994	Influence of a single application of subgingival chlorhexidine gel or tetracycline paste on the clinical parameters of adult periodontitis patients	Journal of Clinical Periodontology	21	5	351-355	Duration less than 6 months
127	Van Steenberghe, D., Rosling, B., Söder, P.Ö., Landry, R.G., Van der Velden, U., Timmerman, M.F.T., McCarthy, E.F., Vandenhoven, G., Wouters, C., Wilson, M. and Matthews, J.	1999	A 15-month evaluation of the effects of repeated subgingival minocycline in chronic adult periodontitis	Journal of Periodontology	70	6	657-667	Repeated application
128	Vandekerckhove, B. N., Bollen, C. M., Dekeyser, C., Darius, P., and Quirynen, M.	1996	Full- versus partial-mouth disinfection in the treatment of periodontal infections. Long-term clinical observations of a pilot study	Journal of Periodontology	67	12	1251-1259	Comparison of different SRP
129	Vandekerckhove, B. N., Quirynen, M., and van Steenberghe, D.	1997	The use of tetracycline-containing controlled-release fibers in the treatment of refractory periodontitis	Journal of Periodontology	68	4	353-361	Discontinued adjunct
130	Vitt, A., Gustafsson, A., Ramberg, P., Slizen, V., Kazeko, L. A., and Buhlin, K.	2019	Polyhexamethylene guanidine phosphate irrigation as an adjunctive to scaling and root planing in the treatment of chronic periodontitis	Acta Odontologica Scandinavica	77	4	290-295	No CAL data
131	Wennström, J. L., Heijl, L., Dahlén, G., and Gröndahl, K.	1987	Periodic subgingival antimicrobial irrigation of periodontal pockets (I). Clinical observations	Journal of Clinical Periodontology	14	9	541-550	Adjunct placed before SRP
132	Wong, M. Y., Lu, C. L., Liu, C. M., and Hou, L. T.	1999	Microbiological response of localized sites with recurrent periodontitis in maintenance patients treated with tetracycline fibers	Journal of Periodontology	70	8	861-868	Discontinued adjunct
133	Wong, M. Y., Lu, C. L., Liu, C. M., Hou, L. T., and Chang, W. K.	1998	Clinical response of localized recurrent periodontitis treated with scaling, root planing, and tetracycline fiber	Journal of The Formosan Medical Association = Taiwan Yi Zhi	97	7	490-497	Discontinued adjunct

134	Zingale, J., Harpenau, L., Bruce, G., Chambers, D., and Lundergan, W.	2012	The effectiveness of scaling and root planing with adjunctive time-release minocycline using an open and closed approach for the treatment of periodontitis	General Dentistry	60	4	300-305	No CAL data, compared surgical procedure

Table S2. Study characteristics of included studies.

Study citation	Country	Trial design	Centre	Setting	Funding	Disease severity	Definition	Age Mean ± SD (range)		Sex Male/female		n smokers (%)
								Test	Control	Test	Control	
**Chlorhexidine chip**												
(Azmak et al. 2002)	Turkey	split-mouth	single	universit y	NR	moderate to severe chronic periodontitis	Nonadjacent interproxima l anterior sites with PPD 6-8 mm and BOP	NR (36-62)		NR		NS
(Heasman et al. 2001)	UK	split-mouth	single	universit y	NR	moderate to severe chronic periodontitis	PPD ≥ 5 mm and BOP	42.6 ± 12.6 (34–59)		8/18		NS
(Paolantonio et al. 2008a)	Italy	split-mouth	4	universit y	NR	moderate to advanced periodontitis	Interproxima l sites with PPD ≥ 5 mm and BOP	NR (33-65)		34/82		NS
(Paolantonio et al. 2008b)	Italy	split-mouth	single	universit y	NR	moderate to advanced periodontitis	Interproxima l sites with PPD ≥ 5 mm and BOP	NR (31-63)		33/49		NS
(Reddy et al. 2016)	India	parallel	single	universit y	NR	chronic periodontitis	Nonadjacent sites with PPD ≥ 5 mm and BOP	NR		NR		NS
(Sakellari et al. 2010)	Greece	parallel	single	universit y	commercial	generalized chronic periodontitis	Interproxima l single-rooted sites with PPD 5-7 mm	46.35 ± 7.31 (36-64)	48.75 ± 10.15 (37-75)	14/11	13/12	9/50 (18)

**Chlorhexidine varnish**												
(Cosyn et al. 2007)	Belgium	parallel	2	university and private practice	NR	chronic periodontitis	PPD ≥ 6 mm and BOP, radiographic bone loss (≥ 1/3 of the root length)	49 ± 13 (30-75)	52 ± 12 (30-75)	9/8	7/9	NS
**Chlorhexidine xanthan gel**												
(Jain et al. 2013)	India	split-mouth	single	university	NR	chronic localized or generalized periodontitis	PPD 5-7 mm	NR (30-60)		17/13		NS
(Kranti et al. 2010)	India	split-mouth	single	university	NR	chronic periodontitis	PPD 5-8 mm	NR (25-65)		NR		NS
(Matesanz et al. 2013)	Spain	parallel	single	university	commercial	generalized chronic periodontitis	PPD > 4 mm and BOP	50.0 ± 8.3 (36–59)	50.2 ± 9.6 (36–71)	5/5	3/9	5/22 (22.7)
(Paolantonio et al. 2009)	Italy	split-mouth	4	university	NR	moderate to advanced periodontitis	Interproximal sites with PPD ≥ 5 mm and BOP	NR (24-58)		39/59		NS
**Doxycycline hyclate gel**												
(Ağan et al. 2006A)	Turkey	split-mouth	single	university	NR	chronic periodontitis	Nonadjacent single-rooted sites with PPD ≥ 6 mm, CAL ≥ 3 mm and BOP	55 (41-49)		6/4		NR
(Ağan et al. 2006B)						aggressive periodontitis		25 (19-31)		4/4		
(Deo et al. 2011)	India	parallel	single	university	NR	generalized chronic periodontitis	PPD 5-7 mm	36.8 ± 4.87 (30-45)		25/35		NS
(Machion et al. 2004)	Brazil	parallel	single	university	research fund	chronic periodontitis	Anterior sites with PPD ≥ 5 mm and BOP	40.45 ± 4.47 (NR)	42.00 ± 4.38 (NR)	19/24		43/43 (100)

(Tomasi et al. 2008)	Sweden	parallel	single	university	research fund	moderate to advanced periodontitis	PPD ≥ 5 mm and BOP	52 (37-67)	54 (32-70)	9/10	6/7	15/32 (46.9)
**Metronidazole gel**												
(Buduneli et al. 2001)	Turkey	split-mouth	single	university	commercial	chronic periodontitis	Nonadjacent sites with PPD ≥ 5 mm and CAL ≥ 4 mm	41.1 ± 4.9 (35-50)		8/10		NR
(Griffiths et al. 2000)	UK	split-mouth	2	university	commercial	moderate to advanced chronic periodontitis	PPD ≥ 5 mm	46 (34–63)		18/27		15/45 (33.3)
								47 (34–71)		24/19		19/43 (44.2)
(Leiknes et al. 2007)	Norway	split-mouth	single	university	research fund	chronic periodontitis	Proximal sites with PPD ≥ 5 mm and BOP	50.3 (NR)		10/11		NR
(Stelzel and Florès-de-Jacoby 2000)	Germany	split-mouth	single	university	commercial	chronic periodontitis	PPD ≥ 5 mm	47 (23–70)		35/24		25/59 (42.4)
**Minocycline gel/ointment**												
(Jain et al. 2012)	India	split-mouth	single	university	NR	moderate to severe chronic periodontitis	PPD > 5 mm and CAL ≥ 4 mm	NR		NR		NS
(Soeroso et al. 2016)	Indonesia	parallel	single	university	commercial	localized chronic periodontitis	Interproximal sites with PPD 4-6 mm, CAL ≥ 4 mm and BOP	43.67 ± 6.88 (30-55)	44.00 ± 6.93 (30-55)	7/33	9/31	NS
(Zhao et al. 2006)	China	parallel	single	university	NR	moderate to advanced periodontitis	PPD ≥ 5 mm and BOP	49.46 (38-71)		64/0		64/64 (100)
**Minocycline microspheres**												

(Aboelsaad et al. 2014)	Lebanon	split-mouth	single	university	NR	moderate to severe chronic periodontitis	CAL ≥ 4 mm and PPD ≥ 5 mm	37 (31–49)		5/15		20/20 (100)
(Henderson et al. 2002)	New Zealand	split-mouth	single	university	commercial	moderate to severe chronic periodontitis	Interproximal sites with PPD 6-9 mm	46.3 (35-69)		7/8		6/15 (40)
(Van Dyke et al. 2002)	USA	parallel	single	university	NR	moderate to severe periodontitis	PPD ≥ 6 mm	NR		NR		NR
*(103A 2000)	USA	parallel	8	NR	commercial	moderate to advanced chronic periodontitis	PPD 6-9 mm and BOP	48.6 ± 10.1 (29-76)	SRP alone: 48 ± 10 (31-76) Vehicle: 47.7 ± 9.7 (29-77)	69/52	SRP alone: 67/57 Vehicle: 67/56	151/368 (42.2)
*(103B 2000)	USA	parallel	8	NR	commercial	moderate to advanced chronic periodontitis	PPD 6-9 mm and BOP	49.6 ± 10.2 (29-75)	SRP alone: 47.7 ± 9.5 (29-72) Vehicle: 46.7 ± 10.3 (29-79)	65/63	SRP alone: 65/61 Vehicle: 77/49	120/380 (31.6)
**Tetracycline fibers**												
(Reddy et al. 2016)	India	parallel	single	university	NR	chronic periodontitis	Nonadjacent sites with PPD ≥ 5 mm and BOP	NR		NR		NS
**Hyaluronic acid gel**												

(Eick et al. 2013)	Germany	parallel	single	universit y	commercial	moderate or severe chronic periodontitis	PPD ≥ 5 mm	54.82 ± 9.35 (42-70)	54.06 ± 9.81 (41-72)	8/9	6/11	NS
**Sulfonic/sulfuric acid gel**												
(Isola et al. 2018)	Italy	split-mouth	single	universit y	research fund	generalized chronic periodontitis	PPD ≥ 5 mm and BOP, no furcations	46.7 (27-65)		19/17		NS
**Antimicrobial photodynamic therapy (aPDT)**												
**Indocyanine green (ICG)**												
(Gandhi et al. 2019)	USA and India	split-mouth	single	universit y	NR	moderate to severe chronic periodontitis	PPD ≥ 5 mm	NR (30-60)		NR		NS
(G. Hill et al. 2019)	Germany	split-mouth	single	universit y	commercial	chronic periodontitis	one single and one multi-rooted tooth, PPD ≥ 4 mm per quadrant	61.1 (NR)		3/17		NS
**Methylene blue (MB) 0.005%**												
(Berakdar et al. 2012)	Germany	split-mouth	single	universit y	NR	chronic periodontitis	Minimum 4 teeth with PPD ≥ 5 mm and BOP	59.3 ± 11.7 (38-74)		12/10		NS
(Müller Campanile et al. 2015)	Switzerland	split-mouth	single	private practice	commercial	chronic periodontitis	PPD > 4 mm, CAL > 1 mm and BOP	62.8 (37-77)		14/13		12/27 (44.4)
**Methylene blue (MB) 1%**												
(Betsy et al. 2014)	India	parallel	single	universit y	research fund	chronic periodontitis	Single rooted sites with PPD 4-6 mm	40.8 ± 8.3 (NR)	38.4 ± 9.6 (NR)	22/22	15/29	NS
(Dilsiz et al. 2013)	Turkey	split-mouth	single	universit y	research fund	chronic periodontitis	Nonadjacent sites, PPD ≥ 5 mm, BOP and	40.7 ± 7.3 (30-58)		10/14		NS

							radiographic bone loss					
(Luchesi et al. 2013)	Brazil	parallel	single	university	research fund	chronic periodontitis	one buccal or lingual class II furcation (Hamp et al. 1975) with PPD ≥ 5 mm and BOP	50.75 ± 8.18 (NR)	50.24 ± 10.89 (NR)	7/14	6/15	NS
(Malgikar et al. 2016)	India	split-mouth	single	university	NR	chronic periodontitis	PPD ≥ 5 mm, radiographic bone loss	NR (24-55) Male: 36.73 ± 8.46 Female: 34.33 ± 6.80		15/9		NS
**Phenothiazine chloride (PC)**												
(Alwaeli et al. 2015)	Jordan	split-mouth	single	university	NR	chronic periodontitis	Minimum 4 posterior teeth with CAL ≥ 4 mm	40.96 ± 13.34 (NR)		5/11		NR
(Chondros et al. 2009)	The Netherlands	parallel	single	university	commercial	chronic periodontitis	PPD ≥ 4 mm and BOP	50.6 ± 9.2 (NR)	48.3 ± 7.9 (NR)	5/7	5/7	7/24 (29.2)
(Christodoulides et al. 2008)	The Netherlands	parallel	single	university	commercial	chronic periodontitis	NR	43.7 ± 7.3 (NR)	47.3 ± 8.8 (NR)	5/7	6/6	3/24 (12.5)
**Toluidine blue O (TBO)**												
(Theodoro et al. 2012)	Brazil	split-mouth	single	university	research fund	chronic periodontitis	Nonadjacent sites with PPD 5-9 mm and BOP	43.12 ± 8.2 (NR)		12/21		NS
**Subgingival antiseptic irrigation**												
(Denez et al. 2016)	Belgium	split-mouth	single	university	research fund	moderate to severe chronic periodontitis	PPD ≥ 4 mm and BOP, radiographic bone loss	45 ± 9.7 (28-60)		10/18		NS

(do Vale et al. 2016)	Brazil	parallel	single	university	research fund	generalized aggressive periodontitis	PPD ≥ 5 mm and BOP	28.54 ±4.14 (NR)	28.57 ±4.59 (NR)	9/5	12/2	NS
(Krück et al. 2012)	Germany and Switzerland	parallel	single	university	research fund	generalized moderate chronic periodontitis	PPD 4-6 mm	51.82 ± 10.61 (35-68)<br>50.13 ± 9.74 (34-71)	51.00 ± 12.65 (34-75)	5/12<br>8/9	9/8	NR

NR—not reported; NS—nonsmokers; *103A and 103B are two separate studies with individual data reported under one document (FDA Center for Drug Application Reviews 2000).

Table S3. Clinical outcome assessment of included studies.

Study citation	Blinding	Examiners	Calibration	Probing type	Use of stent	Gingival bleeding indices	Plaque indices
(Azmak et al. 2002)	single	1	NR	Manual	NR	Saxer and Muhlemann 1975	Silness and Loe 1964
(Heasman et al. 2001)	single	2	Yes	Manual (PPD), Computer assisted (CAL)	NR	Muhlemann 1977	Silness and Loe 1964
(Paolantonio et al. 2008a)	single	4	Yes	Manual	yes	Lobene 1986	Loe 1967
(Paolantonio et al. 2008b)	single	1	NR	Manual	NR	NR	NR
(Reddy et al. 2016)	NR	1	NR	Manual	NR	NR	NR
(Sakellari et al. 2010)	single	1	Yes	Manual	NR	NR	NR
(Cosyn et al. 2007)	single	1	yes	Manual	NR	Muhlemann and Son 1971	Quigley and Hein 1962
(M. Jain et al. 2013)	single	1	NR	Manual	yes	Muhlemann and Son 1971, Loe and Silness 1963	Silness and Loe 1964
(Kranti et al. 2010)	triple	1	NR	Manual	yes	Loe 1967	Loe 1967
(Matesanz et al. 2013)	triple	2	Yes	Computer assisted	NR	Loe 1967	Loe 1967
(Paolantonio et al. 2009)	single	3	Yes	Manual	yes	Loe 1967	Lobene 1986
(Ağan et al. 2006)	single	1	NR	NR	NR	Saxer and Muhleman 1975	Quigley and Hein 1962
(Deo et al. 2011)	single	1	NR	Manual	NR	Muhlemann and Son 1971	Quigley and Hein 1962

(Machion et al. 2004)	single	1	Yes	Computer assisted	Yes	NR	NR
(Tomasi et al. 2008)	single	2	Yes	Manual	NR	NR	NR
(Buduneli et al. 2001)	single	1	NR	Manual	NR	Saxer and Muhleman 1975	Ainamo and Bay 1975
(Griffiths et al. 2000)	single	2	Yes	Manual	Yes	Muhleman 1977	NR
(Leiknes et al. 2007)	single	1	NR	Computer assisted	NR	NR	NR
(Stelzel and Florès-de-Jacoby 2000)	single	NR	NR	Manual	NR	NR	NR
(Jain et al. 2012)	NR	NR	NR	NR	Yes	Silness and Loe 1964	Ainamo and Bay 1975
(Soeroso et al. 2016)	single	NR	NR	Manual	NR	Muhlemann and Son 1971	Loe and Silness 1967
(Zhao et al. 2006)	single	1	NR	NR	NR	NR	NR
(Aboelsaad et al. 2014)	single	1	Yes	Manual	NR	NR	Silness and Loe 1964
(Henderson et al. 2002)	single	1	NR	Manual	NR	Muhlemann 1977	Silness and Loe 1964
(Van Dyke et al. 2002)	double	1	NR	Computer assisted	NR	Loe and Silness 1963	Silness and Loe 1964
(103A 2000)*	single	8	Yes	Manual	NR	NR	NR
(103B 2000)*	single	8	Yes	Manual	NR	NR	NR
(Eick et al. 2013)	Non	1	NR	Manual	NR	NR	Lange 1977
(Isola et al. 2018)	single	1	yes	Manual	NR	Muhlemann and Son 1971	Quigley and Hein 1962
(Gandhi et al. 2019)	double	1	NR	Manual	NR	Loe 1967	Loe 1967
(G. Hill et al. 2019)	single	1	NR	Force controlled	NR	NR	NR
(Berakdar et al. 2012)	single	1	NR	Manual	NR	Loe and Silness 1963	Silness and Loe 1964
(Müller Campanile et al. 2015)	single	1	NR	NR	NR	Loe and Silness 1963	Silness and Loe 1964
(Betsy et al. 2014)	single	2	Yes	Manual	NR	Ainamo and Bay 1975	Silness and Loe 1964
(Dilsiz et al. 2013)	double	1	Yes	Computer assisted	Yes	Loe 1967	Loe 1967
(Luchesi et al. 2013)	double	1	Yes	Manual	Yes	Muhlemann and Son 1971	Ainamo and Bay 1975
(Malgikar et al. 2016)	triple	1	Yes	Manual	Yes	Loe and Silness 1963	Silness and Loe 1964

(Alwaeli et al. 2015)	single	2	Yes	Force controlled	yes	NR	NR
(Chondros et al. 2009)	Single	1	Yes	Manual	NR	NR	O'Leary 1972
(Christodoulides et al. 2008)	single	1	Yes	Manual	NR	NR	O'Leary 1972
(Theodoro et al. 2012)	single	1	Yes	Manual	Yes	NR	NR
(Denez et al. 2016)	single	1	yes	Manual	Yes	Loe 1967	Silness and Loe 1964
(do Vale et al. 2016)	single	1	yes	Manual	Yes	Muhlemann and Son 1971	Ainamo and Bay 1975
(Krück et al. 2012)	Non	3	yes	Manual	NR	NR	NR

NR—not reported; *additional data from Williams et al. (2001).

Table S4. Intervention characteristics of included studies.

Study citation	Status of perio-dontitis	Perio-dontal therapy pre-inter-vention	Perio-dontal therapy post-interv-ention	Area of inter-vention FM/PM	Test group				Control group		
					Inter-vention	Frequency of appli-cation	Dressing	Appli-cation time	Inter-vention	Operator	Debride-ment time
**Chlorhexidine chip**											
(Azmak et al. 2002)	Untreated	> 6 m prior to BL	FMSP and OHI	PM – 1 site	Periochip® + SRP	single	NR	NR	SRP	NR	5 min/tooth
(Heasman et al. 2001)	Recurrent	SPT > 3 m	NR	FM - PPD ≥ 5 mm and BOP	Periochip® + SRP	single	NR	NR	SRP	Dental hygienist (SRP), Dentist (chip)	5 min/tooth
(Paolantonio et al. 2008a)	Untreated	> 6 m prior to BL	FMSP and OHI	PM – 1 site	Periochip® + SRP	single	NR	NR	SRP	NR	5 min/site; 2 h/session
(Paolantonio et al. 2008b)	Untreated	> 6 m prior to BL	FMSP and OHI	PM – 1 site	Periochip® + SRP	single	NR	NR	SRP	NR	5 min/site; 2 h/session
(Reddy et al. 2016)	Untreated	> 3 m prior to BL	FMSP and OHI	PM—1 site	PerioCol®-CG + SRP	single	NR	NR	SRP	NR	NR
(Sakellari et al. 2010)	Untreated	> 12 m prior to BL	OHI	PM—4 sites	Periochip® + SRP	single	NR	NR	SRP	NR	5-10 min/tooth
**Chlorhexidine varnish**											
(Cosyn et al. 2007)	Untreated	NR	NR	FM	EC40® + SRP	single	NR	NR	SRP	NR	3 h/patient

**Chlorhexidine xanthan gel**											
(M. Jain et al. 2013)	Untreated	> 6 m prior to BL	NR	PM—1 site	Chlo-Site® + SRP	single	Yes	NR	SRP	NR	No time restriction
(Kranti et al. 2010)	Untreated	> 6 m prior to BL	FMSP and OHI	FM—PPD 5-8 mm	Chlo-Site® + SRP	single	NR	NR	Placebo + SRP	NR	NR
(Matesanz et al. 2013)	Recurrent	> 6 m prior to BL or SPT > 1 year	FMSP and OHI	PM—4-10 sites	Chlo-Site® + SRP	single	Yes	NR	Placebo + SRP	NR	NR
(Paolantonio et al. 2009)	Untreated	> 6 m prior to BL	FMSP and OHI	PM—1 site	Chlo-Site® + SRP	single	NR	NR	SRP	NR	5 min/site, 2 h/session
**Doxycycline hyclate gel**											
(Ağan et al. 2006)	Untreated	> 6 m prior to BL	FMSP and OHI	PM—2 sites	Atridox® + SRP	single	Yes	NR	SRP	NR	10 min/tooth
(Deo et al. 2011)	Untreated	NR	OHI	PM—1 site	Atridox® + SRP	single	NR	NR	Placebo + SRP	NR	NR
(Machion et al. 2004)	Untreated	> 6 m prior to BL	FMSP and OHI	FM—PPD ≥ 5 mm	Atridox® + SRP	single	NR	NR	SRP	Experienced	No time restriction
(Tomasi et al. 2008)	Untreated	> 12 m prior to BL	FMSP and OHI	FM—PPD ≥ 5 mm	Atridox® + U/S	single	No	NR	U/S	Dental hygienist	No time restriction
**Metronidazole gel**											
(Buduneli et al. 2001)	Untreated	> 6 m prior to BL	OHI	PM—2-3 sites	Elyzol® + SRP	2x (BL, 1 w)	NR	NR	SRP	NR	9-12 min/tooth
(Griffiths et al. 2000)	Untreated	> 6 m prior to BL	FMSP and OHI	FM—PPD ≥ 5 mm	Elyzol® + SRP	2x (BL, 1 w)	NR	NR	SRP	NR	Max 60 min/arch
(Leiknes et al. 2007)	Recurrent	SPT ≥ 2 y	OHI	PM—1 site	Elyzol® + SRP	2x (BL, 1 w)	NR	NR	SRP	NR	No time restriction
(Stelzel and Florès-de-Jacoby 2000)	Untreated, recurrent	> 3 m prior to BL	NR	FM—PPD ≥ 5 mm	Elyzol® + SRP	2x (BL, 1 w)	NR	NR	SRP	NR	NR
**Minocycline gel/ointment**											
(Jain et al. 2012)	Untreated	NR	FMSP	PM—22 sites	Dentomycin® + SRP	3x (BL, 2 w, 4 w) [#]	Cyano-acrylate	NR	SRP	NR	NR
(Soeroso et al. 2016)	Untreated	> 6 m prior to BL	NR	FM	Periocline® + SRP	4x (BL, 1 w, 2 w, 3 w) [#]	NR	NR	SRP	NR	NR
(Zhao et al. 2006)	Untreated	Never	FMSP + OHI	FM—PPD ≥ 5 mm	Periocline® + SRP	4x (BL, 1 w, 2 w, 3 w) [#]	NR	NR	SRP	NR	10 min/tooth
**Minocycline microspheres**											

(Aboelsaad et al. 2014)	Untreated	> 6 m prior to BL	FMSP and OHI	FM—PPD ≥ 5 mm	Arestin®+ SRP	single	NR	NR	SRP	NR	NR
(Henderson et al. 2002)	Untreated	> 6 m prior to BL	NR	PM—2 sites	Arestin®+ SRP	single	No	NR	SRP	Dentist	90 min/patient
(Van Dyke et al. 2002)	Untreated	NR	NR	PM—2 teeth	Arestin®+ SRP	single	NR	NR	SRP	NR	NR
(103A, 103B 2000)*	Untreated	> 6 m prior to BL	NR	FM—PPD ≥ 5 mm	Arestin®+ SRP	single	NR	NR	SRP, Placebo + SRP	NR	No time restriction
**Tetracycline fibers**											
(Reddy et al. 2016)	Untreated	> 3 m prior to BL	FMSP and OHI	PM—1 site	PerioCol®-TC + SRP	single	NR	NR	SRP	NR	NR
**Hyaluronic acid gel**											
(Eick et al. 2013)	Untreated	> 12 m prior to BL	FMSP + OHI	FM	0.8% Gengigel® + SRP	single	NR	NR	SRP	Periodontist	NR
**Sulfonic/sulfuric acid gel**											
(Isola et al. 2018)	Untreated	> 12 m prior to BL	FMSP + OHI	PM—1 quadrant	HybenX® + SRP	single	NR	60 s	NaCL+ SRP	Periodontist	9 min/ quadrant
**Antimicrobial photodynamic therapy**											
(Gandhi et al. 2019)	Untreated	> 6 m prior to BL	FMSP and OHI	FM—PPD ≥ 5 mm	ICG + SRP	single	Laser: 100 mW and 810 nm	2 min	SRP	Periodontist	5 min/tooth
(G. Hill et al. 2019)	Untreated	> 6 m prior to BL	FMSP and OHI	PM—2 quadrants PPD ≥ 4 mm	perio green® + SRP	single	Laser: 100 mW and 808 nm	NR	SRP	NR	NR
(Berakdar et al. 2012)	Untreated	NR	FMSP	PM—2 teeth	PeriowaveTM + SRP	single	Laser: 75 mW and 670 nm	1 min	SRP	NR	NR
(Müller Campanile et al. 2015)	Recurrent	SRP > 3-12 m	OHI	PM—4 sites	PeriowaveTM + U/S	single	Laser: 280 mW and 670 nm	1 min	Sham + U/S	NR	NR
(Betsy et al. 2014)	Untreated	NR	OHI	FM—PPD 4-6 mm	MB 1% + SRP	single	Laser: 1 W and	3 min	SRP	Experienced periodontist	No time restriction

							655 nm				
(Dilsiz et al. 2013)	Untreated	> 6 m prior to BL	FMSP and OHI	FM—PPD ≥ 5 mm	MB 1% + SRP	single	Laser: 100 mW and 808 nm	3 min	Sham + SRP	NR	No time restriction
(Luchesi et al. 2013)	Untreated	> 6 m prior to BL	FMSP	PM—1 site	MB 1% + SRP	single	Laser: 60 mW and 660 nm	1 min	Sham + SRP	NR	NR
(Malgikar et al. 2016)	Untreated	> 6 m prior to BL	OHI	PM—>1 site	MB 1% + SRP	single	Laser: 1 W and 980 nm	3 min	SRP	NR	NR
(Alwaeli et al. 2015)	Untreated	None	FMSP and OHI	PM—2 quadrants PPD ≥ 4 mm	HELBO® + SRP	single	Laser: 100 mW and 660 nm	1-3 min	SRP	Dentist	NR
(Chondros et al. 2009)	Recurrent	SRP > 6 m	NR	FM—PPD ≥ 4 mm	HELBO® + SRP	single	Laser: 75 mW and 670 nm	1 min	SRP	Dental therapist	No time restriction
(Christodoulides et al. 2008)	Untreated	> 2 y prior to BL	NR	FM	HELBO® + SRP	single	Laser: 75 mW and 670 nm	1 min	SRP	Experienced	No time restriction
(Theodoro et al. 2012)	Untreated	> 12 m prior to BL	FMSP and OHI	PM—1 site	TBO 0.01% + SRP	Single	Laser: 30 mW and 660 nm	1 min	SRP, Sham + SRP	NR	NR
**Subgingival antiseptic irrigation**											
(Denez et al. 2016)	Untreated	> 3 m prior to BL	OHI	PM—4 sites	10% PVP-I + SRP	single	NR	60 s	0.9% NaCl + SRP	Periodontist	1.5 h/patient
(do Vale et al. 2016)	Untreated	> 6 m prior to BL	FMSP and OHI	FM	10% PVP-I + U/S	Single	NR	NR	U/S	NR	45 min/session
(Krück et al. 2012)	Untreated	NR	NR	FM	0.12% CHX + SRP	single	NR	NR	0.9% NaCl + SRP	NR	5 min/tooth
					7.5% PVP-I + SRP	single	NR				

NR—not reported; FM—full mouth; PM—partial mouth; BL—baseline; SPT—supportive periodontal therapy; FMSP—full mouth supragingival prophylaxis; *additional data from Williams et al. (2001), [#]considered as single application per manufacturer's instruction.

Table S5. Outcomes data of included studies.

Study citation	Follow-up duration (months)	Test group					Control group					
		Sample size BL/end of study	Mean PPD reduction	Mean CAL gain	Mean BOP reduction	Adverse events	Sample size BL/end of study	Mean PPD reduction	Mean CAL gain	Mean BOP reduction	Adverse events	Statistical notes
**Chlorhexidine chip**												
(Azmak et al. 2002)	6	22/20	2.40 ± 0.63	1.70 ± 0.56	NR	NR	22/20	2.10 ± 0.63	1.60 ± 0.56	NR	NR	End data in graphs
(Heasman et al. 2001)	6	26/24	0.78 ± 0.61	0.43 ± 0.76	1.08 ± 0.51	1 non-intervention related aphthae of the buccal mucosa	26/24	0.45 ± 0.66	0.15 ± 0.46	0.59 ± 0.51	NR	SE converted to SD, ITT
(Paolantonio et al. 2008a)	6	116/116	1.50 ± 1.08	1.10 ± 1.08	NR	NR	116/116	0.90 ± 1.08	0.50 ± 1.08	NR	NR	SE converted to SD
(Paolantonio et al. 2008b)	6	82/82	1.50 ± 0.91	1.10 ± 0.91	86%	NR	82/82	0.90 ± 0.91	0.50 ± 0.91	85%	NR	Assumed SE converted to SD
(Reddy et al. 2016)	12	16/16	3.56 ± 1.26	2.13 ± 1.14	NR	NR	16/16	2.94 ± 1.01	0.88 ± 1.06	NR	NR	SE converted to SD
(Sakellari et al. 2010)	6	27/25	1.79 ± 1.02	1.40 ± 1.19	0.25 ± 0.41	None	29/25	2.05 ± 0.90	1.40 ± 1.48	0.33 ± 0.39	None	Mean difference NR
**Chlorhexidine varnish**												
(Cosyn et al. 2007)	6	17/15	1.13 ± 0.71	0.36 ± 1.14	0.56 ± 0.42	NR	16/14	0.96 ± 0.53	0.39 ± 0.95	0.48 ± 0.52	NR	
**Chlorhexidine xanthan gel**												
(M. Jain et al. 2013)	6	30/30	2.80 ± 0.73	1.67 ± 3.55	1.56 ± 0.78	NR	30/30	2.20 ± 0.92	2.23 ± 2.67	1.44 ± 0.96	NR	Mean difference NR
(Kranti et al. 2010)	6	10/10	3.11 ± 0.47	3.11 ± 0.65	0.93 ± 0.21	NR	10/10	2.44 ± 0.55	2.44 ± 0.98	0.62 ± 0.25	NR	
(Matesanz et al. 2013)	6	10/10	0.34 ± 0.41	0.23 ± 0.70	0.14 ± 0.19	None	12/11	0.20 ± 0.40	0.04 ± 0.70	0.17 ± 0.17	None	SE, CI converted to SD

(Paolantonio et al. 2009)	6	98/98	2.33 ± 0.99	1.40 ± 0.99	NR	NR	98/98	1.50 ± 0.99	0.50 ± 0.99	NR	NR	End data in graphs
**Doxycycline hyclate gel**												
(Ağan et al. 2006A)	6	10/10	3.00	1.56	NR	None	10/10	3.06	1.44	NR	None	No SD
(Ağan et al. 2006B)		8/8	3.11	1.55	NR		8/8	2.75	1.40	NR		
(Deo et al. 2011)	6	30/30	3.03 ± 0.92	2.00 ± 0.64	NR	None	30/30	2.30 ± 0.65	1.13 ± 1.07	NR	None	
(Machion et al. 2004)	6	NR/22	2.17 ± 0.93	1.63 ± 0.93	37 ± 30	None	NR/21	1.76 ± 0.63	1.04 ± 0.71	36 ± 33	NR	
(Tomasi et al. 2008)	9	NR/19	1.10 ± 0.41	0.80 ± 0.52	26% ± 38.6%	4—painkillers, 4—chewing discomfort due to root sensitivity	NR/13	1.10 ± 0.50	0.90 ± 0.66	31% ± 37%	4—chewing discomfort due to root sensitivity	CI converted to SD
**Metronidazole gel**												
(Buduneli et al. 2001)	12	18/18	3.20 ± 0.82	2.06 ± 0.59	NR	NR	18/18	3.41 ± 0.74	2.12 ± 0.53	NR	NR	
(Griffiths et al. 2000)	9	88/84	1.50 ± 3.89	0.80 ± 3.89	35%	NR	88/84	1.00 ± 3.89	0.40 ± 3.89	26%	NR	SE provided, converted to SD
(Leiknes et al. 2007)	6	21/21	1.90 ± 1.26	1.60 ± 1.72	38%	NR	21/21	1.80 ± 0.92	1.00 ± 2.02	33%	NR	
(Stelzel and Florès-de-Jacoby 2000)	9	64/59	1.37 ± 0.85	1.01 ± 1.29	36% ± 31.5%	None, bitter taste in ½ of patients	64/59	1.19 ± 0.97	0.94 ± 1.39	28% ± 35.2%	None	
**Minocycline gel/ointment**												
(Jain et al. 2012)	9	15/13	2.36 ± 1.04	2.14 ± 1.74	0.363	None	15/13	1.39 ± 1.02	1.62 ± 1.80	0.116	None	ITT
(Soeroso et al. 2016)	6	42/40	1.79 ± 0.65	1.79 ± 0.65	0.34 ± 0.41	NR	42/42	1.87 ± 0.84	0.73 ± 1.79	0.48 ± 0.41	NR	Mean differences NR
(Zhao et al. 2006)	6	32/29	2.11 ± 0.57	1.90 ± 0.63	55%	NR	32/29	1.44 ± 0.58	1.21 ± 0.57	59%	NR	
**Minocycline microspheres**												

(Aboelsaad et al. 2014)	6	20/20	1.88 ± 0.56	1.10 ± 0.22	19%	None	20/20	0.95 ± 0.46	0.45 ± 0.71	18%	None	
(Henderson et al. 2002)	6	15/15	2.50 ± 1.40	2.10 ± 1.50	47%	NR	15/15	1.70 ± 1.58	1.35 ± 1.33	43%	NR	Adjacent and remote sites combined and averaged
(Van Dyke et al. 2002)	6	12/12	1.94 ± 0.73	1.02 ± 0.90	NR	2—black hairy tongue and abscess formation	12/10	1.66 ± 0.69	0.54 ± 0.62	NR	None	Reported in SE, converted to SD
(103A 2000)*	9	121/115	1.2 ± 0.79	1.01 ± 0.82	26% ± 25.29%	594—periodontitis, tooth sensitivity, tooth caries, infection, dental pain, gingivitis, headache, stomatitis, flu syndrome, dental infection, accidental injury	124/114	1.04 ± 0.81	0.98 ± 0.88	27% ± 27.47%	SRP alone—543; vehicle—589; same AE as test	ITT
							123/112	0.9 ± 0.7	0.87 ± 0.87	19% ± 22.33%		
(103B 2000)*	9	128/124	1.63 ± 0.8	1.09 ± 1.03	32% ± 27.21%		126/115	1.32 ± 0.8	0.99 ± 1.01	28% ± 27.58%		
							126/118	1.3 ± 0.81	0.92 ± 0.94	23% ± 27.5%		
**Tetracycline fibers**												
(Reddy et al. 2016)	12	16/16	3.00 ± 1.14	1.13 ± 1.11	NR	NR	16/16	2.94 ± 1.01	0.88 ± 1.06	NR	NR	SE converted to SD
**Hyaluronic acid gel**												
(Eick et al. 2013)	6	21/17	1.07 ± 0.36	1.24 ± 0.58	7.5% ± 24.73%	None	21/17	0.82 ± 0.36	1.34 ± 0.57	5.2% ± 19.33%	None	
**Sulfonic/sulfuric acid gel**												
(Isola et al. 2018)	12	17/15	2.73 ± 0.65	1.94 ± 0.33	64% ± 3.4%	None	16/14	1.8 ± 0.52	0.74 ± 0.42	35% ± 4.2%	None	
**Antimicrobial photodynamic therapy**												
(Gandhi et al. 2019)	6	30/26	1.59 ± 1.07	1.46 ± 1.17	0.96	NR	30/26	0.52 ± 0.52	0.55 ± 0.56	0.73	NR	
(G. Hill et al. 2019)	9	20/20	1.16 ± 1.11	0.93 ± 1.95	20%	None	20/20	1.16 ± 1.08	0.95 ± 1.77	18%	None	4-6 mm subgroup data used, higher n

(Berakdar et al. 2012)	6	22/22	2.9 ± 0.8	2.50	86%	NR	22/22	2.4 ± 0.6	2.00	77%	NR	No SD or SE provided for CAL
(Müller Campanile et al. 2015)	6	28/27	3.40 ± 1.94	3.70 ± 3.10	48%	2—pain/discomfort	28/27	2.90 ± 1.84	3.00 ± 2.58	19%	None	Mean difference NR, repeated arm removed
(Betsy et al. 2014)	6	50/44	2.40 ± 0.86	2.30 ± 1.36	75%	None	50/44	1.40 ± 0.90	1.53 ± 1.50	25%	None	Median, IQR reported, converted to mean, SD, ITT
(Dilsiz et al. 2013)	6	24/24	1.54 ± 0.59	1.54 ± 1.10	50% ± 0.52%	None	24/24	1.42 ± 0.88	1.50 ± 0.88	46% ± 0.52%	None	KTP arm is removed (not of interest)
(Luchesi et al. 2013)	6	21/16	1.59 ± 1.11	0.78 ± 1.54	63%	None	21/21	1.50 ± 1.73	1.00 ± 1.69	55%	None	
(Malgikar et al. 2016)	6	24/24	2.57 ± 0.53	2.55 ± 0.44	1.79 ± 0.49	None	24/24	2.50 ± 0.54	2.63 ± 0.47	1.78 ± 0.37	None	
(Alwaeli et al. 2015)	12	21/16	1.51 ± 1.54	1.48 ± 1.89	64%	None	21/16	0.6 ± 1.66	0.13 ± 1.7	13%	None	
(Chondros et al. 2009)	6	12/12	0.8 ± 0.5	0.7 ± 0.7	50% ± 29.6%	None	12/12	0.9 ± 0.8	0.5 ± 0.6	10% ± 42.9%	None	
(Christodoulides et al. 2008)	6	12/12	0.9 ± 0.3	0.7 ± 0.3	44% ± 15.5%	None	12/12	0.7 ± 0.7	0.5 ± 0.5	39% ± 20.4%	None	
(Theodoro et al. 2012)	6	33/33	2.33 ± 1.60	1.56 ± 2.56	48%	None	33/33	2.71 ± 1.55	1.98 ± 2.13	70%	None	
							33/33	3.40 ± 1.32	1.99 ± 2.41	64%		
**Subgingival antiseptic irrigation**												
(Denez et al. 2016)	6	28/20	1.90 ± 1.34	1.95 ± 0.94	1.30 ± 0.36	2—NR	28/20	1.92 ± 0.54	1.93 ± 0.22	1.53 ± 0.13	2 - NR	Assumed SE, converted to SD

(do Vale et al. 2016)	6	17/14	1.97 ± 0.52	0.73 ± 1.07	NR	NR	17/14	2.05 ± 0.68	1.18 ± 1.05	NR	NR	
(Krück et al. 2012)	12	17/17	1.23 ± 0.61	1.28 ± 0.91	27% ± 20%	None	17/17	1.25 ± 0.56	1.15 ± 1.51	21% ± 27%	None	Mean difference NR
		17/17	1.39 ± 0.52	1.48 ± 0.88	28% ± 29%							

NR—not reported; ITT—intention to treat; *additional data from Williams et al. (2001).

S2. Sensitivity analyses

Studies with certain design parameters were removed to check for bias that could affect the effect in several sensitivity analyses.

**Sensitivity analyses**	**Definition**
High risk of bias	Studies at high risk of bias were excluded. High of bias was defined using the criteria in the Cochrane Collaboration's Risk of Bias tool (RoB 2.0) (Sterne et al. 2019) and overall risk of bias determined using the provided Excel worksheet (RoB2 Development Group 2019).
Imputed standard deviation	In studies whereby standard deviations were not provided by the study authors, the values were re-calculated using appropriate formulas (Higgins and Green 2011; Wan et al. 2014). These studies with imputed standard deviations were excluded.
Placebo	The possible influence of placebo controls was examined using two sensitivity analyses:<br>a) Head-to-head studies<br>Entire placebo-controlled studies were excluded even though studies had two or more active arms.<br>b) Only active arms<br>Placebo arms from selected studies were excluded, but the remaining active arms of the studies were kept.
Study design	Studies with split-mouth or parallel groups were excluded. The design with the most studies were kept as comparator.
Duration	Studies with a duration of less than twelve months were excluded.

Below, the results from the changes in heterogeneity in each sensitivity analysis are presented.

Table S6. Sensitivity analyses of specific parameters.

**Sensitivity analysis**									
**Including only studies with**	**PPD**				**CAL**				**n studies included**
	τ	**% of variance (τ^2)**	**I^2 (%)**	***p*-value**	τ	**% of variance (τ^2)**	**I^2 (%)**	***p*-value**	
Unadjusted	0.27	-	66.4	< 0.0001	0.22	-	48.4	0.00	45
Low and some concerns of overall risk of bias	0.29	11.61	67.2	< 0.0001	0.19	-26.72	37.6	0.05	29
Original standard deviations	0.27	-4.50	66.4	< 0.0001	0.22	-2.27	48.8	0.00	41
Active arms (no placebo control)	0.25	-13.59	59.7	0.00	0.21	-11.70	42.9	0.02	35
Excluding placebo arms from three arm trials	0.27	-0.73	65.8	< 0.0001	0.24	15.75	52.5	0.00	38
Split mouth design	**0.05**	**-96.24**	**6.6**	**0.38**	**0.12**	**-71.80**	**21.1**	**0.22**	26
Duration of less than 12 months	0.27	1.03	68	< 0.0001	0.21	-12.05	46.1	0.00	40

As it can be seen from the table above, removing the parallel groups from the analysis had the most important impact on the results (96% and 72% reduction of the variance). Therefore, the analysis is divided into split-mouth and parallel design with high risk of bias studies removed.

Table S7. Comparison of probing pocket depth (PPD) mean differences (95% CI), P-score and ranks from complete analysis and having removed studies at high risk of bias (ROB).

Intervention	All studies (45 trials)			Studies without high ROB (29 trials)			Only split-mouth design without high ROB (16 trials)			Only parallel design without high ROB (13 trials)		
	**MD**	**95% CI**	**Rank (P-score)**	**MD**	**95% CI**	**Rank (P-score)**	**MD**	**95% CI**	**Rank (P-score)**	**MD**	**95% CI**	**Rank (P-score)**
SMD only	0		19 (0.22)	0		15 (0.21)	0		12 (0.16)	0		9 (0.24)
Placebo	-0.19	-0.45; 0.07	13 (0.40)	-0.21	-0.49; 0.08	10 (0.39)	-0.20	-0.65; 0.25	9 (0.34)	-0.17	-0.45; 0.11	6 (0.44)
aPDT ICG	-0.88	-1.45; -0.31	2 (0.90)	-0.88	-1.46; -0.29	2 (0.87)	-0.92	-1.38; -0.46	4 (0.82)		Not in network	
aPDT MB 0.005%	-0.56	-1.11; -0.00	4 (0.73)	-0.56	-1.13; 0.01	4 (0.69)	-0.54	-0.99; -0.10	6 (0.60)		Not in network	
aPDT MB 1%	-0.37	-0.72; -0.01	8 (0.59)	-0.37	-0.75; 0.004	8 (0.56)	-0.18	-0.54; 0.19	10 (0.32)	-0.69	-1.14; -0.24	2 (0.85)
aPDT PC	-0.21	-0.67; 0.25	11 (0.44)	-0.06	-0.59; 0.46	12 (0.30)		Not in network		-0.07	-0.49; 0.34	7 (0.34)
aPDT TBO	0.69	-0.17; 1.55	20 (0.04)	0.68	-0.20; 1.56	16 (0.04)	0.70	-0.11; 1.52	13 (0.01)		Not in network	
CHX 0.12%	0.02	-0.71; 0.75	17 (0.28)		Not in network			Not in network			Not in network	
CHX chip	-0.38	-0.65; -0.11	7 (0.61)	-0.16	-0.62; 0.30	11 (0.37)	-0.30	-0.77; 0.17	7 (0.42)	0.02	-0.52; 0.55	8 (0.28)
CHX varnish	-0.17	-0.87; 0.53	12 (0.41)		Not in network			Not in network			Not in network	

CHX xanthan	-0.66	-1.00; -0.32	3 (0.83)	-0.47	-0.96; 0.02	5 (0.63)	-0.60	-1.09; -0.11	5 (0.63)	-0.31	-0.87; 0.25	4 (0.57)
DH	-0.33	-0.64; -0.01	9 (0.55)	-0.44	-0.87; -0.01	6 (0.61)	-0.15	-0.57; 0.27	11 (0.30)	-0.90	-1.50; -0.30	**1 (0.93)**
HA	-0.25	-0.84; 0.34	10 (0.47)	Not in network			Not in network			Not in network		
MET	-0.08	-0.46; 0.29	15 (0.31)	-0.23	-0.90; 0.43	9 (0.44)	-0.21	-0.78; 0.35	8 (0.35)	Not in network		
MINO gel	-0.44	-0.83; -0.06	6 (0.66)	-0.78	-1.29; -0.26	3 (0.83)	-0.97	-1.69; -0.25	3 (0.82)	-0.67	-1.12; -0.22	3 (0.84)
MINO microspheres	-0.44	-0.73; -0.16	5 (0.67)	-0.42	-0.74; -0.11	7 (0.60)	-0.93	-1.35; -0.51	2 (0.83)	-0.26	-0.53; 0.01	5 (0.55)
PI 10%	0.05	-0.47; 0.57	18 (0.23)	0.08	-0.64; 0.80	14 (0.24)	Not in network			0.08	-0.48; 0.64	11 (0.23)
PI 7.5%	-0.14	-0.82; 0.54	14 (0.39)	Not in network			Not in network			Not in network		
SA	-1.12	-1.77; -0.48	**1 (0.96)**	-1.14	-1.81; -0.46	**1 (0.95)**	-1.13	-1.74; -0.53	**1 (0.91)**	Not in network		
TC	0.02	-0.94; 0.98	16 (0.31)	0.10	-0.88; 1.08	13 (0.26)	Not in network			0.14	-0.76; 1.03	10 (0.24)

Comparison of CAL mean differences (95% CI), P-score, and ranks from complete analysis and having removed studies at high ROB.

Intervention	All studies (45 trials)			Studies without high ROB (29 trials)			Only split-mouth design without high ROB (16 trials)			Only parallel design without high ROB (13 trials)		
	MD	95% CI	Rank	MD	95% CI	Rank	MD	95% CI	Rank (P-score)	MD	95% CI	Rank (P-score)

			(P-score)			(P-score)						
SMD only	0		16 (0.27)	0		12 (0.28)	0		9 (0.31)	0		9 (0.27)
Placebo	0.01	-0.24; 0.26	17 (0.27)	0.07	-0.18; 0.32	14 (0.23)	0.11	-0.35; 0.57	10 (0.24)	0.03	-0.22; 0.27	10 (0.24)
aPDT ICG	-0.84	-1.41; -0.27	2 (0.87)	-0.84	-1.38; -0.31	2 (0.88)	-0.86	-1.29; -0.44	2 (0.87)	Not in network		
aPDT MB 0.005%	-0.53	-1.05; -0.01	5 (0.71)	-0.56	-1.00; -0.04	4 (0.72)	-0.51	-0.85; -0.16	4 (0.68)	Not in network		
aPDT MB 1%	-0.09	-0.42; 0.24	13 (0.36)	-0.06	-0.37; 0.24	10 (0.36)	0.08	-0.14; 0.30	11 (0.24)	-0.39	-0.87; 0.09	4 (0.66)
aPDT PC	-0.33	-0.73; 0.06	8 (0.57)	-0.20	-0.59; 0.19	9 (0.48)	Not in network			-0.20	-0.53; 0.13	5 (0.51)
aPDT TBO	0.43	-0.77; 1.62	20 (0.17)	0.45	-0.73; 1.62	15 (0.17)	0.46	-0.68; 1.60	12 (0.16)	Not in network		
CHX 0.12%	-0.13	-1.33; 1.07	11 (0.42)	Not in network			Not in network			Not in network		
CHX chip	-0.42	-0.66; -0.19	6 (0.66)	-0.25	-0.63; 0.13	8 (0.53)	-0.10	-0.40; 0.20	8 (0.41)	-0.50	-1.10; 0.10	3 (0.73)
CHX varnish	0.03	-0.84; 0.90	14 (0.32)	Not in network			Not in network			Not in network		
CHX xanthan	-0.60	-0.97; -0.24	4 (0.77)	0.01	-0.65; 0.67	11 (0.32_	0.56	-0.83; 1.95	13 (0.16)	-0.16	-0.85; 0.53	6 (0.46)
DH	-0.33	-0.63; -0.04	7 (0.58)	-0.40	-0.78; -0.02	6 (0.65)	-0.14	-0.53; 0.26	7 (0.43)	-0.84	-1.40; -0.28	**1 (0.92)**

HA	0.10	-0.48; 0.68	18 (0.24)	Not in network			Not in network			Not in network		
MET	-0.10	-0.46; 0.25	12 (0.37)	-0.50	-1.26; 0.25	5 (0.68)	-0.50	-1.21; 0.20	5 (0.66)	Not in network		
MINO gel	-0.79	-1.19; -0.38	3 (0.86)	-0.67	-1.11; -0.22	3 (0.81)	-0.52	-1.62; 0.58	6 (0.64)	-0.69	-1.08; -0.30	2 (0.87)
MINO microspheres	-0.31	-0.56; -0.05	9 (0.56)	-0.25	-0.50; -0.01	7 (0.54)	-0.65	-0.95; -0.35	3 (0.76)	-0.13	-0.37; 0.11	7 (0.45)
PI 10%	0.12	-0.37; 0.61	19 (0.22)	0.45	-0.42; 1.32	16 (0.13)	Not in network			0.45	-0.37; 1.27	11 (0.10)
PI 7.5%	-0.33	-1.49; 0.83	10 (0.54)	Not in network			Not in network			Not in network		
SA	-1.19	-1.71; -0.67	**1 (0.97)**	-1.13	-1.60; -0.66	**1 (0.97)**	-1.09	-1.58; -0.61	**1 (0.95)**	Not in network		
TC	0.11	-0.79; 1.01	15 (0.27)	0.19	-0.70; 1.07	13 (0.25)	Not in network			0.07	-0.80; 0.95	8 (0.30)

S3. Sensitivity analyses of studies with imputed data

In studies whereby standard deviations were not provided by the study authors, the values were re-calculated using appropriate formulas (Higgins and Green 2011; Wan et al. 2014). These studies with imputed standard deviations were excluded.

Network meta-analysis forest plots without studies with imputed standard deviations

1) Without Ağan et al. (2006)

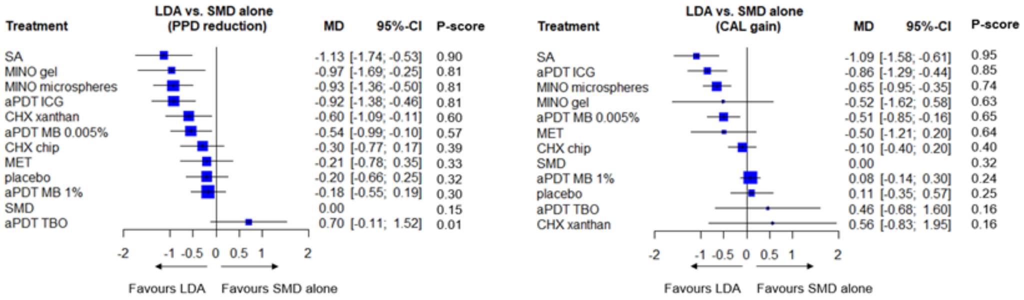

2) Without Betsy et al. (2014)

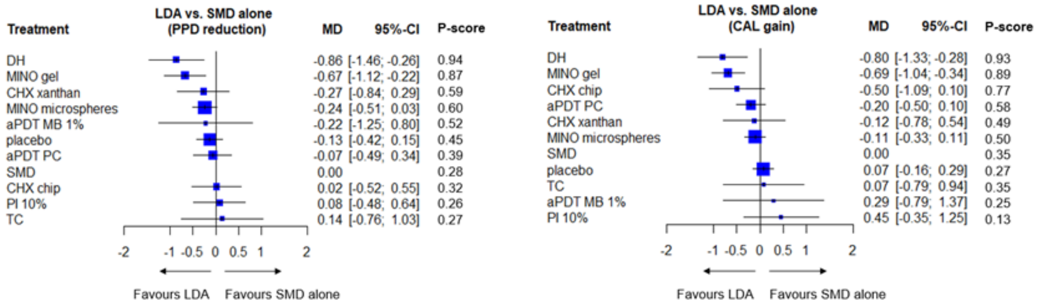

1. DH was removed from the split-mouth single application network. Still, the overall hierarchy was not affected for both PPD and CAL.

2. There was noted change in the hierarchy for aPDT MB 1% for PPD reduction (dropped to 5th rank from 2nd) with MD -0.22 mm (95% CI -1.25 to 0.80; P-score 0.52). The ranking hierarchy of the remaining LDA remained unchanged. Heterogeneity was increased to 51%. For CAL gain, aPDT MB 1% dropped from 4th to 10th rank with MD 0.28 mm (95% CI -0.79 to 1.37; P-score 0.25). However, the ranking hierarchy of the rest remained unchanged. Heterogeneity was reduced to 16.3%.

Figure S1. League tables.

For the league tables, treatments are arranged according to the rank of their overall effectiveness, with the direct (upper right half) and indirect (lower left half) comparisons from the highest to lowest ranked (left to right). Treatment estimates are stated as MD with 95% CIs. Pairwise comparisons that are statistically significant are highlighted in yellow. NA signifies that no direct comparisons were made between treatments. NA = not applicable.

League table of pairwise comparisons in the NMA for PPD changes with split-mouth design studies.

**SA**	NA	NA	NA	NA	NA	NA	NA	-0.93 (-1.33 to -0.53)	NA	NA	NA	NA
-0.20 (-0.94 to 0.53)	**MINO micro spheres**	NA	NA	NA	NA	NA	NA	NA	NA	NA	-0.93 (-1.35 to -0.51)	NA
-0.16 (-1.10 to 0.78)	0.04 (-0.80 to 0.88)	**MINO gel**	NA	NA	NA	NA	NA	NA	NA	NA	-0.97 (-1.69 to -0.25)	NA
-0.21 (-0.97 to 0.55)	-0.01 (-0.63 to 0.62)	-0.05 (-0.90 to 0.81)	**aPDT ICG**	NA	NA	NA	NA	NA	NA	NA	-0.92 (-1.38 to -0.46)	NA
-0.53 (-1.31 to 0.24)	-0.33 (-0.98 to 0.32)	-0.37 (-1.24 to 0.50)	-0.32 (-0.99 to 0.35)	**CHX xanthan**	NA	NA	NA	NA	NA	NA	-0.60 (-1.09 to -0.11)	NA
-0.59 (-1.28 to 0.10)	-0.39 (-1.00 to 0.23)	-0.43 (-1.27 to 0.42)	-0.38 (-1.02 to 0.26)	-0.06 (-0.72 to 0.60)	**aPDT MB 0.005%**	NA	NA	-0.50 (-1.43 to 0.43)	NA	NA	-0.50 (-0.99 to -0.01)	NA
-0.83 (-1.60 to -0.07)	-0.63 (-1.26 to 0.00)	-0.67 (-1.53 to 0.19)	-0.62 (-1.28 to 0.03)	-0.30 (-0.98 to 0.38)	-0.24 (-0.89 to 0.40)	**CHX chip**	NA	NA	NA	NA	-0.30 (-0.77 to 0.17)	NA
-0.92 (-1.75 to -0.10)	-0.72 (-1.42 to -0.01)	-0.76 (-1.68 to 0.16)	-0.71 (-1.44 to 0.02)	-0.39 (-1.13 to 0.36)	-0.33 (-1.05 to 0.39)	-0.09 (-0.82 to 0.64)	**MET**	NA	NA	NA	-0.21 (-0.78 to 0.35)	NA
-0.93 (-1.33 to -0.53)	-0.73 (-1.35 to -0.11)	-0.77 (-1.62 to 0.08)	-0.72 (-1.36 to -0.08)	-0.40 (-1.06 to 0.27)	-0.34 (-0.90 to 0.22)	-0.10 (-0.75 to 0.55)	-0.01 (-0.73 to 0.71)	**placebo**	0.12 (-0.37 to 0.61)	NA	-0.69 (-1.47 to 0.09)	-1.07 (-1.87 to -0.27)
-0.96 (-1.53 to -0.38)	-0.75 (-1.32 to -0.19)	-0.79 (-1.61 to 0.02)	-0.75 (-1.34 to -0.16)	-0.42 (-1.04 to 0.19)	-0.37 (-0.91 to 0.18)	-0.12 (-0.72 to 0.47)	-0.04 (-0.71 to 0.64)	-0.03 (-0.44 to 0.38)	**aPDT MB 1%**	NA	-0.07 (-0.49 to 0.35)	NA
-0.98 (-1.71 to -0.25)	-0.78 (-1.37 to -0.19)	-0.82 (-1.65 to 0.01)	-0.77 (-1.39 to -0.15)	-0.45 (-1.09 to 0.19)	-0.39 (-1.00 to 0.21)	-0.15 (-0.78 to 0.48)	-0.06 (-0.76 to 0.64)	-0.05 (-0.66 to 0.56)	-0.03 (-0.58 to 0.53)	**DH**	-0.15 (-0.57 to 0.27)	NA
-1.13 (-1.74 to -0.53)	-0.93 (-1.35 to -0.51)	-0.97 (-1.69 to -0.25)	-0.92 (-1.38 to -0.46)	-0.60 (-1.09 to -0.11)	-0.54 (-0.99 to -0.10)	-0.30 (-0.77 to 0.17)	-0.21 (-0.78 to 0.35)	-0.20 (-0.65 to 0.25)	-0.18 (-0.54 to 0.19)	-0.15 (-0.57 to 0.27)	**SMD**	-0.38 (-1.30 to 0.54)
-1.84 (-2.71 to -0.97)	-1.63 (-2.55 to -0.72)	-1.67 (-2.76 to -0.59)	-1.63 (-2.56 to -0.69)	-1.30 (-2.25 to -0.36)	-1.25 (-2.14 to -0.35)	-1.00 (-1.94 to -0.07)	-0.92 (-1.91 to 0.07)	-0.91 (-1.68 to -0.13)	-0.88 (-1.71 to -0.05)	-0.85 (-1.77 to 0.06)	-0.70 (-1.52 to 0.11)	**aPDT TBO**

League table of pairwise comparisons in the NMA for CAL changes with split-mouth design studies.

**SA**	NA	NA	NA	NA	NA	NA	NA	NA	-1.20 (-1.35 to -1.05)	NA	NA	NA
-0.23 (-0.88 to 0.41)	**aPDT ICG**	NA	NA	NA	NA	NA	NA	-0.86 (-1.29 to -0.44)	NA	NA	NA	NA
-0.44 (-1.01 to 0.13)	-0.21 (-0.73 to 0.31)	**MINO micro spheres**	NA	NA	NA	NA	NA	-0.65 (-0.95 to -0.35)	NA	NA	NA	NA
-0.59 (-1.16 to -0.02)	-0.35 (-0.90 to 0.19)	-0.14 (-0.60 to 0.32)	**aPDT MB 0.005%**	NA	NA	NA	NA	-0.50 (-0.86 to -0.14)	-0.70 (-2.02 to 0.62)	NA	NA	NA
-0.59 (-1.45 to 0.27)	-0.36 (-1.18 to 0.47)	-0.15 (-0.92 to 0.62)	-0.00 (-0.79 to 0.78)	**MET**	NA	NA	NA	-0.50 (-1.21 to 0.20)	NA	NA	NA	NA
-0.57 (-1.77 to 0.63)	-0.34 (-1.52 to 0.84)	-0.13 (-1.27 to 1.01)	0.01 (-1.14 to 1.16)	0.02 (-1.29 to 1.32)	**MINO gel**	NA	NA	-0.52 (-1.62 to 0.58)	NA	NA	NA	NA
-0.96 (-1.58 to -0.33)	-0.73 (-1.31 to -0.15)	-0.51 (-1.01 to -0.02)	-0.37 (-0.90 to 0.15)	-0.37 (-1.18 to 0.44)	-0.38 (-1.55 to 0.78)	**DH**	NA	-0.13 (-0.53 to 0.26)	NA	NA	NA	NA
-0.99 (-1.56 to -0.42)	-0.76 (-1.28 to -0.24)	-0.55 (-0.98 to -0.12)	-0.41 (-0.87 to 0.05)	-0.40 (-1.17 to 0.37)	-0.42 (-1.56 to 0.72)	-0.04 (-0.53 to 0.46)	**CHX chip**	-0.10 (-0.40 to 0.20)	NA	NA	NA	NA
-1.09 (-1.58 to -0.61)	-0.86 (-1.29 to -0.44)	-0.65 (-0.95 to -0.35)	-0.51 (-0.85 to -0.16)	-0.50 (-1.21 to 0.20)	-0.52 (-1.62 to 0.58)	-0.14 (-0.53 to 0.26)	-0.10 (-0.40 to 0.20)	**SMD**	0.01 (-1.11 to 1.13)	-0.08 (-0.30 to 0.14)	-0.42 (-1.61 to 0.77)	-0.56 (-1.95 to 0.83)
-1.20 (-1.35 to -1.05)	-0.97 (-1.59 to -0.34)	-0.76 (-1.31 to -0.21)	-0.61 (-1.16 to -0.06)	-0.61 (-1.45 to 0.23)	-0.63 (-1.82 to 0.56)	-0.24 (-0.85 to 0.36)	-0.21 (-0.76 to 0.34)	-0.11 (-0.57 to 0.35)	**placebo**	0.04 (-0.45 to 0.53)	-0.43 (-1.77 to 0.91)	NA
-1.17 (-1.63 to -0.71)	-0.94 (-1.42 to -0.46)	-0.73 (-1.10 to -0.35)	-0.58 (-0.99 to -0.18)	-0.58 (-1.32 to 0.16)	-0.60 (-1.72 to 0.52)	-0.21 (-0.66 to 0.24)	-0.18 (-0.55 to 0.20)	-0.08 (-0.30 to 0.14)	0.03 (-0.40 to 0.46)	**aPDT MB 1%**	NA	NA
-1.55 (-2.73 to -0.38)	-1.32 (-2.54 to -0.11)	-1.11 (-2.29 to 0.07)	-0.97 (-2.15 to 0.22)	-0.96 (-2.30 to 0.38)	-0.98 (-2.56 to 0.60)	-0.60 (-1.80 to 0.61)	-0.56 (-1.74 to 0.62)	-0.46 (-1.60 to 0.68)	-0.35 (-1.52 to 0.81)	-0.38 (-1.53 to 0.77)	**aPDT TBO**	NA
-1.65 (-3.12 to -0.18)	-1.42 (-2.87 to 0.03)	-1.21 (-2.63 to 0.21)	-1.07 (-2.49 to 0.36)	-1.06 (-2.62 to 0.49)	-1.08 (-2.85 to 0.69)	-0.69 (-2.14 to 0.75)	-0.66 (-2.08 to 0.76)	-0.56 (-1.95 to 0.83)	-0.45 (-1.91 to 1.01)	-0.48 (-1.89 to 0.92)	-0.10 (-1.89 to 1.69)	**CHX xanthan**

League table of pairwise comparisons in the NMA for PPD changes with parallel design studies.

**DH**	NA	NA	NA	NA	-0.73 (-1.26 to -0.20)	NA	NA	NA	NA	NA
-0.21 (-0.94 to 0.51)	**aPDT MB 1%**	NA	NA	NA	-0.09 (-1.07 to 0.89)	NA	NA	-0.80 (-1.30 to -0.30)	NA	NA
-0.23 (-0.98 to 0.52)	-0.02 (-0.66 to 0.62)	**MINO gel**	NA	NA	NA	NA	NA	-0.67 (-1.12 to -0.22)	NA	NA
-0.59 (-1.31 to 0.13)	-0.38 (-1.07 to 0.32)	-0.36 (-1.08 to 0.36)	**CHX xanthan**	NA	-0.14 (-0.62 to 0.34)	NA	NA	NA	NA	NA
-0.64 (-1.24 to -0.04)	-0.43 (-0.94 to 0.08)	-0.41 (-0.94 to 0.11)	-0.05 (-0.61 to 0.51)	**MINO micro spheres**	-0.11 (-0.40 to 0.19)	NA	NA	-0.24 (-0.51 to 0.03)	NA	NA
-0.73 (-1.26 to -0.20)	-0.52 (-1.02 to -0.02)	-0.50 (-1.03 to 0.03)	-0.14 (-0.62 to 0.34)	-0.09 (-0.37 to 0.20)	**placebo**	NA	NA	-0.13 (-0.42 to 0.16)	NA	NA
-0.83 (-1.55 to -0.10)	-0.62 (-1.23 to 0.00)	-0.60 (-1.21 to 0.01)	-0.24 (-0.93 to 0.46)	-0.19 (-0.68 to 0.31)	-0.10 (-0.60 to 0.40)	**aPDT PC**	NA	-0.07 (-0.49 to 0.34)	NA	NA
-0.92 (-1.72 to -0.11)	-0.70 (-1.41 to 0.00)	-0.69 (-1.39 to 0.02)	-0.33 (-1.10 to 0.45)	-0.27 (-0.88 to 0.33)	-0.19 (-0.79 to 0.42)	-0.09 (-0.77 to 0.59)	**CHX chip**	0.02 (-0.52 to 0.55)	-0.56 (-1.69 to 0.57)	NA
-0.90 (-1.50 to -0.30)	-0.69 (-1.14 to -0.24)	-0.67 (-1.12 to -0.22)	-0.31 (-0.87 to 0.25)	-0.26 (-0.53 to 0.01)	-0.17 (-0.45 to 0.11)	-0.07 (-0.49 to 0.34)	0.02 (-0.52 to 0.55)	**SMD**	0.06 (-0.88 to 1.00)	-0.08 (-0.64 to 0.48)
-1.04 (-2.11 to 0.04)	-0.82 (-1.83 to 0.18)	-0.81 (-1.81 to 0.20)	-0.45 (-1.50 to 0.61)	-0.39 (-1.33 to 0.54)	-0.31 (-1.25 to 0.63)	-0.21 (-1.20 to 0.78)	-0.12 (-1.08 to 0.84)	-0.14 (-1.03 to 0.76)	**TC**	NA
-0.98 (-1.80 to -0.16)	-0.77 (-1.49 to -0.05)	-0.75 (-1.47 to -0.03)	-0.39 (-1.18 to 0.40)	-0.34 (-0.96 to 0.28)	-0.25 (-0.88 to 0.38)	-0.15 (-0.85 to 0.55)	-0.06 (-0.84 to 0.71)	-0.08 (-0.64 to 0.48)	0.06 (-1.00 to 1.11)	**PI 10%**

League table of pairwise comparisons in the NMA for CAL changes with parallel design studies.

**DH**	NA	NA	NA	NA	NA	NA	NA	NA	-0.87 (-1.37 to -0.37)	NA
-0.15 (-0.83 to 0.53)	**MINO gel**	NA	NA	NA	NA	NA	NA	-0.69 (-1.08 to -0.30)	NA	NA
-0.34 (-1.16 to 0.48)	-0.19 (-0.91 to 0.53)	**CHX chip**	NA	NA	NA	NA	-1.00 (-2.00 to 0.00)	-0.50 (-1.10 to 0.10)	NA	NA
-0.45 (-1.17 to 0.27)	-0.30 (-0.92 to 0.32)	-0.11 (-0.88 to 0.66)	**aPDT MB 1%**	NA	NA	NA	NA	-0.55 (-1.09 to -0.01)	0.22 (-0.85 to 1.29)	NA
-0.64 (-1.29 to 0.01)	-0.49 (-1.00 to 0.02)	-0.30 (-0.99 to 0.39)	-0.19 (-0.77 to 0.39)	**aPDT PC**	NA	NA	NA	-0.20 (-0.53 to 0.13)	NA	NA
-0.68 (-1.50 to 0.14)	-0.53 (-1.32 to 0.26)	-0.34 (-1.25 to 0.58)	-0.23 (-1.05 to 0.60)	-0.04 (-0.80 to 0.72)	**CHX xanthan**	NA	NA	NA	-0.19 (-0.83 to 0.45)	NA
-0.71 (-1.27 to -0.15)	-0.56 (-1.01 to -0.10)	-0.37 (-1.02 to 0.28)	-0.26 (-0.78 to 0.27)	-0.07 (-0.47 to 0.34)	-0.03 (-0.72 to 0.66)	**MINO micro spheres**	NA	-0.12 (-0.36 to 0.12)	-0.15 (-0.41 to 0.10)	NA
-0.91 (-1.95 to 0.13)	-0.76 (-1.72 to 0.19)	-0.57 (-1.48 to 0.33)	-0.46 (-1.46 to 0.54)	-0.27 (-1.21 to 0.66)	-0.23 (-1.35 to 0.88)	-0.21 (-1.11 to 0.70)	**TC**	-0.25 (-1.18 to 0.68)	NA	NA
-0.84 (-1.40 to -0.28)	-0.69 (-1.08 to -0.30)	-0.50 (-1.10 to 0.10)	-0.39 (-0.87 to 0.09)	-0.20 (-0.53 to 0.13)	-0.16 (-0.85 to 0.53)	-0.13 (-0.37 to 0.11)	0.07 (-0.80 to 0.95)	**SMD**	-0.09 (-0.35 to 0.16)	-0.45 (-1.27 to 0.37)
-0.87 (-1.37 to -0.37)	-0.72 (-1.18 to -0.26)	-0.53 (-1.18 to 0.12)	-0.42 (-0.94 to 0.10)	-0.23 (-0.64 to 0.18)	-0.19 (-0.83 to 0.45)	-0.16 (-0.41 to 0.09)	0.04 (-0.87 to 0.95)	-0.03 (-0.27 to 0.22)	**placebo**	NA
-1.29 (-2.28 to -0.30)	-1.14 (-2.05 to -0.23)	-0.95 (-1.97 to 0.07)	-0.84 (-1.79 to 0.11)	-0.65 (-1.53 to 0.23)	-0.61 (-1.68 to 0.46)	-0.58 (-1.44 to 0.27)	-0.38 (-1.58 to 0.82)	-0.45 (-1.27 to 0.37)	-0.42 (-1.28 to 0.43)	**PI 10%**

Figure S2. Inconsistency plot for the network meta-analysis.

1) Inconsistency plot for the network meta-analysis of PPD and CAL changes in split-mouth design studies

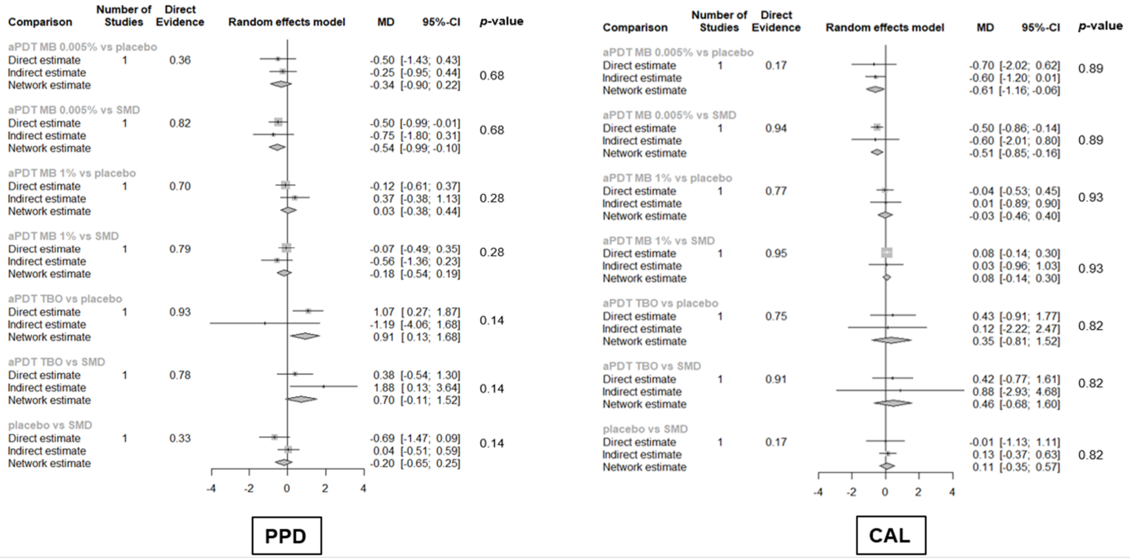

2) Inconsistency plot for the network meta-analysis of PPD and CAL changes in parallel design studies

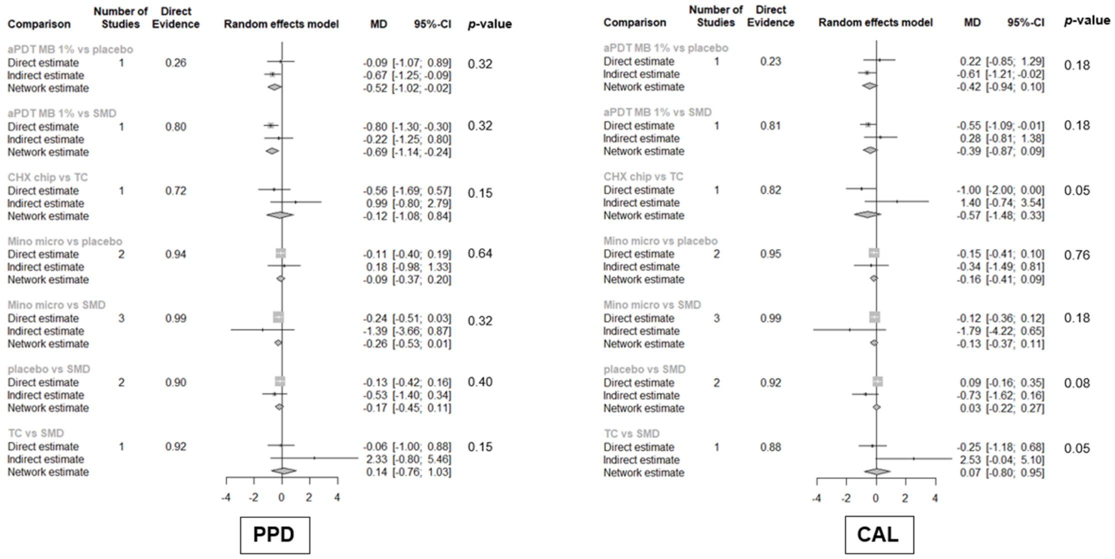

Figure S3. Comparison-adjusted funnel plot for publication and small-study bias.

1) Comparison-adjusted funnel plot for the network of single LDA application with split-mouth design for PPD and CAL changes

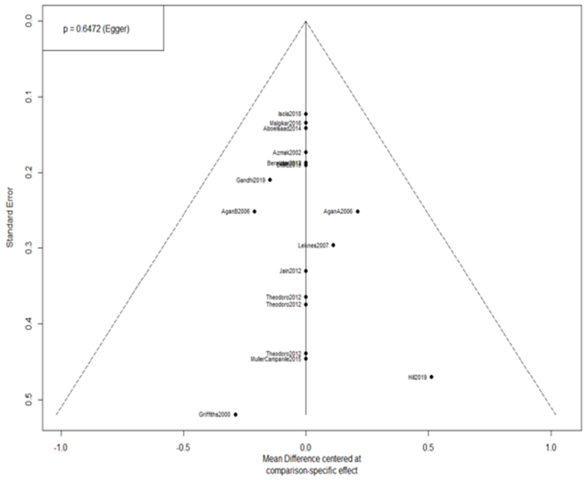

PPD

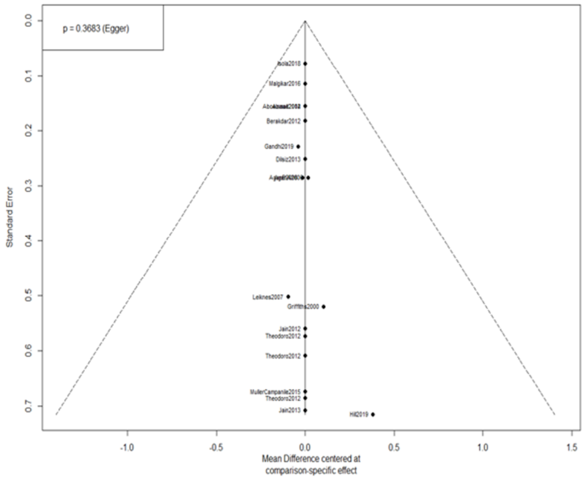

CAL

2) Comparison-adjusted funnel plot for the network of single LDA application with parallel design for PPD and CAL changes

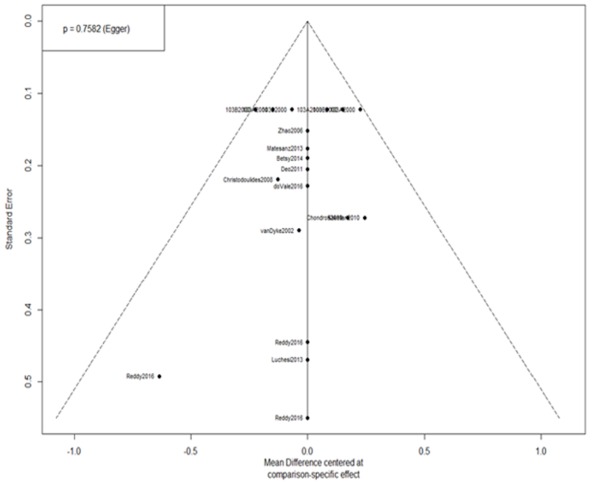

PPD

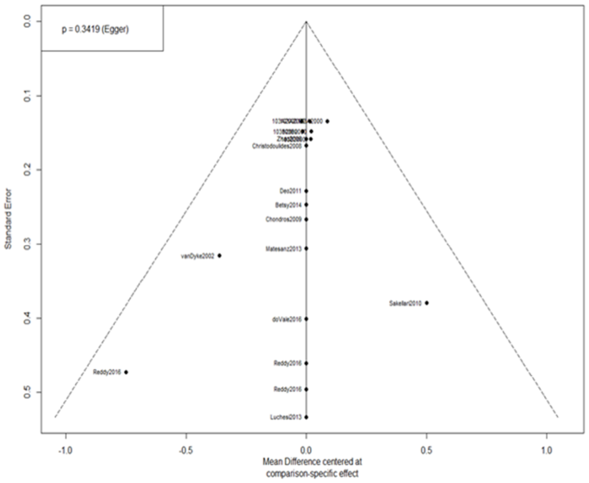

CAL

Table S8. Grading of Recommendations Assessment, Development and Evaluation (GRADE) certainty of evidence for primary outcomes.

Mean differences (95% CIs) and certainty of evidence for PPD changes in split-mouth design

Comparisons	GRADE					
	Direct evidence		Indirect evidence		Network meta-analysis	
	Mean difference (95% CI)	Certainty of evidence	Mean difference (95% CI)	Certainty of evidence	Mean difference (95% CI)	Certainty of evidence
**aPDT ICG vs.**						
aPDT MB 0.005%	-	-	-0.38 (-1.02 to 0.26)	⊕⊕◯◯ [e, c] Low	-0.38 (-1.02 to 0.26)	⊕◯◯◯ [c, f] Very Low
aPDT MB 1%	-	-	-0.75 (-1.34 to -0.16)	⊕⊕⊕◯ [e] Moderate	-0.75 (-1.34 to -0.16)	⊕⊕⊕◯ [e] Moderate
aPDT TBO	-	-	-1.63 (-2.56 to -0.69)	⊕⊕⊕◯ [e] Moderate	-1.63 (-2.56 to -0.69)	⊕⊕⊕◯ [e] Moderate
CHX chip	-	-	-0.62 (-1.28 to 0.03)	⊕⊕⊕◯ [e] Moderate	-0.62 (-1.28 to 0.03)	⊕⊕◯◯ [e, c] Low
CHX xanthan	-	-	-0.32 (-0.99 to 0.35)	⊕⊕◯◯ [e, c] Low	-0.32 (-0.99 to 0.35)	⊕◯◯◯ [c, f] Very Low
DH	-	-	-0.77 (-1.39 to -0.15)	⊕⊕⊕◯ [e] Moderate	-0.77 (-1.39 to -0.15)	⊕⊕⊕◯ [e] Moderate
MET	-	-	-0.71 (-1.44 to 0.02)	⊕⊕◯◯ [e, c] Low	-0.71 (-1.44 to 0.02)	⊕◯◯◯ [c, f] Very Low
MINO gel	-	-	0.05 (-0.81 to 0.90)	⊕⊕◯◯ [e, c] Low	0.05 (-0.81 to 0.90)	⊕◯◯◯ [c, f] Very Low
MINO microspheres	-	-	0.01 (-0.62 to 0.63)	⊕⊕◯◯ [e, c] Low	0.01 (-0.62 to 0.63)	⊕◯◯◯ [c, f] Very Low
placebo	-	-	-0.72 (-1.36 to -0.08)	⊕⊕◯◯ [e, c] Low	-0.72 (-1.36 to -0.08)	⊕⊕⊕◯ [e] Moderate
SA	-	-	0.21 (-0.55 to 0.97)	⊕⊕◯◯ [e, c] Low	0.21 (-0.55 to 0.97)	⊕⊕◯◯ [e, c] Low
SMD	-0.92 (-1.38 to -0.46)	⊕⊕⊕◯ [a] Moderate	-	-	-0.92 (-1.38 to -0.46)	⊕⊕⊕◯ [e] Moderate
**aPDT MB 0.005% vs.**						
aPDT MB 1%	-	-	-0.37 (-0.91 to 0.18)	⊕⊕◯◯ [e, c] Low	-0.37 (-0.91 to 0.18)	⊕◯◯◯ [c, f] Very Low
aPDT TBO	-	-	-1.25 (-2.14 to -0.35)	⊕⊕⊕◯ [e] Moderate	-1.25 (-2.14 to -0.35)	⊕⊕⊕◯ [e] Moderate

CHX chip	-	-	-0.24 (-0.89 to 0.40)	⊕⊕◯◯ [e, c] Low	-0.24 (-0.89 to 0.40)	⊕◯◯◯ [c, f] Very Low
CHX xanthan	-	-	0.06 (-0.60 to 0.72)	⊕⊕◯◯ [e, c] Low	0.06 (-0.60 to 0.72)	⊕◯◯◯ [c, f] Very Low
DH	-	-	-0.39 (-1.00 to 0.21)	⊕⊕◯◯ [e, c] Low	-0.39 (-1.00 to 0.21)	⊕◯◯◯ [c, f] Very Low
MET	-	-	-0.33 (-1.05 to 0.39)	⊕⊕◯◯ [e, c] Low	-0.33 (-1.05 to 0.39)	⊕◯◯◯ [c, f] Very Low
MINO gel	-	-	0.43 (-0.42 to 1.27)	⊕⊕◯◯ [e, c] Low	0.43 (-0.42 to 1.27)	⊕◯◯◯ [c, f] Very Low
MINO microspheres	-	-	0.39 (-0.23 to 1.00)	⊕⊕◯◯ [e, c] Low	0.39 (-0.23 to 1.00)	⊕◯◯◯ [c, f] Very Low
placebo	-0.50 (-1.43 to 0.43)	⊕⊕◯◯ [a, c] Low	-0.25 (-0.95 to 0.44)	⊕⊕◯◯ [e, c] Low	-0.34 (-0.90 to 0.22)	⊕◯◯◯ [c, f] Very Low
SA	-	-	0.59 (-0.1 to 1.28)	⊕⊕◯◯ [e, c] Low	0.59 (-0.1 to 1.28)	⊕◯◯◯ [c, f] Very Low
SMD	-0.50 (-0.99 to -0.01)	⊕⊕⊕◯ [a] Moderate	-0.75 (-1.80 to 0.31)	⊕⊕◯◯ [e, c] Low	-0.54 (-0.99 to -0.10)	⊕⊕⊕◯ [e] Moderate
**aPDT MB 1% vs.**						
aPDT TBO	-	-	-0.88 (-1.71 to -0.05)	⊕⊕⊕◯ [e] Moderate	-0.88 (-1.71 to -0.05)	⊕⊕⊕◯ [e] Moderate
CHX chip	-	-	0.12 (- 0.47 to 0.72)	⊕⊕◯◯ [e, c] Low	0.12 (- 0.47 to 0.72)	⊕◯◯◯ [c, f] Very Low
CHX xanthan	-	-	0.42 (-0.19 to 1.04)	⊕⊕◯◯ [e, c] Low	0.42 (-0.19 to 1.04)	⊕◯◯◯ [c, f] Very Low
DH	-	-	-0.03 (-0.58 to 0.53)	⊕⊕◯◯ [e, c] Low	-0.03 (-0.58 to 0.53)	⊕◯◯◯ [c, f] Very Low
MET	-	-	0.04 (- 0.64 to 0.71)	⊕⊕◯◯ [e, c] Low	0.04 (- 0.64 to 0.71)	⊕◯◯◯ [c, f] Very Low
MINO gel	-	-	0.79 (-0.02 to 1.61)	⊕⊕◯◯ [e, c] Low	0.79 (-0.02 to 1.61)	⊕◯◯◯ [c, f] Very Low
MINO microspheres	-	-	0.75 (0.19 to 1.32)	⊕⊕⊕◯ [e] Moderate	0.75 (0.19 to 1.32)	⊕⊕⊕◯ [e] Moderate
placebo	-0.12 (-0.37 to 0.61)	⊕⊕◯◯ [a, c] Low	0.03 (-0.38 to 0.44)	⊕⊕◯◯ [e, c] Low	0.03 (-0.38 to 0.44)	⊕◯◯◯ [c, f] Very Low
SA	-	-	0.96 (0.38 to 1.53)	⊕⊕⊕◯ [e] Moderate	0.96 (0.38 to 1.53)	⊕⊕⊕◯ [e] Moderate

SMD	-0.07 (-0.49 to 0.35)	⊕⊕◯◯ [a, c]<br>Low	-0.56 (-1.36 to 0.23)	⊕⊕◯◯ [e, c]<br>Low	-0.18 (-0.54 to 0.19)	⊕◯◯◯ [c, f]<br>Very Low
**aPDT TBO vs.**						
CHX chip	-	-	1.00 (0.07 to 1.94)	⊕⊕⊕◯ [e]<br>Moderate	1.00 (0.07 to 1.94)	⊕⊕⊕◯ [e]<br>Moderate
CHX xanthan	-	-	1.30 (0.36 to 2.25)	⊕⊕⊕◯ [e]<br>Moderate	1.30 (0.36 to 2.25)	⊕⊕⊕◯ [e]<br>Moderate
DH	-	-	0.85 (-0.06 to 1.77)	⊕⊕◯◯ [e, c]<br>Low	0.85 (-0.06 to 1.77)	⊕◯◯◯ [c, f]<br>Very Low
MET	-	-	0.92 (-0.07 to 1.91	⊕⊕◯◯ [e, c]<br>Low	0.92 (-0.07 to 1.91	⊕◯◯◯ [c, f]<br>Very Low
MINO gel	-	-	1.67 (0.59 to 2.76)	⊕⊕⊕◯ [e]<br>Moderate	1.67 (0.59 to 2.76)	⊕⊕⊕◯ [e]<br>Moderate
MINO microspheres	-	-	1.63 (0.72 to 2.55)	⊕⊕⊕◯ [e]<br>Moderate	1.63 (0.72 to 2.55)	⊕⊕⊕◯ [e]<br>Moderate
placebo	1.07 (0.27 to 1.87)	⊕⊕⊕◯ [a]<br>Moderate	-1.19 (-4.06 to 1.68)	⊕◯◯◯ [d, f]<br>Very Low	0.91 (0.13 to 1.68)	⊕⊕⊕◯ [e]<br>Moderate
SA	-	-	1.84 (-0.97 to 2.71)	⊕⊕◯◯ [e, c]<br>Low	1.84 (-0.97 to 2.71)	⊕◯◯◯ [c, f]<br>Very Low
SMD	0.38 (-0.54 to 1.30)	⊕⊕◯◯ [a, c]<br>Low	1.88 (0.13 to 3.64)	⊕⊕⊕◯ [e]<br>Moderate	0.70 (- 0.11 to 1.52)	⊕⊕◯◯ [e, c]<br>Low
**CHX chip vs.**						
CHX xanthan	-	-	0.30 (- 0.38 to 0.98)	⊕⊕◯◯ [e, c]<br>Low	0.30 (- 0.38 to 0.98)	⊕◯◯◯ [c, f]<br>Very Low
DH	-	-	-0.15 (-0.78 to 0.48)	⊕⊕◯◯ [e, c]<br>Low	-0.15 (-0.78 to 0.48)	⊕◯◯◯ [c, f]<br>Very Low
MET	-	-	-0.09 (-0.82 to 0.64)	⊕⊕◯◯ [e, c]<br>Low	-0.09 (-0.82 to 0.64)	⊕◯◯◯ [c, f]<br>Very Low
MINO gel	-	-	0.67 (-0.19 to 1.53)	⊕⊕◯◯ [e, c]<br>Low	0.67 (-0.19 to 1.53)	⊕◯◯◯ [c, f]<br>Very Low
MINO microspheres	-	-	0.63 (0.00 to 1.26)	⊕⊕⊕◯ [e]<br>Moderate	0.63 (0.00 to 1.26)	⊕⊕⊕◯ [e]<br>Moderate
placebo	-	-	-0.10 (-0.75 to 0.55)	⊕⊕◯◯ [e, c]<br>Low	-0.10 (-0.75 to 0.55)	⊕◯◯◯ [c, f]<br>Very Low
SA	-	-	0.83 (0.07 to 1.60)	⊕⊕⊕◯ [e]<br>Moderate	0.83 (0.07 to 1.60)	⊕⊕⊕◯ [e]<br>Moderate

SMD	-0.30 (-0.77 to 0.17)	⊕⊕◯◯ [a, c] Low	-	-	-0.30 (-0.77 to 0.17)	⊕◯◯◯ [c, f] Very Low
**CHX xanthan vs.**						
DH	-	-	-0.45 (-1.09 to 0.19)	⊕⊕◯◯ [e, c] Low	-0.45 (-1.09 to 0.19)	⊕◯◯◯ [c, f] Very Low
MET	-	-	-0.39 (-1.13 to 0.36)	⊕⊕◯◯ [e, c] Low	-0.39 (-1.13 to 0.36)	⊕◯◯◯ [c, f] Very Low
MINO gel	-	-	0.37 (-0.50 to 1.24)	⊕⊕◯◯ [e, c] Low	0.37 (-0.50 to 1.24)	⊕◯◯◯ [c, f] Very Low
MINO microspheres	-	-	0.33 (-0.32 to 0.98)	⊕⊕◯◯ [e, c] Low	0.33 (-0.32 to 0.98)	⊕◯◯◯ [c, f] Very Low
placebo	-	-	-0.40 (-1.06 to 0.27)	⊕⊕◯◯ [e, c] Low	-0.40 (-1.06 to 0.27)	⊕◯◯◯ [c, f] Very Low
SA	-	-	0.53 (-0.24 to 1.31)	⊕⊕◯◯ [e, c] Low	0.53 (-0.24 to 1.31)	⊕◯◯◯ [c, f] Very Low
SMD	-0.60 (-1.09 to -0.11)	⊕⊕⊕◯ [a] Moderate	-	-	-0.60 (-1.09 to -0.11)	⊕⊕⊕◯ [e] Moderate
**DH vs.**						
MET	-	-	0.06 (-0.64 to 0.76)	⊕⊕◯◯ [e, c] Low	0.06 (-0.64 to 0.76)	⊕◯◯◯ [c, f] Very Low
MINO gel	-	-	0.82 (-0.01 to 1.65)	⊕⊕◯◯ [e, c] Low	0.82 (-0.01 to 1.65)	⊕◯◯◯ [c, f] Very Low
MINO microspheres	-	-	0.78 (0.19 to 1.37)	⊕⊕⊕◯ [e] Moderate	0.78 (0.19 to 1.37)	⊕⊕⊕◯ [e] Moderate
placebo	-	-	0.05 (0.56 to 0.66)	⊕⊕⊕◯ [e] Moderate	0.05 (0.56 to 0.66)	⊕⊕⊕◯ [e] Moderate
SA	-	-	0.98 (0.25 to 1.71)	⊕⊕⊕◯ [e] Moderate	0.98 (0.25 to 1.71)	⊕⊕⊕◯ [e] Moderate
SMD	-0.15 (-0.57 to 0.27)	⊕⊕◯◯ [a, c] Low	-	-	-0.15 (-0.57 to 0.27)	⊕◯◯◯ [c, f] Very Low
**MET vs.**						
MINO gel	-	-	0.76 (-0.16 to 1.68)	⊕⊕◯◯ [e, c] Low	0.76 (-0.16 to 1.68)	⊕◯◯◯ [c, f] Very Low
MINO microspheres	-	-	0.72 (0.01 to 1.42)	⊕⊕⊕◯ [e] Moderate	0.72 (0.01 to 1.42)	⊕⊕⊕◯ [e] Moderate
placebo	-	-	-0.01 (-0.73 to 0.71)	⊕⊕◯◯ [e, c] Low	-0.01 (-0.73 to 0.71)	⊕◯◯◯ [c, f] Very Low

SA	-	-	0.92 (0.10 to 1.75)	⊕⊕⊕◯ [e] Moderate	0.92 (0.10 to 1.75)	⊕⊕⊕◯ [e] Moderate
SMD	-0.21 (-0.78 to 0.35)	⊕⊕◯◯ [a, c] Low	-	-	-0.21 (-0.78 to 0.35)	⊕◯◯◯ [c, f] Very Low
**MINO gel vs.**						
MINO microspheres	-	-	-0.04 (-0.88 to 0.80)	⊕⊕◯◯ [e, c] Low	-0.04 (-0.88 to 0.80)	⊕◯◯◯ [c, f] Very Low
placebo	-	-	-0.77 (-1.62 to 0.08)	⊕⊕◯◯ [e, c] Low	-0.77 (-1.62 to 0.08)	⊕◯◯◯ [c, f] Very Low
SA	-	-	0.16 (-0.78 to 1.10)	⊕⊕◯◯ [e, c] Low	0.16 (-0.78 to 1.10)	⊕◯◯◯ [c, f] Very Low
SMD	-0.97 (-1.69 to -0.25)	⊕⊕⊕◯ [a] Moderate	-	-	-0.97 (-1.69 to -0.25)	⊕⊕⊕◯ [e] Moderate
**MINO microspheres vs.**						
placebo	-	-	-0.73 (-1.35 to -0.11)	⊕⊕⊕◯ [e] Moderate	-0.73 (-1.35 to -0.11)	⊕⊕⊕◯ [e] Moderate
SA	-	-	0.20 (- 0.53 to 0.94)	⊕⊕◯◯ [e, c] Low	0.20 (- 0.53 to 0.94)	⊕◯◯◯ [c, f] Very Low
SMD	-0.93 (-1.35 to -0.51)	⊕⊕⊕◯ [a] Moderate	-	-	-0.93 (-1.35 to -0.51)	⊕⊕⊕◯ [e] Moderate
**Placebo vs.**						
SA	0.93 (0.53 to 1.33)	⊕⊕⊕◯ [a] Moderate	-	-	0.93 (0.53 to 1.33)	⊕⊕⊕◯ [e] Moderate
SMD	-0.69 (-1.47 to 0.09)	⊕⊕◯◯ [a, c] Low	0.04 (-0.51 to 0.59)	⊕⊕◯◯ [e, c] Low	-0.20 (-0.65 to 0.25)	⊕◯◯◯ [c, f] Very Low
**SA vs. SMD**	-	-	-1.13 (-1.74 to -0.53)	⊕⊕⊕◯ [e] Moderate	-1.13 (-1.74 to -0.53)	⊕⊕⊕◯ [e] Moderate

[a] Limitations (risk of bias). [b] Inconsistency. [c] Imprecision. [d] Severe imprecision. [e] Contributing evidence of moderate quality. [f] Contributing evidence of low or very low quality. [g] Incoherence

Mean differences (95% CIs) and certainty of evidence for PPD changes in parallel design

**Comparisons**	**GRADE**					
	**Direct evidence**		**Indirect evidence**		**Network meta-analysis**	
	**Mean difference (95% CI)**	**Certainty of evidence**	**Mean difference (95% CI)**	**Certainty of evidence**	**Mean difference (95% CI)**	**Certainty of evidence**

**aPDT MB 1% vs.**						
aPDT PC	-	-	-0.62 (-1.23 to 0.00)	⊕⊕◯◯ [e, c] Low	-0.62 (-1.23 to 0.00)	⊕◯◯◯ [c, f] Very Low
CHX chip	-	-	-0.70 (-1.41 to 0.00)	⊕⊕◯◯ [e, c] Low	-0.70 (-1.41 to 0.00)	⊕◯◯◯ [c, f] Very Low
CHX xanthan	-	-	-0.38 (-1.07 to 0.32)	⊕⊕◯◯ [e, c] Low	-0.38 (-1.07 to 0.32)	⊕◯◯◯ [c, f] Very Low
DH	-	-	0.21 (-0.51 to 0.94)	⊕⊕◯◯ [e, c] Low	0.21 (-0.51 to 0.94)	⊕◯◯◯ [c, f] Very Low
MINO gel	-	-	-0.02 (-0.66 to 0.62)	⊕⊕◯◯ [e, c] Low	-0.02 (-0.66 to 0.62)	⊕◯◯◯ [c, f] Very Low
MINO microspheres	-	-	-0.43 (-0.94 to 0.08)	⊕⊕◯◯ [e, c] Low	-0.43 (-0.94 to 0.08)	⊕◯◯◯ [c, f] Very Low
PI 10%	-	-	-0.77 (-1.49 to -0.05)	⊕⊕⊕◯ [e] Moderate	-0.77 (-1.49 to -0.05)	⊕⊕⊕◯ [e] Moderate
placebo	-0.09 (-1.07 to 0.89)	⊕⊕◯◯ [a, c] Low	-0.67 (-1.25 to -0.09)	⊕⊕⊕◯ [e] Moderate	-0.52 (-1.02 to -0.02)	⊕⊕⊕◯ [e] Moderate
SMD	-0.80 (-1.30 to -0.30)	⊕⊕◯◯ [a, c] Low	-0.22 (-1.25 to 0.80)	⊕⊕◯◯ [e, c] Low	-0.69 (-1.14 to -0.24)	⊕⊕⊕◯ [e] Moderate
TC	-	-	-0.82 (-1.83 to 0.18)	⊕⊕◯◯ [e, c] Low	-0.82 (-1.83 to 0.18)	⊕◯◯◯ [c, f] Very Low
**aPDT PC vs.**						
CHX chip	-	-	-0.09 (-0.77 to 0.59)	⊕⊕◯◯ [e, c] Low	-0.09 (-0.77 to 0.59)	⊕◯◯◯ [c, f] Very Low
CHX xanthan	-	-	0.24 (-0.46 to 0.93)	⊕⊕◯◯ [e, c] Low	0.24 (-0.46 to 0.93)	⊕◯◯◯ [c, f] Very Low
DH	-	-	0.83 (0.10 to 1.55)	⊕⊕⊕◯ [e] Moderate	0.83 (0.10 to 1.55)	⊕⊕⊕◯ [e] Moderate
MINO gel	-	-	0.60 (-0.01 to 1.21)	⊕⊕◯◯ [e, c] Low	0.60 (-0.01 to 1.21)	⊕◯◯◯ [c, f] Very Low
MINO microspheres	-	-	0.19 (- 0.31 to 0.68)	⊕⊕◯◯ [e, c] Low	0.19 (- 0.31 to 0.68)	⊕◯◯◯ [c, f] Very Low
PI 10%	-	-	-0.15 (-0.85 to 0.55)	⊕⊕◯◯ [e, c] Low	-0.15 (-0.85 to 0.55)	⊕◯◯◯ [c, f] Very Low
placebo	-	-	0.10 (-0.40 to 0.60)	⊕⊕◯◯ [e, c] Low	0.10 (-0.40 to 0.60)	⊕◯◯◯ [c, f] Very Low

SMD	-0.07 (-0.49 to 0.34)	⊕⊕◯◯ [a, c]<br>Low	-	-	-0.07 (-0.49 to 0.34)	⊕◯◯◯ [c, f]<br>Very Low
TC	-	-	-0.21 (-1.20 to 0.78)	⊕⊕◯◯ [e, c]<br>Low	-0.21 (-1.20 to 0.78)	⊕◯◯◯ [c, f]<br>Very Low
**CHX chip vs.**						
CHX xanthan	-	-	0.33 (- 0.45 to 1.10)	⊕⊕◯◯ [e, c]<br>Low	0.33 (- 0.45 to 1.10)	⊕◯◯◯ [c, f]<br>Very Low
DH	-	-	0.92 (0.11 to 1.72)	⊕⊕⊕◯ [e]<br>Moderate	0.92 (0.11 to 1.72)	⊕⊕⊕◯ [e]<br>Moderate
MINO gel	-	-	0.69 (-0.02 to 1.39)	⊕⊕◯◯ [e, c]<br>Low	0.69 (-0.02 to 1.39)	⊕◯◯◯ [c, f]<br>Very Low
MINO microspheres	-	-	0.27 (-0.33 to 0.88)	⊕⊕◯◯ [e, c]<br>Low	0.27 (-0.33 to 0.88)	⊕◯◯◯ [c, f]<br>Very Low
PI 10%	-	-	-0.06 (-0.84 to 0.71)	⊕⊕◯◯ [e, c]<br>Low	-0.06 (-0.84 to 0.71)	⊕◯◯◯ [c, f]<br>Very Low
placebo	-	-	0.19 (-0.42 to 0.79)	⊕⊕◯◯ [e, c]<br>Low	0.19 (-0.42 to 0.79)	⊕◯◯◯ [c, f]<br>Very Low
SMD	0.02 (-0.52 to 0.55)	⊕⊕◯◯ [a, c]<br>Low	-	-	0.02 (-0.52 to 0.55)	⊕◯◯◯ [c, f]<br>Very Low
TC	-0.56 (-1.69 to 0.57)	⊕⊕◯◯ [a, c]<br>Low	0.99 (-0.80 to 2.79)	⊕◯◯◯ [d, f]<br>Very Low	-0.12 (-1.08 to 0.84)	⊕◯◯◯ [c, f]<br>Very Low
**CHX xanthan vs.**						
DH	-	-	0.59 (-0.13 to 1.31)	⊕⊕◯◯ [e, c]<br>Low	0.59 (-0.13 to 1.31)	⊕◯◯◯ [c, f]<br>Very Low
MINO gel	-	-	0.36 (-0.36 to 1.08)	⊕⊕◯◯ [e, c]<br>Low	0.36 (-0.36 to 1.08)	⊕◯◯◯ [c, f]<br>Very Low
MINO microspheres	-	-	-0.05 (-0.61 to 0.51)	⊕⊕◯◯ [e, c]<br>Low	-0.05 (-0.61 to 0.51)	⊕◯◯◯ [c, f]<br>Very Low
PI 10%	-	-	-0.39 (-1.18 to 0.40)	⊕⊕◯◯ [e, c]<br>Low	-0.39 (-1.18 to 0.40)	⊕◯◯◯ [c, f]<br>Very Low
placebo	-0.14 (-0.62 to 0.34)	⊕⊕◯◯ [a, c]<br>Low	-	-	-0.14 (-0.62 to 0.34)	⊕◯◯◯ [c, f]<br>Very Low
SMD	-	-	-0.31 (-0.87 to 0.25)	⊕⊕◯◯ [e, c]<br>Low	-0.31 (-0.87 to 0.25)	⊕◯◯◯ [c, f]<br>Very Low
TC	-	-	-0.45 (-1.50 to 0.61)	⊕⊕◯◯ [e, c]<br>Low	-0.45 (-1.50 to 0.61)	⊕◯◯◯ [c, f]<br>Very Low
**DH vs.**						

MINO gel	-	-	-0.23 (-0.98 to 0.52)	⊕⊕◯◯ [e, c] Low	-0.23 (-0.98 to 0.52)	⊕◯◯◯ [c, f] Very Low
MINO microspheres	-	-	-0.64 (-1.24 to -0.04)	⊕⊕⊕◯ [e] Moderate	-0.64 (-1.24 to -0.04)	⊕⊕⊕◯ [e] Moderate
PI 10%	-	-	-0.98 (-1.80 to -0.16)	⊕⊕⊕◯ [e] Moderate	-0.98 (-1.80 to -0.16)	⊕⊕⊕◯ [e] Moderate
placebo	-0.73 (-1.26 to -0.20)	⊕⊕⊕◯ [a] Moderate	-	-	-0.73 (-1.26 to -0.20)	⊕⊕⊕◯ [e] Moderate
SMD	-	-	-0.90 (-1.50 to -0.30)	⊕⊕⊕◯ [e] Moderate	-0.90 (-1.50 to -0.30)	⊕⊕⊕◯ [e] Moderate
TC	-	-	-1.04 (-2.11 to 0.04)	⊕◯◯◯ [d, f] Very Low	-1.04 (-2.11 to 0.04)	⊕◯◯◯ [c, f] Very Low
**MINO gel vs.**						
MINO microspheres	-	-	-0.41 (-0.94 to 0.11)	⊕⊕◯◯ [e, c] Low	-0.41 (-0.94 to 0.11)	⊕◯◯◯ [c, f] Very Low
PI 10%	-	-	-0.75 (-1.47 to -0.03)	⊕⊕⊕◯ [e] Moderate	-0.75 (-1.47 to -0.03)	⊕⊕⊕◯ [e] Moderate
placebo	-	-	-0.50 (-1.03 to 0.03)	⊕⊕◯◯ [e, c] Low	-0.50 (-1.03 to 0.03)	⊕◯◯◯ [c, f] Very Low
SMD	-0.67 (-1.12 to -0.22)	⊕⊕⊕◯ [a] Moderate	-	-	-0.67 (-1.12 to -0.22)	⊕⊕⊕◯ [e] Moderate
TC	-	-	-0.81 (-1.81 to 0.20)	⊕⊕◯◯ [e, c] Low	-0.81 (-1.81 to 0.20)	⊕◯◯◯ [c, f] Very Low
**MINO microspheres vs.**						
PI 10%	-	-	-0.34 (-0.96 to 0.28)	⊕⊕◯◯ [e, c] Low	-0.34 (-0.96 to 0.28)	⊕◯◯◯ [c, f] Very Low
placebo	-0.11 (-0.40 to 0.19)	⊕⊕◯◯ [a, c] Low	0.18 (-0.98 to 1.33)	⊕⊕◯◯ [e, c] Low	-0.09 (-0.37 to 0.20)	⊕⊕◯◯ [e, c] Low
SMD	-0.24 (-0.51 to 0.03)	⊕⊕◯◯ [a, c] Low	-1.39 (-3.66 to 0.87)	⊕◯◯◯ [d, f] Very Low	-0.26 (-0.53 to 0.01)	⊕◯◯◯ [c, f] Very Low
TC	-	-	-0.39 (-1.33 to 0.54)	⊕⊕◯◯ [e, c] Low	-0.39 (-1.33 to 0.54)	⊕◯◯◯ [c, f] Very Low
**PI 10% vs.**						
placebo	-	-	0.25 (- 0.38 to 0.88)	⊕⊕◯◯ [e, c] Low	0.25 (- 0.38 to 0.88)	⊕◯◯◯ [c, f] Very Low
SMD	-0.08 (-0.64 to 0.48)	⊕⊕◯◯ [a, c] Low	-	-	0.08 (-0.48 to 0.64)	⊕◯◯◯ [c, f] Very Low

TC	-	-	-0.06 (-1.11 to 1.00)	⊕⊕◯◯ [e, c] Low	-0.06 (-1.11 to 1.00)	⊕◯◯◯ [c, f] Very Low
**Placebo vs.**						
SMD	-0.13 (-0.42 to 0.16)	⊕⊕◯◯ [a, c] Low	-0.53 (-1.40 to 0.34)	⊕⊕◯◯ [e, c] Low	-0.17 (-0.45 to 0.11)	⊕◯◯◯ [c, f] Very Low
TC	-	-	-0.31 (-1.25 to 0.63)	⊕⊕◯◯ [e, c] Low	-0.31 (-1.25 to 0.63)	⊕◯◯◯ [c, f] Very Low
**TC vs. SMD**	0.06 (-0.88 to 1.00)	⊕⊕◯◯ [a, c] Low	2.33 (-0.80 to 5.46)	⊕◯◯◯ [d, f] Very Low	0.14 (-0.76 to 1.03)	⊕◯◯◯ [c, f] Very Low

[a] Limitations (risk of bias). [b] Inconsistency. [c] Imprecision. [d] Severe imprecision. [e] Contributing evidence of moderate quality. [f] Contributing evidence of low or very low quality. [g] Incoherence

Mean differences (95% CIs) and certainty of evidence for CAL changes in split-mouth design

Comparisons	GRADE					
	Direct evidence		Indirect evidence		Network meta-analysis	
	Mean difference (95% CI)	Certainty of evidence	Mean difference (95% CI)	Certainty of evidence	Mean difference (95% CI)	Certainty of evidence
**aPDT ICG vs.**						
aPDT MB 0.005%	-	-	-0.35 (-0.90 to 0.19)	⊕⊕◯◯ [e, c] Low	-0.35 (-0.90 to 0.19)	⊕◯◯◯ [c, f] Very Low
aPDT MB 1%	-	-	-0.94 (-1.42 to -0.46)	⊕⊕⊕◯ [e] Moderate	-0.94 (-1.42 to -0.46)	⊕⊕⊕◯ [e] Moderate
aPDT TBO	-	-	-1.32 (-2.54 to -0.11)	⊕⊕⊕◯ [e] Moderate	-1.32 (-2.54 to -0.11)	⊕⊕⊕◯ [e] Moderate
CHX chip	-	-	-0.76 (-1.28 to -0.24)	⊕⊕⊕◯ [e] Moderate	-0.76 (-1.28 to -0.24)	⊕⊕⊕◯ [e] Moderate
CHX xanthan	-	-	-1.42 (-2.87 to 0.03)	⊕⊕◯◯ [e, c] Low	-1.42 (-2.87 to 0.03)	⊕◯◯◯ [c, f] Very Low
DH	-	-	-0.73 (-1.31 to -0.15)	⊕⊕⊕◯ [e] Moderate	-0.73 (-1.31 to -0.15)	⊕⊕⊕◯ [e] Moderate
MET	-	-	-0.36 (-1.18 to 0.47)	⊕⊕◯◯ [e, c] Low	-0.36 (-1.18 to 0.47)	⊕◯◯◯ [c, f] Very Low
MINO gel	-	-	-0.34 (-1.52 to 0.84)	⊕⊕◯◯ [e, c] Low	-0.34 (-1.52 to 0.84)	⊕◯◯◯ [c, f] Very Low

MINO microspheres	-	-	-0.21 (-0.73 to 0.31)	⊕⊕◯◯ [e, c] Low	-0.21 (-0.73 to 0.31)	⊕◯◯◯ [c, f] Very Low
placebo	-	-	-0.97 (-1.59 to -0.34)	⊕⊕⊕◯ [e] Moderate	-0.97 (-1.59 to -0.34)	⊕⊕⊕◯ [e] Moderate

**SA**	**-**	**-**	**0.23 (- 0.41 to 0.88)**	**⊕⊕◯◯ [e, c] Low**	**0.23 (- 0.41 to 0.88)**	**⊕⊕◯◯ [e, c] Low**
SMD	-0.86 (-1.29 to -0.44)	⊕⊕⊕◯ [a] Moderate	-	-	-0.86 (-1.29 to -0.44)	⊕⊕⊕◯ [e] Moderate
**aPDT MB 0.005% vs.**						
aPDT MB 1%	-	-	-0.58 (-0.99 to -0.18)	⊕⊕⊕◯ [e] Moderate	-0.58 (-0.99 to -0.18)	⊕⊕◯◯ [e, c] Low
aPDT TBO	-	-	-0.97 (-2.15 to 0.22)	⊕⊕◯◯ [e, c] Low	-0.97 (-2.15 to 0.22)	⊕⊕◯◯ [e, c] Low
CHX chip	-	-	-0.41 (-0.87 to 0.05)	⊕⊕◯◯ [e, c] Low	-0.41 (-0.87 to 0.05)	⊕◯◯◯ [c, f] Very Low
CHX xanthan	-	-	-1.07 (-2.49 to 0.36)	⊕⊕◯◯ [e, c] Low	-1.07 (-2.49 to 0.36)	⊕◯◯◯ [c, f] Very Low
DH	-	-	-0.37 (-0.90 to 0.15)	⊕⊕◯◯ [e, c] Low	-0.37 (-0.90 to 0.15)	⊕◯◯◯ [c, f] Very Low
MET	-	-	-0.00 (-0.79 to 0.78)	⊕⊕◯◯ [e, c] Low	-0.00 (-0.79 to 0.78)	⊕◯◯◯ [c, f] Very Low
MINO gel	-	-	0.01 (-1.14 to 1.16)	⊕⊕◯◯ [e, c] Low	0.01 (-1.14 to 1.16)	⊕◯◯◯ [c, f] Very Low
MINO microspheres	-	-	0.14 (- 0.32 to 0.60)	⊕⊕◯◯ [e, c] Low	0.14 (- 0.32 to 0.60)	⊕◯◯◯ [c, f] Very Low
placebo	-0.50 (-0.86 to -0.14)	⊕⊕⊕◯ [a] Moderate	-0.60 (-1.20 to 0.01)	⊕⊕◯◯ [e, c] Low	-0.61 (-1.16 to -0.06)	⊕⊕⊕◯ [e] Moderate
SA	-	-	0.59 (0.02 to 1.16)	⊕⊕⊕◯ [e] Moderate	0.59 (0.02 to 1.16)	⊕⊕◯◯ [e, c] Low
SMD	-0.70 (-2.02 to 0.62)	⊕⊕◯◯ [a, c] Low	-0.60 (-2.01 to 0.80)	⊕⊕◯◯ [e, c] Low	-0.51 (-0.85 to -0.16)	⊕⊕◯◯ [e, c] Low
**aPDT MB 1% vs.**						
aPDT TBO	-	-	-0.38 (-1.53 to 0.77)	⊕⊕◯◯ [e, c] Low	-0.38 (-1.53 to 0.77)	⊕◯◯◯ [c, f] Very Low

CHX chip	-	-	0.18 (- 0.20 to 0.55)	⊕⊕◯◯ [e, c] Low	0.18 (- 0.20 to 0.55)	⊕◯◯◯ [c, f] Very Low
CHX xanthan	-	-	-0.48 (-1.89 to 0.92)	⊕⊕◯◯ [e, c] Low	-0.48 (-1.89 to 0.92)	⊕◯◯◯ [c, f] Very Low
DH	-	-	0.21 (-0.24 to 0.66)	⊕⊕◯◯ [e, c] Low	0.21 (-0.24 to 0.66)	⊕◯◯◯ [c, f] Very Low
MET	-	-	0.58 (-0.16 to 1.32)	⊕⊕◯◯ [e, c] Low	0.58 (-0.16 to 1.32)	⊕◯◯◯ [c, f] Very Low
MINO gel	-	-	0.60 (-0.52 to 1.72)	⊕⊕◯◯ [e, c] Low	0.60 (-0.52 to 1.72)	⊕◯◯◯ [c, f] Very Low
MINO microspheres	-	-	0.73 (0.35 to 1.10)	⊕⊕⊕◯ [e] Moderate	0.73 (0.35 to 1.10)	⊕⊕⊕◯ [e] Moderate
placebo	0.04 (-0.45 to 0.53)	⊕⊕◯◯ [a, c] Low	0.01 (-0.89 to 0.90)	⊕⊕◯◯ [e, c] Low	-0.03 (-0.46 to 0.40)	⊕◯◯◯ [c, f] Very Low
SA	-	-	1.17 (-0.71 to 1.63)	⊕⊕◯◯ [e, c] Low	1.17 (-0.71 to 1.63)	⊕◯◯◯ [c, f] Very Low
SMD	-0.08 (-0.30 to 0.14)	⊕⊕◯◯ [a, c] Low	0.03 (-0.96 to 1.03)	⊕⊕◯◯ [e, c] Low	0.08 (-0.14 to 0.30)	⊕◯◯◯ [c, f] Very Low
**aPDT TBO vs.**						
CHX chip	-	-	0.56 (- 0.62 to 1.74)	⊕⊕◯◯ [e, c] Low	0.56 (- 0.62 to 1.74)	⊕◯◯◯ [c, f] Very Low
CHX xanthan	-	-	-0.10 (-1.89 to 1.69)	⊕⊕◯◯ [e, c] Low	-0.10 (-1.89 to 1.69)	⊕◯◯◯ [c, f] Very Low
DH	-	-	0.60 (- 0.61 to 1.80)	⊕⊕◯◯ [e, c] Low	0.60 (- 0.61 to 1.80)	⊕◯◯◯ [c, f] Very Low
MET	-	-	0.96 (-0.38 to 2.30)	⊕⊕◯◯ [e, c] Low	0.96 (-0.38 to 2.30)	⊕◯◯◯ [c, f] Very Low
MINO gel	-	-	0.98 (- 0.60 to 2.56)	⊕⊕◯◯ [e, c] Low	0.98 (- 0.60 to 2.56)	⊕◯◯◯ [c, f] Very Low
MINO microspheres	-	-	1.11 (-0.07 to 2.29)	⊕⊕◯◯ [e, c] Low	1.11 (-0.07 to 2.29)	⊕◯◯◯ [c, f] Very Low
placebo	-0.43 (-1.77 to 0.91)	⊕⊕◯◯ [a, c] Low	0.12 (-2.22 to 2.47)	⊕◯◯◯ [d, f] Very Low	0.35 (-0.81 to 1.52)	⊕◯◯◯ [c, f] Very Low
SA	-	-	1.55 (0.38 to 2.73)	⊕⊕⊕◯ [e] Moderate	1.55 (0.38 to 2.73)	⊕⊕⊕◯ [e] Moderate
SMD	-0.42 (-1.61 to 0.77)	⊕⊕◯◯ [a, c] Low	0.88 (-2.93 to 4.68)	⊕◯◯◯ [d, f] Very Low	0.46 (-0.68 to 1.60)	⊕◯◯◯ [c, f] Very Low

**CHX chip vs.**						
CHX xanthan	-	-	-0.66 (-2.08 to 0.76)	⊕⊕◯◯ [e, c]<br>Low	-0.66 (-2.08 to 0.76)	⊕◯◯◯ [c, f]<br>Very Low
DH	-	-	0.04 (-0.46 to 0.53)	⊕⊕◯◯ [e, c]<br>Low	0.04 (-0.46 to 0.53)	⊕◯◯◯ [c, f]<br>Very Low
MET	-	-	0.40 (-0.37 to 1.17)	⊕⊕◯◯ [e, c]<br>Low	0.40 (-0.37 to 1.17)	⊕◯◯◯ [c, f]<br>Very Low
MINO gel	-	-	0.42 (-0.72 to 1.56)	⊕⊕◯◯ [e, c]<br>Low	0.42 (-0.72 to 1.56)	⊕◯◯◯ [c, f]<br>Very Low
MINO microspheres	-	-	0.55 (0.12 to 0.98)	⊕⊕⊕◯ [e]<br>Moderate	0.55 (0.12 to 0.98)	⊕⊕⊕◯ [e]<br>Moderate
placebo	-	-	-0.21 (-0.76 to 0.34)	⊕⊕◯◯ [e, c]<br>Low	-0.21 (-0.76 to 0.34)	⊕◯◯◯ [c, f]<br>Very Low
SA	-	-	0.99 (0.42 to 1.56)	⊕⊕⊕◯ [e]<br>Moderate	0.99 (0.42 to 1.56)	⊕⊕⊕◯ [e]<br>Moderate
SMD	-0.10 (-0.40 to 0.20)	⊕⊕◯◯ [a, c]<br>Low	-	-	-0.10 (-0.40 to 0.20)	⊕◯◯◯ [c, f]<br>Very Low
**CHX xanthan vs.**						
DH	-	-	0.69 (-0.75 to 2.14)	⊕⊕◯◯ [e, c]<br>Low	0.69 (-0.75 to 2.14)	⊕◯◯◯ [c, f]<br>Very Low
MET	-	-	1.06 (-0.49 to 2.62)	⊕⊕◯◯ [e, c]<br>Low	1.06 (-0.49 to 2.62)	⊕◯◯◯ [c, f]<br>Very Low
MINO gel	-	-	1.08 (-0.69 to 2.85)	⊕⊕◯◯ [e, c]<br>Low	1.08 (-0.69 to 2.85)	⊕◯◯◯ [c, f]<br>Very Low
MINO microspheres	-	-	1.21 (-0.21 to 2.63)	⊕⊕◯◯ [e, c]<br>Low	1.21 (-0.21 to 2.63)	⊕◯◯◯ [c, f]<br>Very Low
placebo	-	-	0.45 (-1.01 to 1.91)	⊕⊕◯◯ [e, c]<br>Low	0.45 (-1.01 to 1.91)	⊕◯◯◯ [c, f]<br>Very Low
SA	-	-	1.65 (0.18 to 3.12)	⊕⊕⊕◯ [e]<br>Moderate	1.65 (0.18 to 3.12)	⊕⊕⊕◯ [e]<br>Moderate
SMD	-0.56 (-1.95 to 0.83)	⊕⊕◯◯ [a, c]<br>Low	-	-	0.56 (-0.83 to 1.95)	⊕◯◯◯ [c, f]<br>Very Low
**DH vs.**						
MET	-	-	0.37 (-0.44 to 1.18)	⊕⊕◯◯ [e, c]<br>Low	0.37 (-0.44 to 1.18)	⊕◯◯◯ [c, f]<br>Very Low
MINO gel	-	-	0.38 (-0.78 to 1.55)	⊕⊕◯◯ [e, c]<br>Low	0.38 (-0.78 to 1.55)	⊕◯◯◯ [c, f]<br>Very Low

MINO microspheres	-	-	0.51 (0.02 to 1.01)	⊕⊕⊕◯ [e]<br>Moderate	0.51 (0.02 to 1.01)	⊕⊕⊕◯ [e]<br>Moderate
placebo	-	-	-0.24 (-0.85 to 0.36)	⊕⊕◯◯ [e, c]<br>Low	-0.24 (-0.85 to 0.36)	⊕◯◯◯ [c, f]<br>Very Low
SA	-	-	0.96 (0.33 to 1.58)	⊕⊕⊕◯ [e]<br>Moderate	0.96 (0.33 to 1.58)	⊕⊕⊕◯ [e]<br>Moderate
SMD	-0.13 (-0.53 to 0.26)	⊕⊕◯◯ [a, c]<br>Low	-	-	-0.14 (-0.53 to 0.26)	⊕◯◯◯ [c, f]<br>Very Low
**MET vs.**						
MINO gel	-	-	0.02 (-1.29 to 1.32)	⊕⊕◯◯ [e, c]<br>Low	0.02 (-1.29 to 1.32)	⊕◯◯◯ [c, f]<br>Very Low
MINO microspheres	-	-	0.15 (-0.62 to 0.92)	⊕⊕◯◯ [e, c]<br>Low	0.15 (-0.62 to 0.92)	⊕◯◯◯ [c, f]<br>Very Low
placebo	-	-	-0.61 (-1.45 to 0.23)	⊕⊕◯◯ [e, c]<br>Low	-0.61 (-1.45 to 0.23)	⊕◯◯◯ [c, f]<br>Very Low
SA	-	-	0.59 (-0.27 to 1.45)	⊕⊕◯◯ [e, c]<br>Low	0.59 (-0.27 to 1.45)	⊕◯◯◯ [c, f]<br>Very Low
SMD	-0.50 (-1.21 to 0.20)	⊕⊕◯◯ [a, c]<br>Low	-	-	-0.50 (-1.21 to 0.20)	⊕◯◯◯ [c, f]<br>Very Low
**MINO gel vs.**						
MINO microspheres			0.13 (-1.01 to 1.27)	⊕⊕◯◯ [e, c]<br>Low	0.13 (-1.01 to 1.27)	⊕◯◯◯ [c, f]<br>Very Low
placebo	-	-	-0.63 (-1.82 to 0.56)	⊕⊕◯◯ [e, c]<br>Low	-0.63 (-1.82 to 0.56)	⊕◯◯◯ [c, f]<br>Very Low
SA	-	-	0.57 (-0.63 to 1.77)	⊕⊕◯◯ [e, c]<br>Low	0.57 (-0.63 to 1.77)	⊕◯◯◯ [c, f]<br>Very Low
SMD	-0.52 (-1.62 to 0.58)	⊕⊕◯◯ [a, c]<br>Low	-	-	-0.52 (-1.62 to 0.58)	⊕◯◯◯ [c, f]<br>Very Low
**MINO microspheres vs.**						
placebo	-	-	-0.76 (-1.31 to -0.21)	⊕⊕⊕◯ [e]<br>Moderate	-0.76 (-1.31 to -0.21)	⊕⊕⊕◯ [e]<br>Moderate
SA	-	-	0.44 (-0.13 to 1.01)	⊕⊕◯◯ [e, c]<br>Low	0.44 (-0.13 to 1.01)	⊕◯◯◯ [c, f]<br>Very Low
SMD	-0.65 (-0.95 to -0.35)	⊕⊕⊕◯ [a]<br>Moderate	-	-	-0.65 (-0.95 to -0.35)	⊕⊕⊕◯ [e]<br>Moderate
**Placebo vs.**						

SA	-1.20 (-1.35 to -1.05)	⊕⊕⊕◯ [a]<br>Moderate	-	-	1.20 (1.05 to 1.35)	⊕⊕⊕◯ [e]<br>Moderate
SMD	0.01 (-1.11 to 1.13)	⊕⊕◯◯ [a, c]<br>Low	0.13 (-0.37 to 0.63)	⊕⊕◯◯ [e, c]<br>Low	0.11 (-0.35 to 0.57)	⊕◯◯◯ [c, f]<br>Very Low
**SA vs. SMD**	-	-	-1.09 (-1.58 to -0.61)	⊕⊕⊕◯ [e]<br>Moderate	-1.09 (-1.58 to -0.61)	⊕⊕⊕◯ [e]<br>Moderate

[a] Limitations (risk of bias). [b] Inconsistency. [c] Imprecision. [d] Severe imprecision. [e] Contributing evidence of moderate quality. [f] Contributing evidence of low or very low quality. [g] Incoherence

Mean differences (95% CIs) and certainty of evidence for CAL changes in parallel design

**Comparisons**	**GRADE**					
	**Direct evidence**		**Indirect evidence**		**Network meta-analysis**	
	**Mean difference (95% CI)**	**Certainty of evidence**	**Mean difference (95% CI)**	**Certainty of evidence**	**Mean difference (95% CI)**	**Certainty of evidence**
**aPDT MB 1% vs.**						
aPDT PC	-	-	-0.19 (-0.77 to 0.39)	⊕⊕◯◯ [e, c]<br>Low	-0.19 (-0.77 to 0.39)	⊕◯◯◯ [c, f]<br>Very Low
CHX chip	-	-	0.11 (-0.66 to 0.88)	⊕⊕◯◯ [e, c]<br>Low	0.11 (-0.66 to 0.88)	⊕◯◯◯ [c, f]<br>Very Low
CHX xanthan	-	-	-0.23 (-1.05 to 0.60)	⊕⊕◯◯ [e, c]<br>Low	-0.23 (-1.05 to 0.60)	⊕◯◯◯ [c, f]<br>Very Low
DH	-	-	0.45 (-0.27 to 1.17)	⊕⊕◯◯ [e, c]<br>Low	0.45 (-0.27 to 1.17)	⊕◯◯◯ [c, f]<br>Very Low
MINO gel	-	-	0.30 (-0.32 to 0.92)	⊕⊕◯◯ [e, c]<br>Low	0.30 (-0.32 to 0.92)	⊕◯◯◯ [c, f]<br>Very Low
MINO microspheres	-	-	-0.26 (-0.78 to 0.27)	⊕⊕◯◯ [e, c]<br>Low	-0.26 (-0.78 to 0.27)	⊕◯◯◯ [c, f]<br>Very Low
PI 10%	-	-	-0.84 (-1.79 to 0.11)	⊕⊕◯◯ [e, c]<br>Low	-0.84 (-1.79 to 0.11)	⊕◯◯◯ [c, f]<br>Very Low
placebo	0.22 (-0.85 to 1.29)	⊕⊕◯◯ [a, c]<br>Low	-0.61 (-1.21 to -0.02)	⊕⊕⊕◯ [e]<br>Moderate	-0.42 (-0.94 to 0.10)	⊕⊕◯◯ [e, c]<br>Low
SMD	-0.55 (-1.09 to -0.01)	⊕⊕◯◯ [a, c]<br>Low	0.28 (-0.81 to 1.38)	⊕⊕◯◯ [e, c]<br>Low	-0.39 (-0.87 to 0.09)	⊕⊕◯◯ [e, c]<br>Low
TC	-	-	-0.46 (-1.46 to 0.54)	⊕⊕◯◯ [e, c]<br>Low	-0.46 (-1.46 to 0.54)	⊕◯◯◯ [c, f]<br>Very Low

**aPDT PC vs.**						
CHX chip	-	-	0.30 (-0.39 to 0.99)	⊕⊕◯◯ [e, c]<br>Low	0.30 (-0.39 to 0.99)	⊕◯◯◯ [c, f]<br>Very Low
CHX xanthan	-	-	-0.04 (-0.80 to 0.72)	⊕⊕◯◯ [e, c]<br>Low	-0.04 (-0.80 to 0.72)	⊕◯◯◯ [c, f]<br>Very Low
DH	-	-	0.64 (-0.01 to 1.29)	⊕⊕⊕◯ [e]<br>Moderate	0.64 (-0.01 to 1.29)	⊕⊕⊕◯ [e]<br>Moderate
MINO gel	-	-	0.49 (-0.02 to 1.00)	⊕⊕◯◯ [e, c]<br>Low	0.49 (-0.02 to 1.00)	⊕◯◯◯ [c, f]<br>Very Low
MINO microspheres	-	-	-0.07 (-0.47 to 0.34)	⊕⊕◯◯ [e, c]<br>Low	-0.07 (-0.47 to 0.34)	⊕◯◯◯ [c, f]<br>Very Low
PI 10%	-	-	-0.65 (-1.53 to 0.23)	⊕⊕◯◯ [e, c]<br>Low	-0.65 (-1.53 to 0.23)	⊕◯◯◯ [c, f]<br>Very Low
placebo	-	-	-0.23 (-0.64 to 0.18)	⊕⊕◯◯ [e, c]<br>Low	-0.23 (-0.64 to 0.18)	⊕◯◯◯ [c, f]<br>Very Low
SMD	-0.20 (-0.53 to 0.13)	⊕⊕◯◯ [a, c]<br>Low	-	-	-0.20 (-0.53 to 0.13)	⊕◯◯◯ [c, f]<br>Very Low
TC			-0.27 (-1.21 to 0.66)	⊕⊕◯◯ [e, c]<br>Low	-0.27 (-1.21 to 0.66)	⊕◯◯◯ [c, f]<br>Very Low
**CHX chip vs.**						
CHX xanthan	-	-	-0.34 (-1.25 to 0.58)	⊕⊕◯◯ [e, c]<br>Low	-0.34 (-1.25 to 0.58)	⊕◯◯◯ [c, f]<br>Very Low
DH	-	-	0.34 (-0.48 to 1.16)	⊕⊕◯◯ [e, c]<br>Low	0.34 (-0.48 to 1.16)	⊕◯◯◯ [c, f]<br>Very Low
MINO gel	-	-	0.19 (-0.53 to 0.91)	⊕⊕◯◯ [e, c]<br>Low	0.19 (-0.53 to 0.91)	⊕◯◯◯ [c, f]<br>Very Low
MINO microspheres	-	-	-0.37 (-1.02 to 0.28)	⊕⊕◯◯ [e, c]<br>Low	-0.37 (-1.02 to 0.28)	⊕◯◯◯ [c, f]<br>Very Low
PI 10%	-	-	-0.95 (-1.97 to 0.07)	⊕⊕◯◯ [e, c]<br>Low	-0.95 (-1.97 to 0.07)	⊕◯◯◯ [c, f]<br>Very Low
placebo	-	-	-0.53 (-1.18 to 0.12)	⊕⊕◯◯ [e, c]<br>Low	-0.53 (-1.18 to 0.12)	⊕◯◯◯ [c, f]<br>Very Low
SMD	-0.50 (-1.10 to 0.10)	⊕⊕◯◯ [a, c]<br>Low	-	-	-0.50 (-1.10 to 0.10)	⊕◯◯◯ [c, f]<br>Very Low
TC	-1.00 (-2.00 to 0.00)	⊕⊕◯◯ [a, c]<br>Low	1.40 (-0.748 to 3.54)	⊕◯◯◯ [d, f]<br>Very Low	-0.57 (-1.48 to 0.33)	⊕◯◯◯ [c, f]<br>Very Low
**CHX xanthan vs.**						

DH	-	-	0.68 (-0.14 to 1.50)	⊕⊕◯◯ [e, c] Low	0.68 (-0.14 to 1.50)	⊕◯◯◯ [c, f] Very Low
MINO gel	-	-	0.53 (-0.26 to 1.32)	⊕⊕◯◯ [e, c] Low	0.53 (-0.26 to 1.32)	⊕◯◯◯ [c, f] Very Low
MINO microspheres	-	-	-0.03 (-0.72 to 0.66)	⊕⊕◯◯ [e, c] Low	-0.03 (-0.72 to 0.66)	⊕◯◯◯ [c, f] Very Low
PI 10%	-	-	-0.61 (-1.68 to 0.46)	⊕⊕◯◯ [e, c] Low	-0.61 (-1.68 to 0.46)	⊕◯◯◯ [c, f] Very Low
placebo	-0.14 (-0.62 to 0.34)	⊕⊕◯◯ [a, c] Low	-	-	-0.19 (-0.83 to 0.45)	⊕◯◯◯ [c, f] Very Low
SMD	-	-	-0.16 (-0.85 to 0.53)	⊕⊕◯◯ [e, c] Low	-0.16 (-0.85 to 0.53)	⊕◯◯◯ [c, f] Very Low
TC	-	-	-0.23 (-1.35 to 0.88)	⊕⊕◯◯ [e, c] Low	-0.23 (-1.35 to 0.88)	⊕◯◯◯ [c, f] Very Low
**DH vs.**						
MINO gel	-	-	-0.15 (-0.83 to 0.53)	⊕⊕◯◯ [e, c] Low	-0.15 (-0.83 to 0.53)	⊕◯◯◯ [c, f] Very Low
MINO microspheres	-	-	-0.71 (-1.27 to -0.15)	⊕⊕⊕◯ [e] Moderate	-0.71 (-1.27 to -0.15)	⊕⊕⊕◯ [e] Moderate
PI 10%	-	-	-1.29 (-2.28 to -0.30)	⊕⊕◯◯ [e, c] Low	-1.29 (-2.28 to -0.30)	⊕◯◯◯ [c, f] Very Low
placebo	-0.87 (-1.37 to -0.37)	⊕⊕⊕◯ [a] Moderate	-	-	-0.87 (-1.37 to -0.37)	⊕⊕⊕◯ [e] Moderate
SMD	-	-	-0.84 (-1.40 to -0.28)	⊕⊕⊕◯ [e] Moderate	-0.84 (-1.40 to -0.28)	⊕⊕⊕◯ [e] Moderate
TC	-	-	-0.91 (-1.95 to 0.13)	⊕◯◯◯ [d, f] Very Low	-0.91 (-1.95 to 0.13)	⊕◯◯◯ [c, f] Very Low
**MINO gel vs.**						
MINO microspheres	-	-	-0.56 (-1.01 to -0.10)	⊕⊕⊕◯ [e] Moderate	-0.56 (-1.01 to -0.10)	⊕⊕⊕◯ [e] Moderate
PI 10%	-	-	-1.14 (-2.05 to -0.23)	⊕⊕◯◯ [e, c] Low	-1.14 (-2.05 to -0.23)	⊕◯◯◯ [c, f] Very Low
placebo	-	-	-0.72 (-1.18 to -0.26)	⊕⊕⊕◯ [e] Moderate	-0.72 (-1.18 to -0.26)	⊕⊕⊕◯ [e] Moderate
SMD	-0.69 (-1.08 to -0.30)	⊕⊕⊕◯ [a] Moderate	-	-	-0.69 (-1.08 to -0.30)	⊕⊕⊕◯ [e] Moderate

TC	-	-	-0.76 (-1.72 to 0.19)	⊕⊕◯◯ [e, c]<br>Low	-0.76 (-1.72 to 0.19)	⊕◯◯◯ [c, f]<br>Very Low
**MINO microspheres vs.**						
PI 10%	-	-	-0.58 (-1.44 to 0.27)	⊕⊕◯◯ [e, c]<br>Low	-0.58 (-1.44 to 0.27)	⊕◯◯◯ [c, f]<br>Very Low
placebo	-0.15 (-0.41 to 0.10)	⊕⊕◯◯ [a, c]<br>Low	-0.34 (-1.49 to 0.81)	⊕⊕◯◯ [e, c]<br>Low	-0.16 (-0.41 to 0.09)	⊕◯◯◯ [c, f]<br>Very Low
SMD	-0.12 (-0.36 to 0.12)	⊕⊕◯◯ [a, c]<br>Low	-1.79 (-4.22 to 0.65)	⊕◯◯◯ [d, f]<br>Very Low	-0.13 (-0.37 to 0.11)	⊕◯◯◯ [c, f]<br>Very Low
TC	-	-	-0.21 (-1.11 to 0.70)	⊕⊕◯◯ [e, c]<br>Low	-0.21 (-1.11 to 0.70)	⊕◯◯◯ [c, f]<br>Very Low
**PI 10% vs.**						
placebo	-	-	0.42 (-0.43 to 1.28)	⊕⊕◯◯ [e, c]<br>Low	0.42 (-0.43 to 1.28)	⊕◯◯◯ [c, f]<br>Very Low
SMD	-0.45 (-1.27 to 0.37)	⊕⊕◯◯ [a, c]<br>Low	-	-	0.45 (-0.37 to 1.27)	⊕◯◯◯ [c, f]<br>Very Low
TC	-	-	0.38 (-0.82 to 1.58)	⊕⊕◯◯ [e, c]<br>Low	0.38 (-0.82 to 1.58)	⊕◯◯◯ [c, f]<br>Very Low
**Placebo vs.**						
SMD	-0.09 (-0.35 to 0.16)	⊕⊕◯◯ [a, c]<br>Low	-0.73 (-1.62 to 0.16)	⊕⊕◯◯ [e, c]<br>Low	0.03 (-0.22 to 0.27)	⊕◯◯◯ [c, f]<br>Very Low
TC	-	-	-0.04 (-0.95 to 0.87)	⊕⊕◯◯ [e, c]<br>Low	-0.04 (-0.95 to 0.87)	⊕◯◯◯ [c, f]<br>Very Low
**TC vs. SMD**	-0.25 (-1.18 to 0.68)	⊕⊕◯◯ [a, c]<br>Low	2.53 (-0.04 to 5.10)	⊕◯◯◯ [d, f]<br>Very Low	0.07 (-0.80 to 0.95)	⊕◯◯◯ [c, f]<br>Very Low

[a] Limitations (risk of bias). [b] Inconsistency. [c] Imprecision. [d] Severe imprecision. [e] Contributing evidence of moderate quality. [f] Contributing evidence of low or very low quality. [g] Incoherence

Table S9. Subgroup analyses of specific parameters for included studies.

Variables	Number of studies	Number of patients		PPD				CAL			
		Control	Test	MD	95% CI	I^2 (%)	*p*-value	MD	95% CI	I^2 (%)	*p*-value
**Split mouth studies**											
**Duration**							**0.002**				**< 0.0001**
Medium	15	388	388	0.2	0.21; 0.63	68.5		0.29	0.08; 0.51	54.7	
Long	1	36	36	0.93	0.69; 1.17	-		1.20	1.05; 1.35	-	
**Smoking status**							**0.01**				0.20
Smoker	3	135	135	0.87	0.61; 1.12	0		0.63	0.35; 0.92	0	
Nonsmoker	13	289	289	0.41	0.18; 0.65	75.1		0.32	-0.07; 0.71	90.3	
**Treatment phase**							0.34				0.50
Untreated	14	376	376	0.48	0.25; 0.71	76.7		0.34	-0.01; 0.69	89.5	
Recurrent	2	48	48	0.22	-0.26; 0.71	0		0.64	-0.15; 1.42	0	
**Treatment area**							0.28				0.43
Partial mouth	12	354	354	0.39	0.15; 0.62	69.9		0.31	-0.13; 0.74	90.6	
Full mouth	4	70	70	0.68	0.20; 1.16	80.2		0.54	0.16; 0.91	56.7	
**Debridement method**							0.93				0.62
SRP	15	397	397	0.46	0.24; 0.68	75.8		0.36	0.02; 0.69	88.7	
U/S	1	27	27	0.50	-0.37; 1.37	-		0.70	-0.62; 2.02	-	
**Control group**							0.79				0.47
SMD	13	337	337	0.44	0.21; 0.68	71		0.31	0.07; 0.54	59.7	
Placebo	3	87	87	0.53	-0.09; 1.15	84.7		0.66	-0.26; 1.59	89.9	
**Funding source**							0.97				0.70
Commercial	3	135	135	0.47	-0.07; 1.01	0		0.50	-0.20; 1.20	0	
Noncommercial	13	289	289	0.46	0.22; 0.69	79.2		0.35	-0.01; 0.70	90.3	
**Parallel studies**											
**Duration**							0.50				**0.04**
Medium	12	460	453	0.28	0.10; 0.47	62.1		0.28	0.08; 0.48	55.8	
Long	1	16	16	0.62	-0.34; 1.58	-		1.25	0.32; 2.18	-	
**Smoking status**							0.27				0.32
Smoker	7	339	337	0.21	0.01; 0.41	57.3		0.22	0.02; 0.43	49.1	
Nonsmoker	6	137	132	0.44	0.10; 0.77	56.9		0.47	0.04; 0.89	60	
Treatment phase							0.13				0.56
Untreated	11	453	447	0.34	0.14; 0.54	61.2		0.33	0.09; 0.57	65.3	
Recurrent	2	23	22	0.07	-0.22; 0.36	0		0.20	-0.20; 0.59	0	
**Treatment area**							0.82				0.32

Partial mouth	6	115	109	0.26	-0.06; 0.58	50.2		0.47	0.08; 0.86	48.6	
Full mouth	7	361	360	0.31	0.08; 0.50	68.7		0.23	-0.00; 0.47	62.4	
**Debridement method**							0.10				0.05
SRP	12	462	455	0.32	0.14; 0.51	58.1		0.35	0.15; 0.55	56.9	
U/S	1	14	14	-0.08	-0.53; 0.37	-		-0.45	-1.24; 0.34	-	
**Control group**							0.71				0.75
SMD	10	414	413	0.27	0.07; 0.48	62.5		0.28	0.06; 0.50	57.9	
Placebo	3	62	56	0.37	-0.09; 0.82	61.2		0.39	-0.23; 1.01	63.2	
**Funding source**							**0.03**				**0.02**
Commercial	6	310	308	0.16	0.03; 0.30	0		0.11	-0.05; 0.26	0	
Noncommercial	7	166	161	0.49	0.23; 0.76	52.4		0.53	0.21; 0.85	53.9	
